# Evaluating the Impact of Chemical Complexity and Horizontal Resolution on Tropospheric Ozone Over the Conterminous US With a Global Variable Resolution Chemistry Model

**DOI:** 10.1029/2021MS002889

**Published:** 2022-06-22

**Authors:** Rebecca H. Schwantes, Forrest G. Lacey, Simone Tilmes, Louisa K. Emmons, Peter H. Lauritzen, Stacy Walters, Patrick Callaghan, Colin M. Zarzycki, Mary C. Barth, Duseong S. Jo, Julio T. Bacmeister, Richard B. Neale, Francis Vitt, Erik Kluzek, Behrooz Roozitalab, Samuel R. Hall, Kirk Ullmann, Carsten Warneke, Jeff Peischl, Ilana B. Pollack, Frank Flocke, Glenn M. Wolfe, Thomas F. Hanisco, Frank N. Keutsch, Jennifer Kaiser, Thao Paul V. Bui, Jose L. Jimenez, Pedro Campuzano‐Jost, Eric C. Apel, Rebecca S. Hornbrook, Alan J. Hills, Bin Yuan, Armin Wisthaler

**Affiliations:** ^1^ Atmospheric Chemistry Observations & Modeling Laboratory National Center for Atmospheric Research Boulder CO USA; ^2^ Cooperative Institute for Research in Environmental Sciences University of Colorado Boulder Boulder CO USA; ^3^ Chemical Sciences Laboratory National Oceanic and Atmospheric Administration Boulder CO USA; ^4^ Climate and Global Dynamics Laboratory National Center for Atmospheric Research Boulder CO USA; ^5^ Department of Meteorology and Atmospheric Science Pennsylvania State University University Park PA USA; ^6^ Department of Chemical and Biochemical Engineering The University of Iowa Iowa City IA USA; ^7^ Center for Global and Regional Environmental Research The University of Iowa Iowa City IA USA; ^8^ Department of Atmospheric Science Colorado State University Fort Collins CO USA; ^9^ Atmospheric Chemistry and Dynamics Lab NASA Goddard Space Flight Center Greenbelt MD USA; ^10^ John A. Paulson School of Engineering and Applied Sciences Harvard University Cambridge MA USA; ^11^ Department of Chemistry and Chemical Biology Harvard University Cambridge MA USA; ^12^ Department of Earth and Planetary Sciences Harvard University Cambridge MA USA; ^13^ School of Civil and Environmental Engineering Georgia Institute of Technology Atlanta GA USA; ^14^ School of Earth and Atmospheric Sciences Georgia Institute of Technology Atlanta GA USA; ^15^ Earth Science Division NASA Ames Research Center Moffett Field CA USA; ^16^ Department of Chemistry University of Colorado Boulder CO USA; ^17^ Institute for Environmental and Climate Research Jinan University Guangzhou China; ^18^ Institute for Ion Physics and Applied Physics University of Innsbruck Innsbruck Austria; ^19^ Department of Chemistry University of Oslo Oslo Norway

**Keywords:** atmospheric chemistry, tropospheric ozone, air quality, aircraft campaigns, horizontal resolution, chemical complexity

## Abstract

A new configuration of the Community Earth System Model (CESM)/Community Atmosphere Model with full chemistry (CAM‐chem) supporting the capability of horizontal mesh refinement through the use of the spectral element (SE) dynamical core is developed and called CESM/CAM‐chem‐SE. Horizontal mesh refinement in CESM/CAM‐chem‐SE is unique and novel in that pollutants such as ozone are accurately represented at human exposure relevant scales while also directly including global feedbacks. CESM/CAM‐chem‐SE with mesh refinement down to ∼14 km over the conterminous US (CONUS) is the beginning of the Multi‐Scale Infrastructure for Chemistry and Aerosols (MUSICAv0). Here, MUSICAv0 is evaluated and used to better understand how horizontal resolution and chemical complexity impact ozone and ozone precursors over CONUS as compared to measurements from five aircraft campaigns, which occurred in 2013. This field campaign analysis demonstrates the importance of using finer horizontal resolution to accurately simulate ozone precursors such as nitrogen oxides and carbon monoxide. In general, the impact of using more complex chemistry on ozone and other oxidation products is more pronounced when using finer horizontal resolution where a larger number of chemical regimes are resolved. Large model biases for ozone near the surface remain in the Southeast US as compared to the aircraft observations even with updated chemistry and finer horizontal resolution. This suggests a need for adding the capability of replacing sections of global emission inventories with regional inventories, increasing the vertical resolution in the planetary boundary layer, and reducing model biases in meteorological variables such as temperature and clouds.

## Key Points

- A new configuration of the Community Atmosphere Model with full chemistry supporting horizontal mesh refinement is developed
- This configuration is the beginning of the Multi‐Scale Infrastructure for Chemistry and Aerosols
- Updating chemistry and horizontal resolution improves simulated ozone and ozone precursors compared to aircraft observations

## 1 Introduction

Because ozone is a short‐lived climate forcer, negatively impacts human health, damages ecosystems, and is fundamental to atmospheric chemistry through its role in photooxidation processes (Monks et al., 2015), accurately predicting ozone in 3D models for the right reasons is important (Young et al., 2018). Generally, models have reasonable skill at simulating large‐scale spatial and seasonal features of tropospheric ozone, but biases exist (Young et al., 2018). For example, surface ozone is generally over‐predicted in models, especially in the northern hemisphere (Young et al., 2018). When comparing a wide variety of global models, more variability exists in the processes that determine the ozone distribution (e.g., chemical production and loss, deposition, and troposphere‐stratosphere exchange) than in the total tropospheric ozone burden (Young et al., 2018) suggesting a need for more evaluation and improvement of model physical and chemical processes. Over the conterminous US (CONUS) specifically, many atmospheric chemistry models consistently overpredict surface ozone during the summer in the Southeast US compared to observations (Canty et al., 2015; Emmons et al., 2020; Fiore et al., 2009; Im et al., 2015; Reidmiller et al., 2009; S. Yu et al., 2010; Tilmes et al., 2015). Many past studies have investigated this problem and suggested possible solutions ranging from improvements in the representation of temperature (Brown‐Steiner et al., 2015; Rasmussen et al., 2012), clouds (Ryu et al., 2018), anthropogenic emissions (McDonald, McKeen, et al., 2018; Travis et al., 2016), biogenic emissions (Kaiser et al., 2018), chemistry (Bates & Jacob, 2019; Schwantes et al., 2020; Squire et al., 2015; Vasquez et al., 2020; Zare et al., 2018, 2019), chemical solver (Sun et al., 2017), and deposition of ozone and volatile organic compounds (VOCs; Clifton et al., 2017; Karl et al., 2010; Nguyen et al., 2015; Travis & Jacob, 2019; Val Martin et al., 2014).

In many cases, global model simulations are not performed at horizontal and vertical resolutions fine enough to accurately represent the nonlinear processes responsible for ozone formation and loss. To fill this gap, regional models with the capability of performing simulations at finer horizontal resolution are used. These regional models require the use of lateral and upper boundary conditions (BCs), which are often either fixed or derived from a global model. Past studies (F. Yan et al., 2021; Herron‐Thorpe et al., 2012; Huang et al., 2017; Im et al., 2018; Tang et al., 2007, 2009) have identified that the selection of BCs impacts simulated ozone especially in regions near the boundaries of the regional model domain. Model performance is often enhanced by using BCs derived from a global model instead of fixed BCs to better capture the temporal variability of ozone and ozone precursors entering the regional model domain; however, general biases present in global models are transferred into regional models through BCs (Herron‐Thorpe et al., 2012; Tang et al., 2007, 2009). Because of this connection, in order to improve model skill at simulating ozone in a regional model, model performance needs to be improved in both regional and global models. Since the chemical and physical processes are often different between global and regional models, separately improving both models is time consuming and inefficient. Additionally, differences in the physical and chemical processes between the regional and global models can cause inconsistencies in the regional model results (Neal et al., 2017). This, along with recent reports summarizing the status and future direction of climate and Earth system modeling (Bellucci et al., 2015; NRC, 2012; Pfister et al., 2020), demonstrates the importance of efforts to unify regional and global models in order to seamlessly model across scales.

Capabilities to model atmospheric chemistry at varying scales exist in several models. For example, WRF‐Chem (Weather Research and Forecasting model coupled with Chemistry), which is a regional fully coupled meteorology‐chemistry model, uses nesting and relies on global model results for BCs (Fast et al., 2006; Grell et al., 2005). GEOS‐Chem, which is a global chemical transport model, uses nesting in their Classic version (Wang et al., 2004; Y.‐Y. Yan et al., 2014), but recently has developed a more seamless approach through grid‐stretching (Bindle et al., 2021). The Community Earth System Model/Community Atmosphere Model with chemistry (CESM/CAM‐chem) is an Earth system model with fully coupled tropospheric and stratospheric chemistry (Lamarque et al., 2012; Tilmes et al., 2015). This work will bring CESM/CAM‐chem to regional scales. The capability of horizontal mesh refinement is already supported in CESM/CAM through the use of the spectral element (SE) dynamical core (Baer et al., 2006; Fournier et al., 2004; Lauritzen et al., 2018; Zarzycki, Jablonowski, & Taylor, 2014; Zarzycki, Levy, et al., 2014). This work expands on this past work to add full tropospheric and stratospheric chemistry of gases and aerosols into CESM/CAM‐SE to create CESM/CAM‐chem‐SE. The capability in CESM/CAM‐chem‐SE to use grids with horizontal mesh refinement within an Earth system model with fully coupled tropospheric and stratospheric chemistry is a novel advancement. The regional and global model components are seamlessly connected such that direct feedbacks occur, which is ideal for accurately simulating air quality from global to regional scales. The capability of mesh refinement within a single model ensures consistency in the physical and chemical processes in the regional and global components. This framework also increases efficiency in model development such that an update to a model component can be easily incorporated into the entire system. The development of CESM/CAM‐chem‐SE is the beginning of a community‐wide effort called MUSICA, The Multi‐Scale Infrastructure for Chemistry and Aerosols, which will create a unified infrastructure to model atmospheric chemistry and aerosols across local to regional to global scales in the Earth system (Pfister et al., 2020). The capability of regional refinement down to ∼14 km over CONUS is called MUSICAv0 (https://www2.acom.ucar.edu/sections/musica-v0).

Better understanding the connections between air quality and climate (Monks et al., 2015) will be an important future use of CESM/CAM‐chem‐SE. Climate mitigation strategies through reductions in air pollutant precursors co‐emitted with greenhouse gases have the potential to greatly reduce air pollution, which will improve human health and potentially offset the economic costs associated with climate mitigation (Chang et al., 2017; Thompson et al., 2014). Many past studies have either used coarse horizontal resolution in a global model (e.g., Garcia‐Menendez et al., 2015; Stohl et al., 2015; Y. Lee et al., 2016) or relied on connecting results from inconsistent regional and global models (e.g., Colette et al., 2013; Gao et al., 2013; Kumar et al., 2018; Pfister et al., 2014) in order to answer how various climate mitigation strategies will impact air quality. CESM/CAM‐chem‐SE, which is a climate model and has fine enough horizontal resolution to simulate human health exposure to air pollutants, will provide a novel and more consistent tool to better study the connections between air quality and climate in the future.

Several past studies have demonstrated the importance of using finer horizontal resolution for simulating ozone production. Coarser horizontal resolution causes unrealistic dilution of transported pollution plumes of VOCs and nitrogen oxides (NO_x_) as well as smoothing local topography, which artificially impacts the transport of air pollutants (Monks et al., 2015). This dilution of NO_x_ in particular can cause ozone production to be inaccurately simulated due to nonlinearities in the process (Cohan et al., 2006). Several studies have demonstrated that using ∼12 km horizontal resolution is generally sufficient to represent ozone formation and loss processes (Cohan et al., 2006), NO_2_ (Valin et al., 2011; Yamaji et al., 2014), and mortality from population‐weighted MDA8 (maximum daily 8‐hr average) ozone (Thompson & Selin, 2012) at the regional scale. Other studies have emphasized the need for even finer horizontal resolution for modeling local events such as shipping (D. D. Davis et al., 2001; Vinken et al., 2011), power plants (Valin et al., 2011), urban/industrial regions (Colette et al., 2014; Gan et al., 2016; Liang & Jacobson, 2000; Yamaji et al., 2014) especially at night (Zakoura & Pandis, 2018), and complex terrain/coastal regions (Gan et al., 2016). Here, CESM/CAM‐chem‐SE is used to explore the importance of finer horizontal resolution on accurately simulating ozone and ozone precursors at the regional scale. Simulations are performed at a typical global horizontal resolution of ∼111 km (∼1°) and also with mesh refinement down to ∼14 km (∼1/8°) over CONUS.

CESM/CAM‐chem also has the capability to use different chemical mechanisms of varying chemical complexity. The default MOZART‐TS1 chemical mechanism has full tropospheric and stratospheric chemistry (Emmons et al., 2020) and includes a volatility basis set (VBS) scheme for secondary organic aerosol (SOA) formation (Tilmes et al., 2019). More complex and updated gas‐phase chemistry for isoprene and terpenes (biogenic VOCs) was recently added into MOZART‐TS1 to create the MOZART‐TS2 chemical mechanism (Schwantes et al., 2020). In CESM/CAM‐chem, using MOZART‐TS2 compared to MOZART‐TS1 reduces the model overprediction of MDA8 surface ozone as compared to the US EPA Clean Air Status and Trends Network monitoring data particularly over the Southeast US (Schwantes et al., 2020) where emissions of biogenic VOCs are most important. However, a large MDA8 surface ozone bias remains, which is potentially due to the coarse horizontal resolution of 0.9° × 1.25° used by Schwantes et al. (2020). This work expands on this previous work to explore whether more complex chemistry has a greater impact on simulated ozone at finer horizontal resolutions, or in other words whether more complex chemistry is needed to achieve the full benefit of using finer horizontal resolution.

In this work, CESM/CAM‐chem‐SE or MUSICAv0 is described in Section 2.1. Model simulations varying horizontal resolution and chemical complexity are performed (Section 2.2) to demonstrate the capabilities of CESM/CAM‐chem‐SE and better understand the impact of horizontal resolution and chemical complexity on ozone and ozone precursors over CONUS (Section 3.1). The model results are compared to observations collected during five aircraft campaigns across the US in 2013 described in Section 2.3. By comparing the model results to ozone, ozone precursors, meteorological variables, and VOC oxidation products measured in these aircraft campaigns, model skill at representing ozone and the physical and chemical processes that determine the ozone distribution is evaluated (Section 3.2). By more accurately simulating ozone and the physical and chemical processes responsible for ozone formation and loss, this work increases the accuracy and confidence in the model's predictive capability, which is important for forecasting, source apportionment, and other model applications. Finally, the physical and chemical processes that are missing or erroneous in the model are highlighted (Section 4) to help prioritize future work (Section 5).

## 2 Methods

The model configuration is described in Section 2.1, the simulations performed are summarized in Section 2.2, and the five aircraft campaigns used to evaluate the model are described in Section 2.3.

### 2.1 Model Description

Numerous past studies (Baer et al., 2006; Dennis et al., 2012; Fournier et al., 2004; Lauritzen et al., 2018; Zarzycki, Levy, et al., 2014; Zarzycki, Jablonowski, & Taylor, 2014) have incrementally developed a configuration of CESM/CAM that uses the SE dynamical core to support both uniform horizontal resolution grids and grids with the capability of mesh refinement (i.e., local increases in horizontal resolution). CAM‐SE with mesh refinement has led to improved representation of tropical cyclones (Zarzycki, Jablonowski, & Taylor, 2014), orographic precipitation (Rhoades et al., 2016), and better understanding of how finer horizontal resolution impacts climate simulations (Zarzycki, Levy, et al., 2014). This work expands on these past studies to add full tropospheric and stratospheric chemistry of gases and aerosols into CESM/CAM‐SE to create CESM/CAM‐chem‐SE or MUSICAv0. The coupling of chemical constituents with the model physics has not fundamentally changed since Lamarque et al. (2012) and Tilmes et al. (2015). As described previously by Lamarque et al. (2012), the model top is ∼40 km. Ozone in the lower stratosphere is controlled by the chemical mechanism, which was last updated for the stratosphere by Gettelman et al. (2019). A layer of ozone and oxygen is included above the model top to ensure accurate calculation of photolysis rates throughout the model (Lamarque et al., 2012). CESM2.2 beta code is used in this work and the model advancements have been released for community use in CESM2.2 (https://wiki.ucar.edu/display/MUSICA/MUSICA+Home).

Two SE grids described previously by Lauritzen et al. (2018) are used: the uniform ne30 grid and the ne0conus30 × 8 grid with mesh refinement over CONUS. In ne30, the grid cells are nominally ∼111 km (∼1°) across, so hereafter this is referred to as ∼111 km horizontal resolution. In ne0conus30 × 8, the grid cells are nominally ∼14 km (∼1°/8°) across over CONUS and ∼111 km across over the rest of the globe, so hereafter this is referred to as ∼14 km horizontal resolution. In all figures, the value is plotted based on the center latitude longitude of each grid cell and not the native area. The ∼111 and ∼14 km horizontal resolution simulations use a physical/chemical time step of 30 and 3.75 min, respectively. Using the ne0conus30 × 8 grid increases the simulation cost by approximately a factor of 29 compared to the ne30 grid due to the increased number of grid cells and the decreased physical/chemical time step needed to satisfy the Courant‐Friedrichs‐Lewy criterion.

A major step in developing CESM/CAM‐chem‐SE with mesh refinement is implementing input and emission data sets appropriate for finer grid sizes. Biogenic emissions are calculated online using the Model of Emissions of Gases and Aerosols from Nature (MEGAN) v2.1 (Guenther et al., 2012) in the community land model (CLM) as further described in Lamarque et al. (2012). The CLM model uses the same horizontal grid resolution as CAM‐chem. The plant functional type (PFT) and leaf area index input files to CLM are regridded to the two grids described above. The list of biogenic VOCs is extended to include all VOCs available in MEGAN for both chemical mechanisms as done in Schwantes et al. (2020). Because the biogenic emissions are calculated online in the CLM model, differences in meteorological variables such as temperature between the simulations will directly impact the emission rates of the biogenic VOCs.

The remaining emissions are generated offline. Daily fire emissions are from the Fire INventory from NCAR v1.5 (Wiedinmyer et al., 2011), which are provided as point sources and are conservatively mapped to the SE grids using a new tool available for download here: https://wiki.ucar.edu/display/MUSICA/Grid+FINN. Anthropogenic emissions from the CAMS version 3.1 global inventory with a native horizontal resolution of 0.1° × 0.1° (Granier et al., 2018) are regridded conservatively to the SE grids. The CAMS emissions are provided on a monthly time scale at the surface. MUSICA starts with the CAMS emissions because with these emissions users can create grids with regional refinement anywhere in the world. Adding the capability of replacing the CAMS emissions with regional emission inventories such as the US EPA National Emissions Inventory (NEI) that includes enhanced regional knowledge, a diel cycle, and vertical information is an important goal for future work. When these fire and anthropogenic emission inventories are regridded, emissions from fine scale features are better resolved and less artificially diluted (i.e., plumes are averaged with fewer background values) when using the ne0conus30 × 8 grid with ∼14 km horizontal resolution over CONUS compared with using the ne30 grid with ∼111 km horizontal resolution uniformly. Ocean and soil emissions are from the POET inventory (Granier et al., 2005). Aircraft emissions are from the Community Emissions Data System (Hoesly et al., 2018). Volcanic emissions are from the GEIA inventory (Andres & Kasgnoc, 1998) and based on the Volcanic Emissions for Earth System Models data set, version 3.11 (Neely & Schmidt, 2016). Tools for regridding emission inventories to SE uniform and mesh refinement grids are available for download here: https://wiki.ucar.edu/display/MUSICA/Regridding+emissions.

Temperature and horizontal winds are nudged to GEOS5 meteorology from the NASA Global Modeling and Assimilation Office. The GEOS5 data are available at 0.67° × 0.5° horizontal resolution every 6 hr for 2010–2012 and at 0.31° × 0.25° every 3 hr for 2013. The GEOS5 meteorological fields (56 vertical levels) are interpolated vertically and horizontally to each CAM grid mesh (32 vertical levels) and a correction for differences in the topography between GEOS5 and CAM is applied. A new specified dynamics scheme, which is fully described in the CAM6 user guide (NCAR, 2017), is used in this work. The new scheme is more flexible, contains more features, and works with the SE dynamical core compared to past specified dynamics schemes used in CAM‐chem (Emmons et al., 2020; Lamarque et al., 2012; Schwantes et al., 2020). Nudging occurred at each time step using the next target nudging force option and the weak time scale option.

Separate approaches are used to generate the initial conditions for CLM and CAM‐chem. To generate CLM initial conditions for the ne0conus30 × 8 grid, a CAM simulation is performed using the ne0conus30 × 8 grid for 3 years (January 2010‐December 2012). The CAM simulation uses a highly reduced chemical mechanism compared to that in CAM‐chem, which reduces the cost of the long simulation needed to spin‐up the land model. To generate CAM‐chem initial conditions for the ne0conus30 × 8 grid, for each chemical mechanism a CAM‐chem simulation is performed with the ne30 grid for 2 years, the ne30 file is regridded to the ne0conus30 × 8 grid, and an additional 1 month spin‐up is performed using CAM‐chem with the ne0conus30 × 8 grid.

### 2.2 Description of Simulations

Four main model simulations (Table 1, #1–4) are performed for the entire year of 2013 to evaluate the new configuration of CESM/CAM‐chem‐SE and better understand the impact of chemical complexity and horizontal resolution on ozone and ozone precursors. Two horizontal resolutions are tested: a global uniform resolution of ∼111 and ∼111 km horizontal resolution globally and mesh refinement over CONUS down to ∼14 km. Two chemical mechanisms of varying complexity are also evaluated: MOZART‐TS1 and MOZART‐TS2.1. MOZART‐TS1, or TS1 hereafter, is the default chemical mechanism used within CESM2/CAM‐chem (Emmons et al., 2020) and includes full tropospheric and stratospheric chemistry. The VBS SOA scheme in TS1 (Tilmes et al., 2019) uses SOA yields from the low‐NO_x_ pathway for all SOA formation under the assumption that SOA forms dominantly from the low‐NO_x_ pathway over most of the Earth. MOZART‐TS2.1, or TS2.1 hereafter, includes more complex isoprene and terpene gas‐phase chemistry as developed by Schwantes et al. (2020) (MOZART‐TS2) and a more complex VBS SOA scheme developed by Hodzic et al. (2016) and implemented into CESM/CAM‐chem by Jo et al. (2021). The SOA scheme in TS2.1 includes SOA yields from both the low‐ and high‐NO_x_ pathways for anthropogenic and biogenic VOCs. Because SOA yields under low‐NO_x_ conditions are generally higher than under high‐NO_x_ conditions, the impact of switching from TS1 to TS2.1 is to reduce SOA formation particularly in regions with high NO_x_ concentrations. New species and reactions added to TS2 to create TS2.1 are listed in Tables S1 and S2 in Supporting Information S1 of the supplement. TS2.1 contains 46 more transported species and 50 more non‐transported species (i.e., radicals) compared to TS1. Using the TS2.1 mechanism increases the simulation cost by around a factor of 1.6 in CESM2.2/CAM‐chem‐SE compared with the TS1 mechanism. Cost estimates will depend on many factors including the computational system used and the amount of output saved, for the latest cost estimates of different configurations, refer to https://wiki.ucar.edu/display/MUSICA/MUSICA+version+0.

**Table 1:** List of Simulations

#	Mechanism^a^	Grid name^b^	US res. (km)^c^	SD time (hr)^d^	Time period^e^
Chemistry and horizontal resolution evaluation					
1	TS1	ne30	∼111	50	January‐December
2	TS1	ne0conus30 × 8	∼14	50	January‐December
3	TS2.1	ne30	∼111	50	January‐December
4	TS2.1	ne0conus30 × 8	∼14	50	January‐December
Specified dynamics sensitivity tests					
5	TS2.1	ne30	∼111	6	August‐September
6	TS2.1	ne30	∼111	12	August‐September
7	TS2.1	ne30	∼111	no CONUS^f^	August‐September
8	TS2.1	ne0conus30 × 8	∼14	6	August‐September
9	TS2.1	ne0conus30 × 8	∼14	12	August‐September
10	TS2.1	ne0conus30 × 8	∼14	no CONUS^f^	August‐September

^a^ TS1 is the MOZART‐TS1 mechanism (Emmons et al., 2020) and TS2.1 is the MOZART‐TS2.1 mechanism (Hodzic et al., 2016; Jo et al., 2021; Schwantes et al., 2020).

^b^ ne30 = ∼111 km globally uniform resolution and ne0conus30 × 8 = ∼111 km global resolution with mesh refinement down to ∼14 km over the conterminous US (CONUS).

^c^ Average horizontal resolution over CONUS for the selected grid.

^d^ Specified dynamics (SD) relaxation time.

^e^ Months simulated in year 2013.

^f^ No nudging over CONUS, but a 50‐hr relaxation time everywhere else.

These four chemistry and horizontal resolution evaluation tests are nudged lightly with a 50‐hr relaxation time throughout the entire model domain (i.e., globally and for all vertical levels) to GEOS5 meteorological data. To evaluate the impact of different specified dynamics options on the results, six sensitivity tests (Table 1, #5–10) are performed for August‐September 2013. For each of the two horizontal resolutions using TS2.1 chemistry, three different specified dynamics options are tested: 6‐hr relaxation time over the entire domain, 12‐hr relaxation time over the entire domain, and no nudging over CONUS, but a 50‐hr relaxation time everywhere else.

### 2.3 Description of Observations

The flight tracks for the five aircraft campaigns conducted in 2013 used to evaluate CESM/CAM‐chem‐SE are shown in Figure S1 in Supporting Information S1. Two aircraft campaigns focused more on urban regions: DISCOVER‐AQ‐TX (Deriving Information on Surface conditions from Column and Vertically Resolved Observations Relevant to Air Quality) over Houston, Texas, in September and DISCOVER‐AQ‐CA over California in January‐February (Crawford & Pickering, 2014). The other three aircraft campaigns focused mostly on the Southeast US and include the SENEX (Southeast Nexus) campaign in June‐July (Warneke et al., 2016); the NOMADSS (Nitrogen, Oxidants, Mercury and Aerosol Distributions, Sources, and Sinks) campaign in June‐July (Carlton et al., 2018); and the SEAC^4^RS (Studies of Emissions, Atmospheric Composition, Clouds, and Climate Coupling by Regional Surveys) campaign in August‐September (Toon et al., 2016). Tables S3 and S4 in Supporting Information S1 describe the observations used throughout this work including the instrumentation, uncertainties, and contributors. As shown with the blue boxes in Figure S1 in Supporting Information S1, the analysis is restricted to include only data from the Southeast US (94.5°–75°W and 29.5°–40°N) for three of these aircraft campaigns: SENEX, NOMADSS, and SEAC^4^RS, only data from Los Angeles and Central Valley region of California (122.2°–117.5°W and 34.5°–39.5°N) for DISCOVER‐AQ‐CA, and only data from Houston, Texas (97°–94.5°W and 28.5°–31.5°N) for DISCOVER‐AQ‐TX. The 1‐min merges for SEAC^4^RS Revision 7, SENEX Revision D, NOMADSS Revision 5, DISCOVER‐AQ‐CA Revision 4, and DISCOVER‐AQ‐TX Revision 3 are used. These field campaigns mostly occur in the summer or in locations with high temperatures throughout the year like Southern California. This work and these past field campaigns focus on the summer period when surface ozone itself and photochemical production of ozone is often highest over CONUS (Fleming et al., 2018; Monks et al., 2015) and when surface ozone model biases are greatest in CESM/CAM‐chem (Emmons et al., 2020; Tilmes et al., 2015).

The model output is saved along the aircraft flight tracks corresponding to the observational times at runtime such that any improvement due to the reduction in the chemical timestep when using finer horizontal resolution is included in the analysis. Vertical interpolation is applied. In the horizontal direction, the two grid cells closest in distance to the observational point based on the grid cell center are averaged with a weight based on the inverse distance. This two grid cell horizontal interpolation produced nearly identical overall results to simply selecting the grid cell whose center is closest in distance to each observational point (Figure S10 in Supporting Information S1). To facilitate further evaluation of this extensive data set, model output of the closest nine grid cells to each observational point for all five aircraft campaigns discussed are available publicly online (see the Model Data Availability description in the Data Availability Statement section). The closest nine grid cells were saved to correspond with the Gauss‐Lobatto‐Legendre grid and cubed‐sphere grid system used by the SE dynamical core (Lauritzen et al., 2018) and if needed allows future work to expand on this analysis to evaluate the spatial representativeness of the model and aircraft comparisons (e.g., Souri et al., 2022).

## 3 Results

First, in Section 3.1 the impact of using more complex chemistry (TS1 and TS2.1) and finer horizontal resolution (∼111 and ∼14 km) on ozone and ozone precursors is evaluated at the surface averaged over August 2013. Because the emissions and input data sets to CESM/CAM‐chem‐SE have been regridded (Section 2.1), biogenic, fire, and anthropogenic emission plumes are less artificially diluted using the ∼14 km horizontal resolution with smaller grid cells compared to that using the ∼111 km horizontal resolution. These differences in emissions between the horizontal resolutions combined with differences in meteorology and chemistry cause large differences in ozone and ozone precursors at the surface especially when simulating fine scale features such as urban and fire plumes. Additionally, using more complex gas and SOA chemistry causes more pronounced differences in simulated surface concentrations of ozone, organic aerosol, formaldehyde, and isoprene oxidation products when using finer horizontal resolution where chemical regimes are better resolved. Second, in Section 3.2 the model results are compared with five aircraft campaigns during 2013 demonstrating that finer horizontal resolution reduces model biases compared to the aircraft observations in ozone precursors such as NO_x_ and CO, but has less of an impact on model biases in ozone itself.

### 3.1 Differences Caused by Changing Resolution and Chemistry

**Figure 1:**
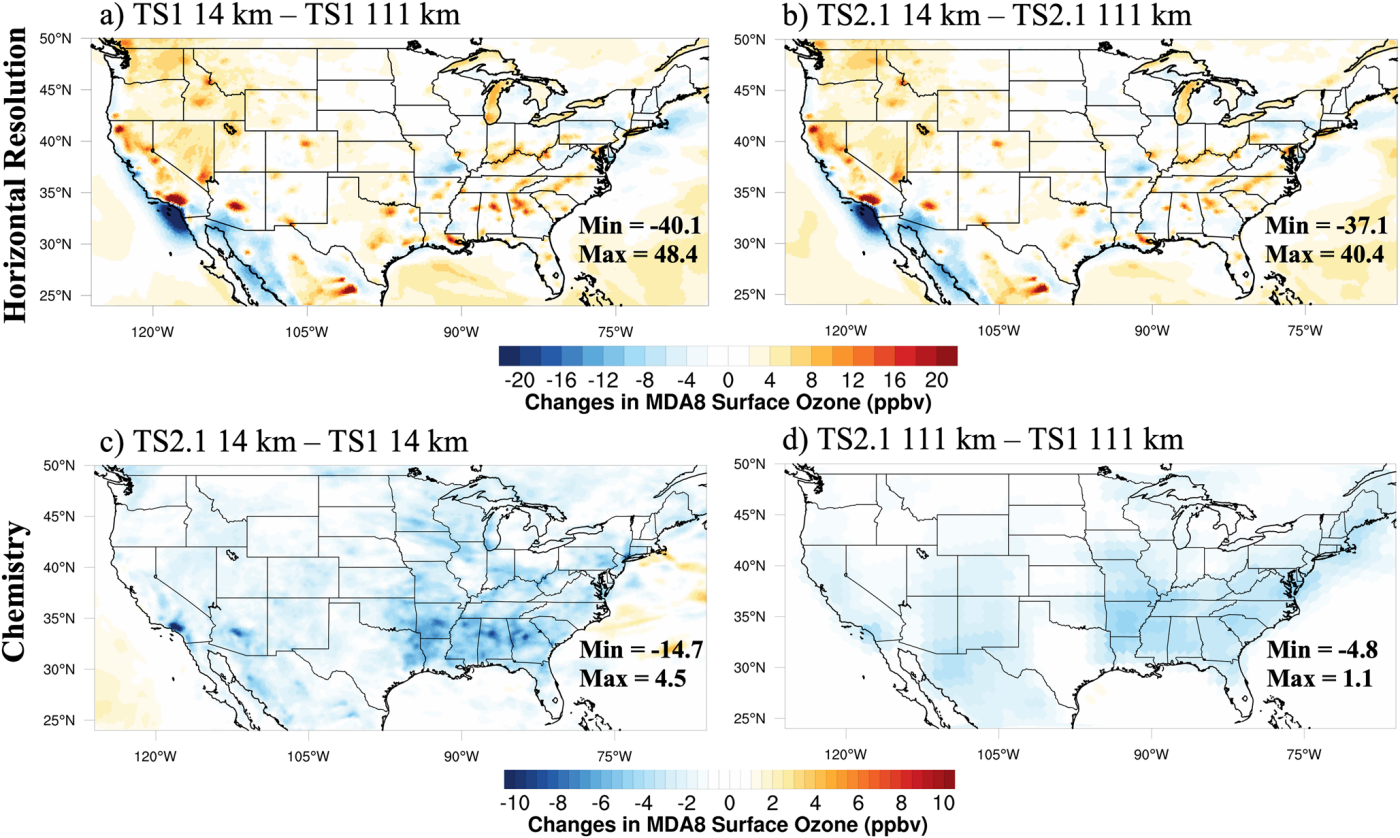
August 2013 average MDA8 surface ozone differences between different horizontal resolutions with chemistry fixed (a and b) and different chemical mechanisms with horizontal resolution fixed (c and d).

**Figure 2:**
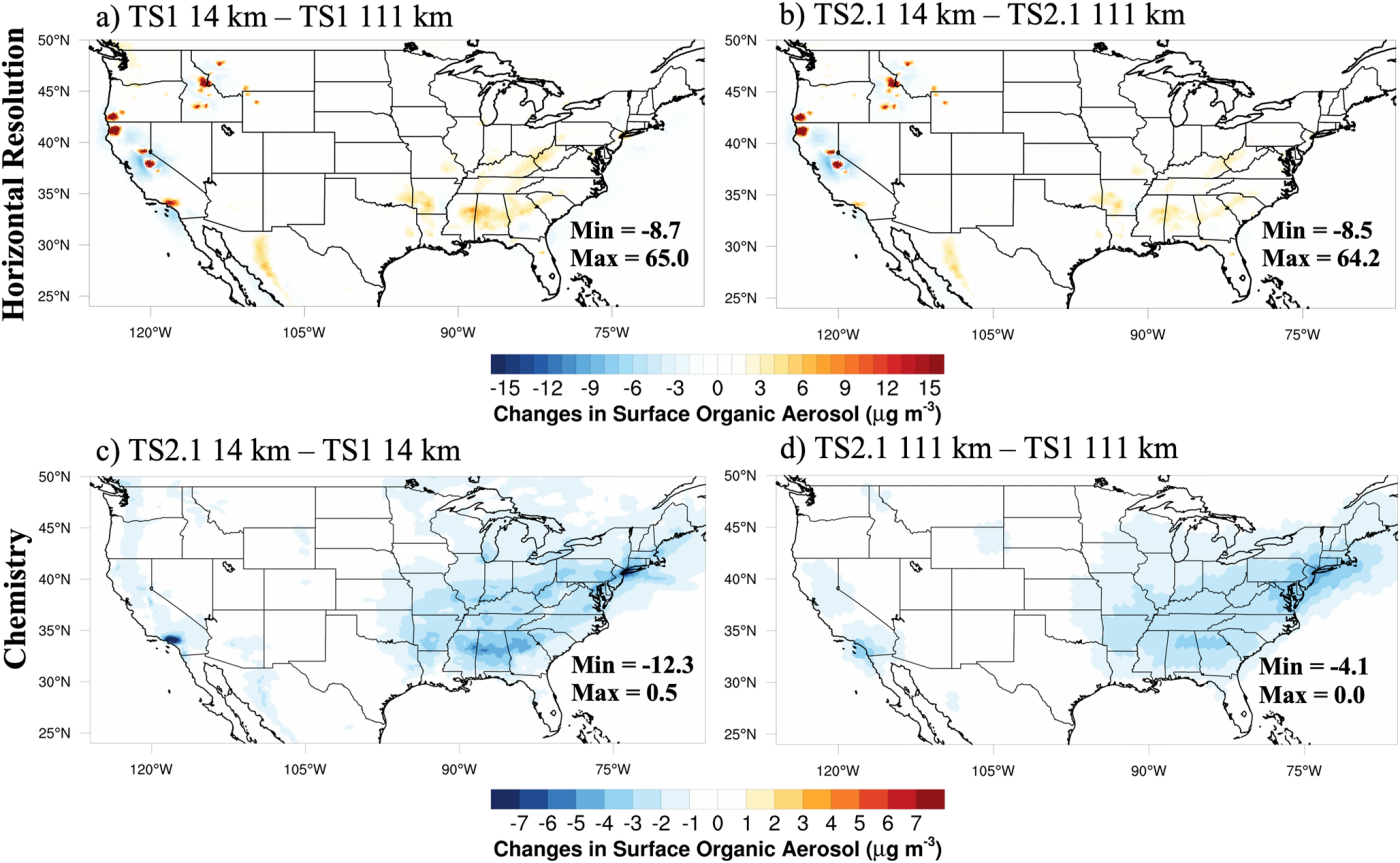
August 2013 average surface organic aerosol differences between different horizontal resolutions with chemistry fixed (a and b) and different chemical mechanisms with horizontal resolution fixed (c and d).

**Figure 3:**
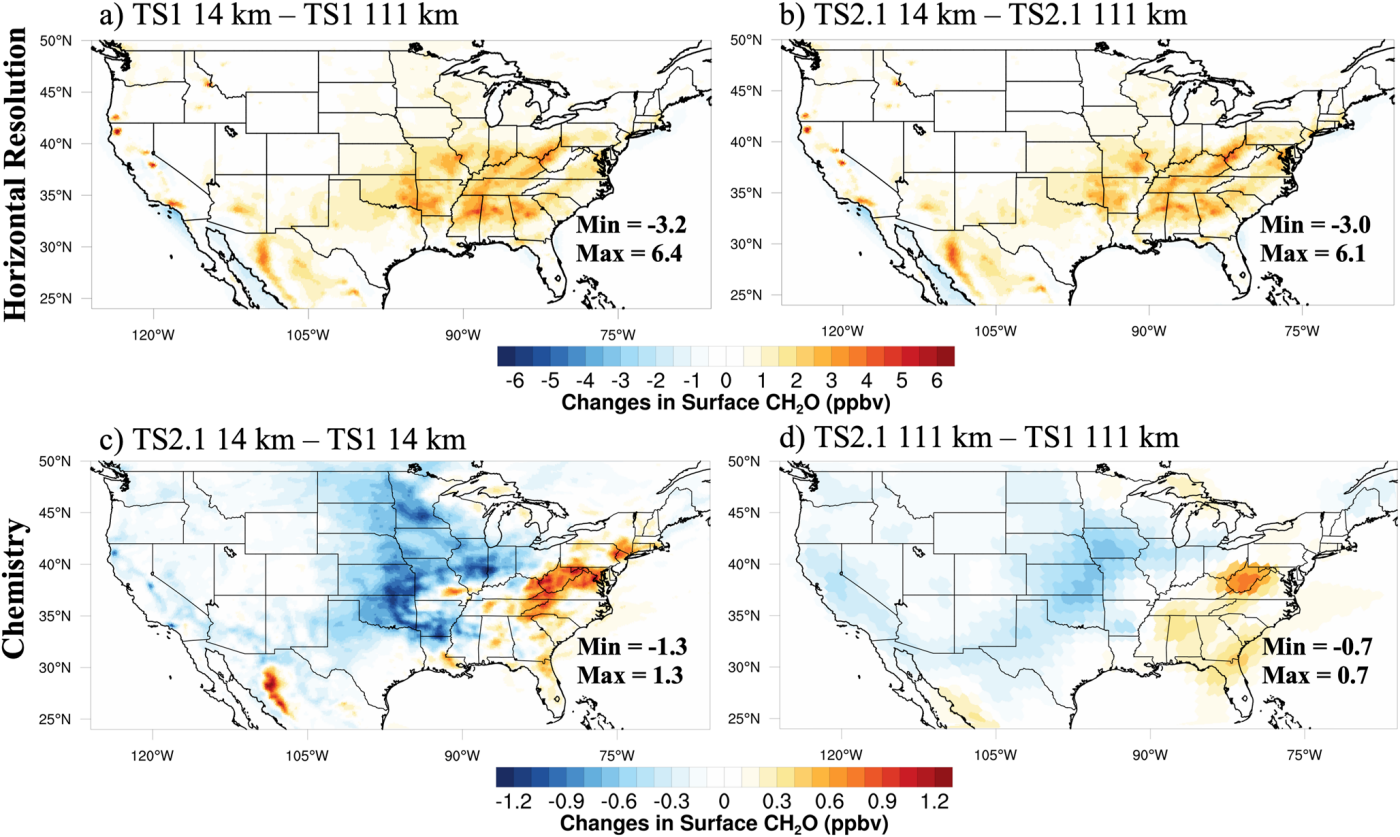
August 2013 average surface formaldehyde (CH_2_O) differences between different horizontal resolutions with chemistry fixed (a and b) and different chemical mechanisms with horizontal resolution fixed (c and d).

Differences due to changes in horizontal resolution and chemistry averaged over August 2013 at the lowest model level, hereafter referred to as surface, are shown for MDA8 ozone, organic aerosol, and formaldehyde (Figures 1, 2, 3). Similar figures for NO_x_, carbon monoxide (CO), hydrogen oxide radicals (HO_x_), isoprene and isoprene oxidation products including isoprene hydroxy hydroperoxide (ISOPOOH), isoprene hydroxy nitrate (ISOPN), methacrolein, and methyl vinyl ketone are presented in the supplement (Figures S2–S9 in Supporting Information S1). When the grids are different, the coarser resolution results are bilinearly interpolated to the finer resolution grid prior to subtraction.

In Figures 1a and 1b, the differences in MDA8 ozone between the horizontal resolutions are largest in and downwind of urban regions where NO_x_ emissions are less artificially diluted at ∼14 km compared to ∼111 km horizontal resolution (Figures S2a and S2b in Supporting Information S1). The concentrated patches of high organic aerosol throughout the Northwest US in Figures 2a and 2b display the location of large wildfires during August 2013. These wildfires are clearly better represented using the ∼14 km resolution than at the ∼111 km resolution for organic aerosol, but also to some degree for ozone (Figures 1a and 1b) and formaldehyde (Figures 3a and 3b). Differences in the area‐weighted averages at the surface over the same regions defined in the field campaign analysis (Figure S1 in Supporting Information S1) are listed in Table S5 in Supporting Information S1. On average, differences between the ∼111 and ∼14 km resolutions for MDA8 ozone and many other compounds are modest.

As shown in Table 2, the tropospheric ozone burden over CONUS is slightly higher at the ∼14 km compared to the ∼111 km horizontal resolution likely caused by the higher total lightning NO emissions at ∼14 km over CONUS. The lightning NO emissions parameterization in CESM/CAM‐chem is described by Lamarque et al. (2012). This work focuses on the surface and lower troposphere over CONUS, where generally lightning NO is not as important (Allen et al., 2012; Kaynak et al., 2008), except over rural regions with low anthropogenic NO_x_ emissions (Kang et al., 2020). Future work will investigate improving the representation of lightning emissions in variable resolution models. As shown by ozone and nitric acid (HNO_3_), dry and wet deposition over CONUS at least on average are similar between the grids with ozone dry deposition and nitric acid wet deposition being slightly higher at the ∼14 km versus ∼111 km horizontal resolution (Table 2).

**Table 2:** Tropospheric^a^ Burden, Lifetime, Deposition, and Emissions Calculated Over Only CONUS for the Different Grids Using TS1

Calculation (unit)	∼111 km^b^	∼14 km^c^
Ozone Burden (Tg)	6.1	6.4
CO Burden (Tg)	4.4	4.7
CH_4_ Burden (Tg)	57.4	56.5
CH_4_ Lifetime (yr)	6.3	7.2
Ozone Dry Dep (Tg/yr)	43.9	47.3
HNO_3_ Dry Dep (Tg/yr)	4.7	4.7
HNO_3_ Wet Dep (Tg/yr)	5.2	5.9
Lightning NO Emis (Tg N/yr)	0.2	0.4
Isoprene Surface Emis (Tg/yr)	13.5	25.0

*Note.* Dep = deposition; Emis = emissions; CO = carbon monoxide; CH_4_ = methane; HNO_3_ = nitric acid.

^a^ Troposphere defined as region where ozone is less than or equal to 150 ppb.

^b^ ∼111 km horizontal resolution globally (ne30 grid).

^c^ ∼14 km over CONUS and ∼111 km horizontal resolution over the rest of the globe (ne0conus30 × 8 grid).

Generally, MDA8 surface ozone decreases across the Eastern US when using TS2.1 compared with TS1 at ∼111 km horizontal resolution (Figure 1d). However, at ∼14 km horizontal resolution, using TS2.1 versus TS1 reduces MDA8 ozone to an even greater degree up to ∼15 ppbv (Figure 1c). This suggests that using more complex chemistry may be more important at finer horizontal resolutions where a greater range of chemical regimes are resolved. In the Southeast US, on average MDA8 ozone is reduced more with updates to chemistry (−2.9 ppbv at ∼111 km and −3.2 ppbv at ∼14 km) than updates to horizontal resolution (0.9 ppbv with TS1 and 0.6 ppbv with TS2.1) as shown in Table S5 in Supporting Information S1. These results demonstrate that updating and adding more complexity in model processes such as chemistry can have a large impact on regional ozone.

Likely the differences in simulated ozone between TS2.1 and TS1 especially at finer horizontal resolution are caused by TS2.1 including a more complex representation of organic nitrate formation and fate than TS1 (Schwantes et al., 2020). Additionally TS2.1 does not fix the second‐generation peroxy radical (RO_2_) fate to that of the first‐generation (Schwantes et al., 2020). For example, organic nitrates which form from the RO_2_ + NO pathway in the first‐generation step react with the hydroxyl radical, OH, in TS2.1 to form peroxy radicals that isomerize or react with NO or HO_2_. Most reduced chemical mechanisms like TS1 assume that the first‐generation organic nitrates react with OH to continue to form products from the RO_2_ + NO channel in the second‐generation step, contrary to observations from field campaigns, which have detected products from mixed peroxy radical fates such as isoprene dihydroxy hydroperoxy nitrates (B. H. Lee et al., 2016; Xiong et al., 2016).

The TS2.1 mechanism also includes updates to the SOA VBS scheme to include SOA formation from both low‐ and high‐NO_x_ pathways (Section 2.2). The ultimate impact of using TS2.1 compared to TS1 is to decrease SOA in regions with higher NO_x_ concentrations. The effect is enhanced at ∼14 km horizontal resolution where NO_x_ emissions are less artificially diluted (Figures 2c and 2d) than at ∼111 km horizontal resolution. However, even at the ∼111 km horizontal resolution, the organic aerosol is reduced by up to 4.1 μg m^−3^ and on average by 1.9 μg m^−3^ (Table S5 in Supporting Information S1) across the Southeast US. Thus, the more complex SOA scheme is important even at the coarse resolution used in global models. At the finer horizontal resolution, the decrease in organic aerosol (Figure 2c) reduces the loss of gas‐phase NO_x_ reservoir compounds including NO_2_, N_2_O_5_, and organic nitrates to aerosols, which ultimately increases the NO_x_ concentrations in cities (Figure S2c in Supporting Information S1). Due to the non‐linearity in ozone production and the high NO_x_ concentrations in these cities, surface ozone declines, which is particularly apparent in the Los Angeles region in Figure 1c. This heterogeneous chemistry is not included in all reduced mechanisms used in 3D models. CESM does not include the feedback of aerosols on photolysis rates (Lamarque et al., 2012), so this is not contributing to the differences in ozone in Figure 1.

Formaldehyde, representative of the impact of using more complex chemistry and finer horizontal resolution on secondary oxidation products, is shown in Figure 3. Formaldehyde plays an important role for inferring ozone sensitivity (e.g., Schroeder et al., 2017) and is used to estimate isoprene emissions from satellite measurements (Kaiser et al., 2018). Here, formaldehyde is enhanced when using finer horizontal resolution (Figures 3a and 3b). This may be partially due to improved representation of the spatial segregation of NO_x_ and isoprene emissions at ∼14 km resolution (Kaiser et al., 2018) because formaldehyde production from isoprene is NO_x_ sensitive (i.e., higher NO_x_ concentrations produce more formaldehyde). However, because K. Yu et al. (2016) found that formaldehyde generally decreases with finer horizontal resolution in the Southeast US when isoprene emissions are kept constant, the increase in formaldehyde at finer horizontal resolution in Figures 3a and 3b is likely due mostly to the increase in isoprene at ∼14 km resolution (Table 2, Figure S5 and Table S5 in Supporting Information S1), which is discussed more in Section 4.2.1. At the ∼14 km resolution, using TS2.1 produces more pronounced differences compared to TS1 for formaldehyde, CO, HO_x_, and isoprene oxidation products than at the ∼111 km resolution (panels c and d in Figure 3 and Figures S3, S4 and S6–S9 in Supporting Information S1), suggesting that using more complex chemistry is more important at finer horizontal resolutions. Schwantes et al. (2020) uses a box model to describe how NO_x_, HO_x_, ozone, and VOC oxidation products differ between TS1 and TS2 separately for isoprene, *α*‐pinene, *β*‐pinene, limonene, and myrcene, which provide insights into which pathways lead to the changes shown in panels (c and d) in Figures 1 and 3 and Figures S2–S9 in Supporting Information S1.

### 3.2 Comparison With Observations

**Figure 4:**
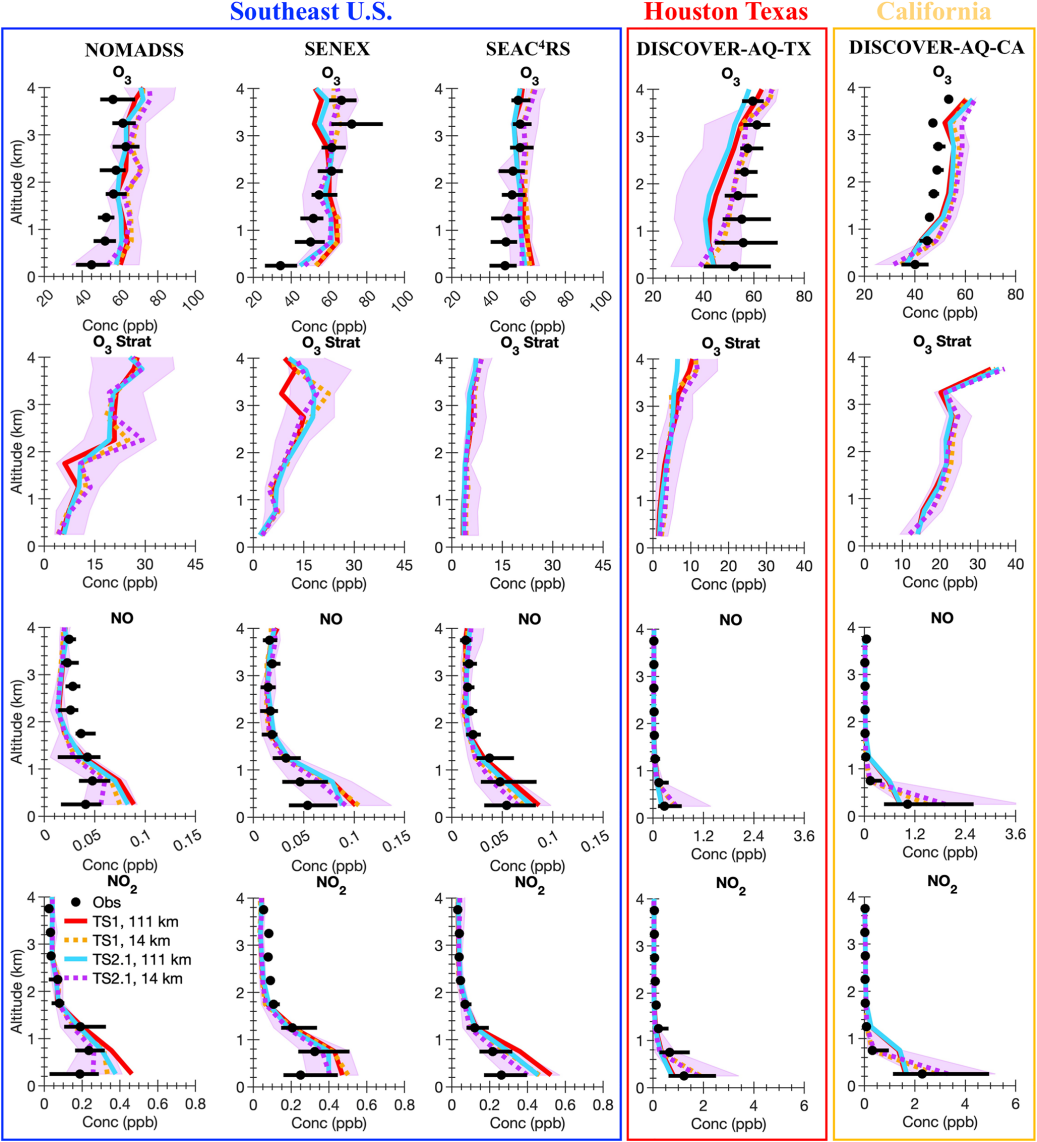
Median vertical profile plots of the five aircraft campaigns for observations (black markers) and the model simulations (colored lines). The black horizontal lines and the purple shading show the 25th and 75th percentiles for the observations and the TS2.1, 14 km model simulation, respectively. Abbreviations are ozone (O_3_), stratospheric ozone tracer (O_3_ Strat), nitrogen oxide (NO), and nitrogen dioxide (NO_2_).

**Figure 5:**
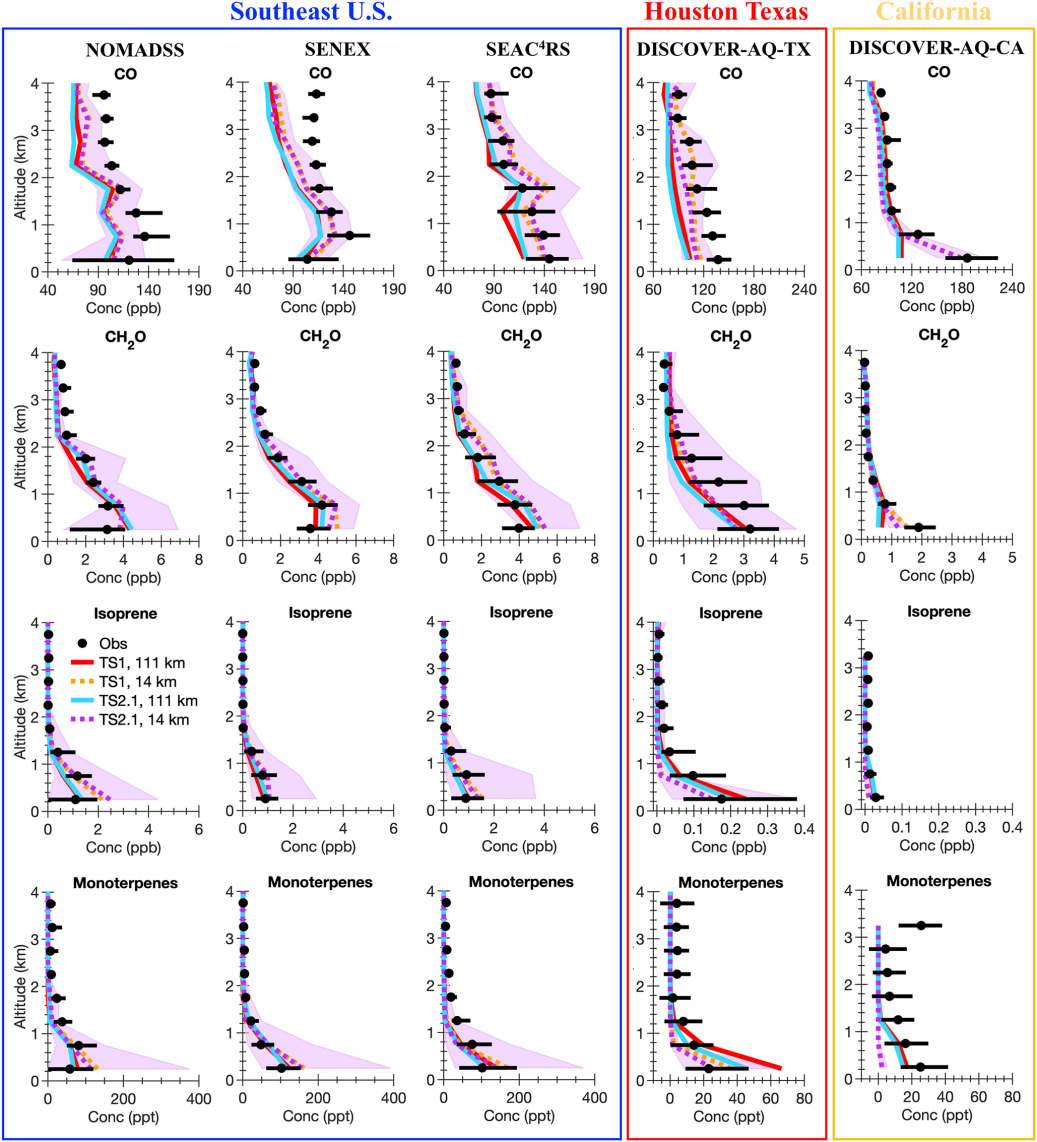
Identical to Figure 4, but median vertical profile plots for carbon monoxide (CO), formaldehyde (CH_2_O), isoprene and monoterpenes.

**Figure 6:**
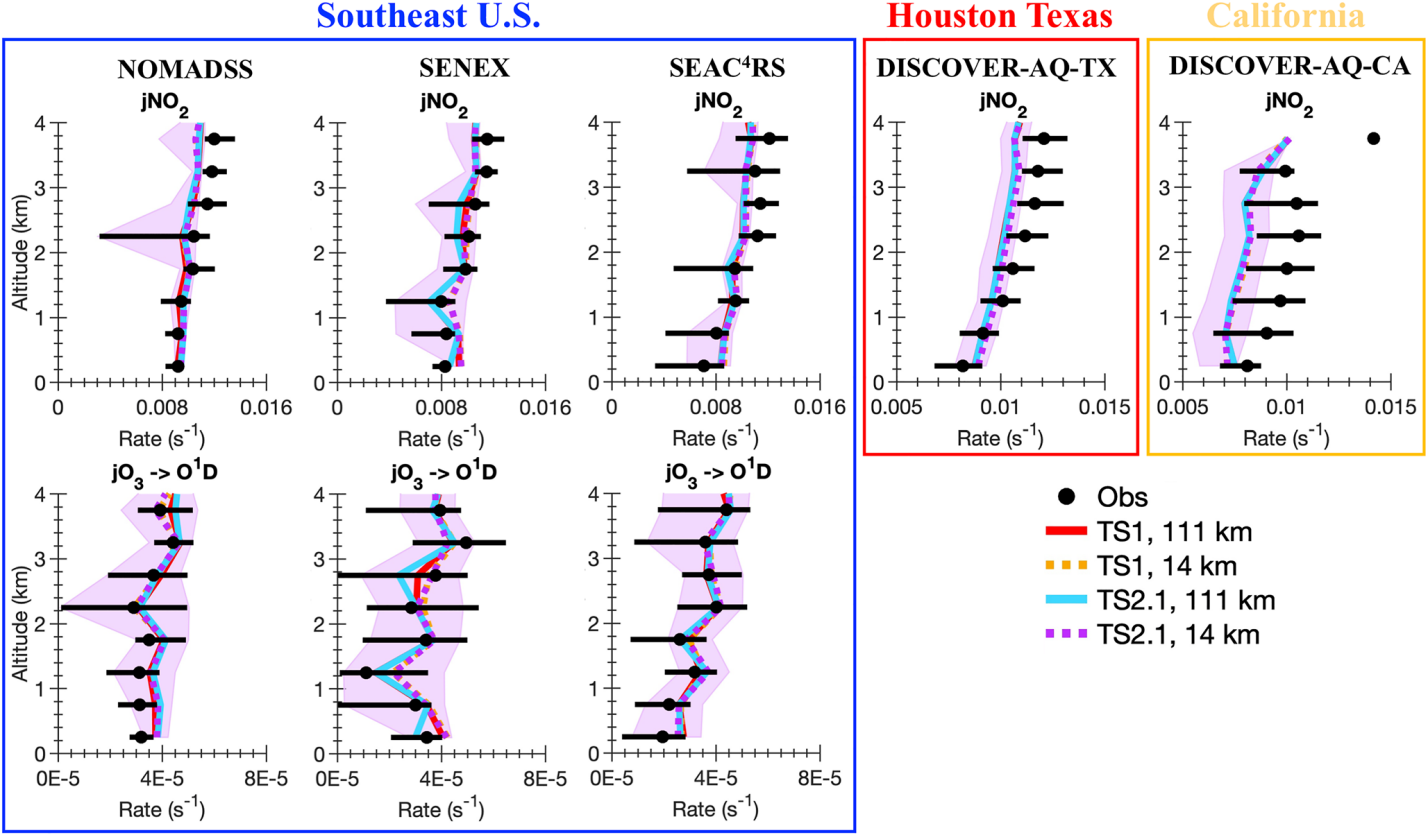
Identical to Figure 4, but for photolysis of NO_2_ (jNO_2_) and when available photolysis of O_3_ (jO_3_).

**Figure 7:**
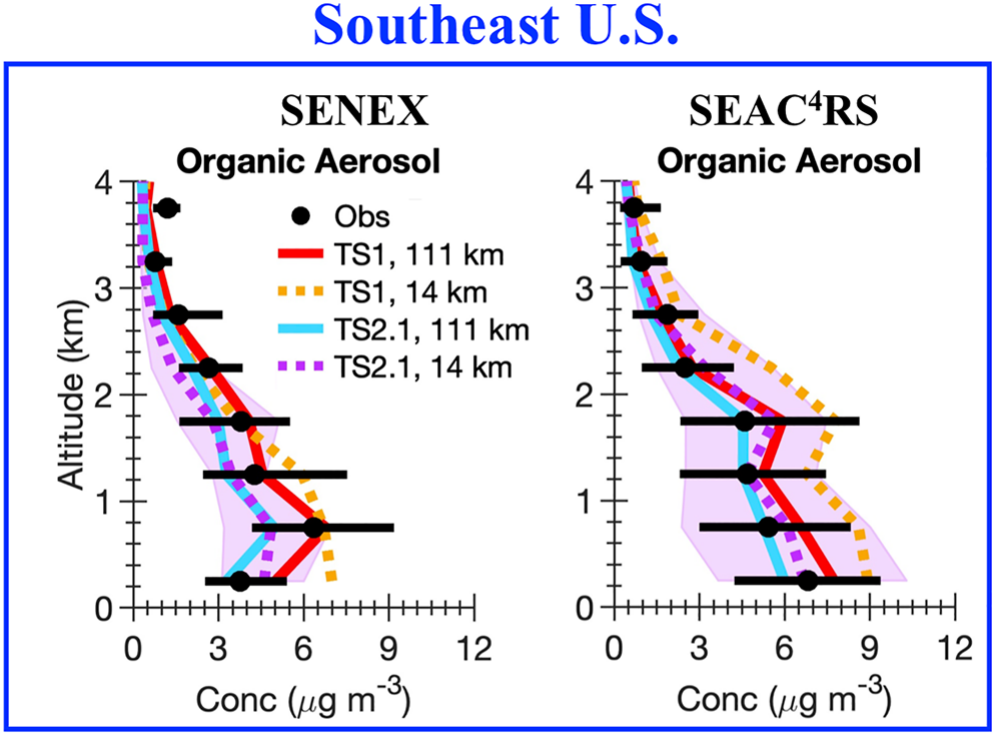
Identical to Figure 4, but for organic aerosol for the two field campaigns that measured organic aerosol by aerosol mass spectrometry.

**Figure 8:**
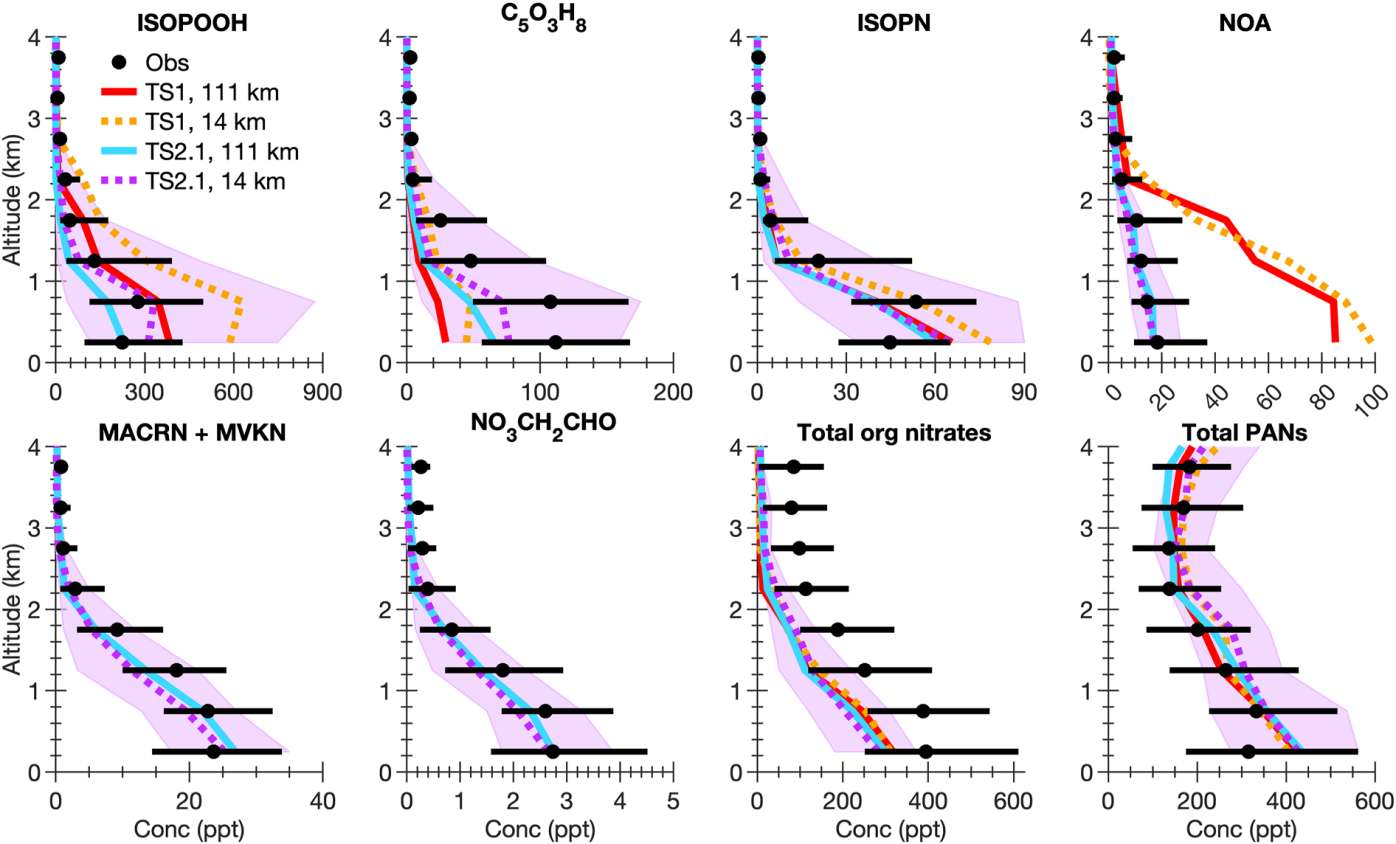
Identical to Figure 4, but for isoprene hydroxy hydroperoxide (ISOPOOH); C_5_O_3_H_8_, which includes all isomers of HPALDs (isoprene hydroperoxy aldehydes), ICHE (isoprene carbonyl hydroxy epoxide), and likely other unknown products; isoprene hydroxy nitrate (ISOPN); propanone nitrate (NOA); methacrolein and methyl vinyl ketone hydroxy nitrates and other isomers of C_4_O_5_H_7_N (MACRN + MVKN); ethanal nitrate (NO_3_CH_2_CHO); total organic nitrates; and total peroxy acyl nitrates (PANs).

A wide variety of observations from the five field campaigns described in Section 2.3 are compared with the model results. Ozone is a complicated pollutant to accurately simulate in models. By evaluating model results against observations not only for ozone, but also ozone precursors (i.e., NO_x_ and VOCs), NO_x_ reservoir compounds, VOC oxidation products, organic aerosol, photolysis rate constants, and temperature, model skill at representing processes important for ozone formation and loss is inferred. For all field campaigns, data influenced by fire plumes (i.e., acetonitrile greater than 0.2 ppbv) are removed from the analysis. We note that if the acetonitrile measurements are intermittent, this method may not completely filter out all fire plumes. For each field campaign, data representative of a single region are selected for analysis as summarized by the blue boxes in Figure S1 in Supporting Information S1. For SEAC^4^RS, SENEX, and NOMADSS data in the Southeast US region only are included. For DISCOVER‐AQ‐TX, data in the region around Houston, Texas, only are included and for DISCOVER‐AQ‐CA, data in the Central Valley and Los Angeles region of California only are included. For field campaigns where limit of detection flags are provided in the merge (SEAC^4^RS and DISCOVER‐AQ), data flagged as lower limit of detection are set to 0 and data flagged as upper limit of detection are removed from the analysis. For field campaigns where limit of detection flags are not provided in the merge (SENEX and NOMADSS), we verified that this did not impact the measurements used in this work. For all field campaigns, data flagged as missing are removed from the analysis. In Figures 4, 5, 6, 7, 8, the median vertical profiles for the observations (black markers) are compared with that for the model simulations (colored lines). The 25th and 75th percentile for the observations (black horizontal line) are compared with the 25th and 75th percentile for the most complex model simulation, TS2.1 chemistry and ∼14 km horizontal resolution (purple shading).

#### 3.2.1 Ozone and Ozone Precursors

For all three campaigns in the Southeast US, more complex chemistry and finer horizontal resolution reduce ozone biases near the surface, but the impact is modest and an ozone bias remains (Figure 4). In two of the campaigns, SENEX and SEAC^4^RS, using more complex chemistry reduces the ozone bias near the surface more than increasing the horizontal resolution. For both SENEX and SEAC^4^RS, the 25th percentile model results are above the median in the observations below 1 km in altitude demonstrating that a large bias in ozone remains.

As diagnosed by the stratospheric ozone tracer (O_3_ Strat) in CAM‐chem, only a small amount of ozone near the surface in the Southeast US is transported down from the stratosphere (Figure 4). The stratospheric ozone tracer concentration near the surface is consistent between the ∼111 and ∼14 km resolutions verifying that at least in the Southeast US the finer horizontal resolution is not leading to an unexpected enhancement of stratospheric ozone near the surface during the summer. The vertical profile of various compounds for the SEAC^4^RS campaign is extended to 12 km in altitude in Figures S11 and S12 in Supporting Information S1. Ozone and the stratospheric ozone tracer are enhanced in the upper troposphere (UT) in the simulations performed at ∼14 km compared to that using ∼111 km (Figure S11 in Supporting Information S1) suggesting that finer horizontal resolution impacts troposphere‐stratosphere exchange and also possibly UT chemistry, which is influenced by convective outflow and lightning‐NO_x_ (Cuchiara et al., 2020).

Although the increased horizontal resolution only moderately impacts ozone itself in the Southeast US, larger changes are observed for ozone precursors such as NO, NO_2_ (Figure 4), CO, formaldehyde (CH_2_O), isoprene, and monoterpenes (Figure 5). The changes in these ozone precursors demonstrate that ozone formation processes are different at finer horizontal resolution. NO and NO_2_ decrease at both horizontal resolutions when increasing the chemical complexity from TS1 to TS2.1 in the Southeast US for all three aircraft campaign comparisons suggesting that NO_x_ biases within models are not only caused by emissions. Differences in chemistry such as NO_x_ recycling and the formation and fate of NO_x_ reservoir compounds can also impact NO_x_ concentrations. Even with improvements to horizontal resolution and chemical complexity, NO and NO_2_ are overpredicted compared to the observations in both the median and 25th and 75th percentiles. This bias in NO_x_ likely contributes to the high bias in ozone in the Southeast US. Past work demonstrates that GEOS‐Chem had similar biases in ozone and NO_x_ when compared to SEAC^4^RS observations and that these biases are reduced by decreasing anthropogenic NO_x_ emissions (Travis et al., 2016). Similarly, the high model bias in NO_y_ compared to observations from SENEX is reduced in WRF‐Chem when using the fuel‐based inventory of motor‐vehicle emissions over the US EPA NEI inventory (McDonald, McKeen, et al., 2018). As discussed more in Section 4.2, updates to replace sections of the global CAMS emission inventory with regional inventories in CESM/CAM‐chem is important for future work.

In all three campaigns in the Southeast US, the median of isoprene and monoterpenes for all simulations is generally near to that of the observations, but the simulations using the ∼14 km horizontal resolution sometimes overpredict near the surface. The 75th percentile of isoprene and monoterpenes is greatly overpredicted in the simulations using ∼14 km horizontal resolution compared to that of the observations (Figure 5). Because the model bias in isoprene and monoterpenes increases when using finer horizontal resolution, this bias is further explored in Section 4. The 75th percentile of CH_2_O in the model is much higher than the observations likely caused by the high bias in the 75th percentile of isoprene.

The other two campaigns, DISCOVER‐AQ‐TX and DISCOVER‐AQ‐CA, focused more on urban regions with greater NO_x_ and less biogenic VOC emissions than that in the Southeast US (Figures 4 and 5). As expected, using more complex isoprene and terpene chemistry generally does not impact ozone or NO_x_ in California and Houston, TX (Figure 4). Clear differences in the ozone vertical profile shape occur when switching from ∼111 to ∼14 km resolution. Ozone is underestimated compared to the observations near the surface especially in Houston, TX.

While finer horizontal resolution only moderately impacts ozone and ozone precursors in the Southeast US, finer horizontal resolution more substantially impacts NO_x_ (Figure 4), CO, and CH_2_O (Figure 5) in Texas and California. In both DISCOVER‐AQ‐TX and DISCOVER‐AQ‐CA, NO_x_ is over‐predicted in the model compared to the observations in the median and sometimes also the 75th percentile using ∼14 km horizontal resolution. In these high NO_x_ urban regions, over‐predicting NO_x_ in the model is likely leading to the underprediction of ozone.

In general, these results demonstrate that ozone precursors and as such the processes forming ozone are better simulated at ∼14 km especially in urban regions even if there are not large differences in the magnitude of ozone itself. Now that we can move seamlessly between ∼14 and ∼111 km horizontal resolutions in CESM, the model processes can be more extensively evaluated and improved, which will hopefully improve model skill at simulating ozone across all scales in the future.

#### 3.2.2 Photolysis

Consistent with previous evaluations of CESM/CAM‐chem against the SEAC^4^RS field campaign (Schwantes et al., 2020), simulated NO_2_ photolysis in all three campaigns in the Southeast US and also in Texas (Figure 6) is overpredicted below 1 km and underpredicted between 2 and 4 km (Figure 6). Using finer horizontal resolution does not improve the model bias in NO_2_ photolysis vertical shape. In California, NO_2_ photolysis is underpredicted in CESM/CAM‐chem at all altitudes below 4 km. The model bias in NO_2_ photolysis may be partially caused by some field campaigns avoiding the sampling of clouds on scales not well‐resolved by the model (Hall et al., 2018). Additionally, the two grid‐cell horizontal interpolation applied here may not fully account for the impact of clouds in neighboring model grid cells on the photolysis rate constant, but the ∼14 km horizontal resolution is likely too coarse for this correction to systematically impact the results. Future work using even finer horizontal resolutions in CESM/CAM‐chem may need to consider applying a correction.

As described by Schwantes et al. (2020), the model bias in the NO_2_ photolysis vertical shape in the Southeast US is likely due to a model bias in clouds. Ryu et al. (2018) using WRF‐Chem demonstrated that incorporating satellite derived clouds improved the NO_2_ photolysis vertical profile and reduced simulated MDA8 surface ozone by 1–5 ppbv. Given the results in Ryu et al. (2018) and that the model bias in the NO_2_ photolysis vertical shape is not improved by using ∼14 km horizontal resolution, evaluating and improving regional cloud biases in CESM/CAM‐chem and better understanding the impact of cloud biases on ozone should be prioritized in the future. Additionally, CESM does not include the feedback of absorbing and scattering aerosols on reducing or enhancing photolysis rates, which impacts oxidants such as HO_x_ and ozone (e.g., Dickerson et al., 1997; Li et al., 2011; Tie et al., 2005). Adding this aerosol‐photolysis feedback into CESM is a priority for future work.

#### 3.2.3 Organic Aerosol

Accurately simulating aerosols is important for simulating ozone for the right reasons in models because aerosols impact photolysis rates (Dickerson et al., 1997) and heterogeneous reactions (Jacob, 2000). To evaluate, model skill at simulating organic aerosol with the updated SOA scheme incorporated into TS2.1 (Section 2.2) the model results are compared to the measured organic aerosol from the aerosol mass spectrometer (AMS) during the SENEX and SEAC^4^RS campaigns. The AMS measures submicron organic aerosol with an upper limit 50% transmission efficiency at an aerodynamic diameter of ∼750 nm as measured during the Atmospheric Tomography Mission (Guo et al., 2021). Consistent with past work (Hodzic et al., 2020; Jo et al., 2021; Tilmes et al., 2019), to compare organic aerosol from CESM/CAM‐chem with the organic aerosol measured by the AMS, the Aitken and accumulation modes in CESM/CAM‐chem are summed for both primary organic aerosol and SOA. Aerosols are modeled in CESM2/CAM‐chem with a four‐mode version of the Modal Aerosol Module (MAM4) (Liu et al., 2016) with updated size bins from Mills et al. (2016). The accumulation mode in CESM generally represents submicron aerosol (Hodzic et al., 2020; Liu et al., 2012; Tilmes et al., 2019) observable by the AMS. A more detailed evaluation of how well the MAM4 scheme simulates the aerosol size distribution in CESM and accounting for possible differences in the aerosol size distribution simulated in CESM and that measured by the AMS is warranted for future work.

Consistent with the differences in surface organic aerosol shown in Figure 2, organic aerosol clearly changes when updating chemistry and using finer horizontal resolution along the SENEX and SEAC^4^RS flight tracks (Figure 7). For SEAC^4^RS, the most complex model configuration (TS2.1 and ∼14 km) represents the organic aerosol observations the best. When using the TS1 mechanism at ∼14 km horizontal resolution, the organic aerosol is biased high, but by applying the updated SOA mechanism in TS2.1 the organic aerosol decreases in line with the observations (Figure 7). In SENEX, the updated TS2.1 chemistry generally underpredicts the organic aerosol compared to the observations and performs worse than the TS1 chemistry. The SENEX campaign occurred earlier in the summer (June‐July) and had lower concentrations of isoprene and monoterpenes at the 75th percentile (Figure 5) than SEAC^4^RS (August‐September). Additionally, the SENEX campaign focused on urban and urban outflow regions and the SEAC^4^RS campaign focused on a more regional perspective of the Southeast US. To better understand the differences in model biases between SENEX and SEAC^4^RS, the spatial biases in organic aerosol are further explored in Section 4.

#### 3.2.4 Isoprene Oxidation Products

Isoprene oxidation products are well represented in CESM/CAM‐chem when using the ∼14 km horizontal resolution and updated TS2.1 chemistry. Because isoprene itself is overpredicted at the 75th percentile in the model (Figure 5), the first‐generation oxidation products including ISOPOOHs and also to a lesser extent ISOPNs are also overpredicted at the 75th percentile in the model (Figure 8). Likely once the isoprene overprediction is corrected, the model bias in first‐generation isoprene oxidation products will also improve.

The observations of C_5_O_3_H_8_ include all isomers of HPALD (isoprene hydroperoxy aldehydes), ICHE (isoprene carbonyl hydroxy epoxides), and likely other unknown products. These observations are compared with HPALD in TS1 and the sum of four HPALD isomers and ICHE in TS2.1. Even with using the finer horizontal resolution and updated TS2.1 chemistry, the model is underpredicting C_5_O_3_H_8_ products (Figure 8). Given the inability of CESM/CAM‐chem to accurately capture C_5_O_3_H_8_ products and the importance of HPALD for HO_x_ recycling, more laboratory studies are needed to identify any unknown C_5_O_3_H_8_ products and better understand the formation and loss pathways of HPALD and ICHE, which are still quite uncertain.

The total organic nitrates are also underpredicted in all model configurations and the finer horizontal resolution only moderately impacts the bias (Figure 8). TS1 and TS2.1 mechanisms do not track organic nitrates produced from C_1_–C_3_ alkane oxidation. As shown by Fisher et al. (2016), this likely contributes to part of this underprediction because of large oil and gas activities in the US and the long atmospheric lifetime of the C_1_–C_3_ alkane organic nitrates. Additionally, CESM/CAM‐chem does not currently track particulate organic nitrates. Adding this is an important priority for future work to better constrain organic nitrate uptake to aerosol and evaluate the total organic nitrate and NO_y_ budget. Additionally, Kenagy et al. (2020) suggest that organic nitrate production from NO_3_‐initiated VOC oxidation at night is comparable to that from OH‐initiated VOC oxidation during the day in the Southeast US. Further evaluation of how well CESM/CAM‐chem represents the boundary layer and chemistry at night may also be needed to fully understand this organic nitrate model bias.

## 4 Discussion

**Figure 9:**
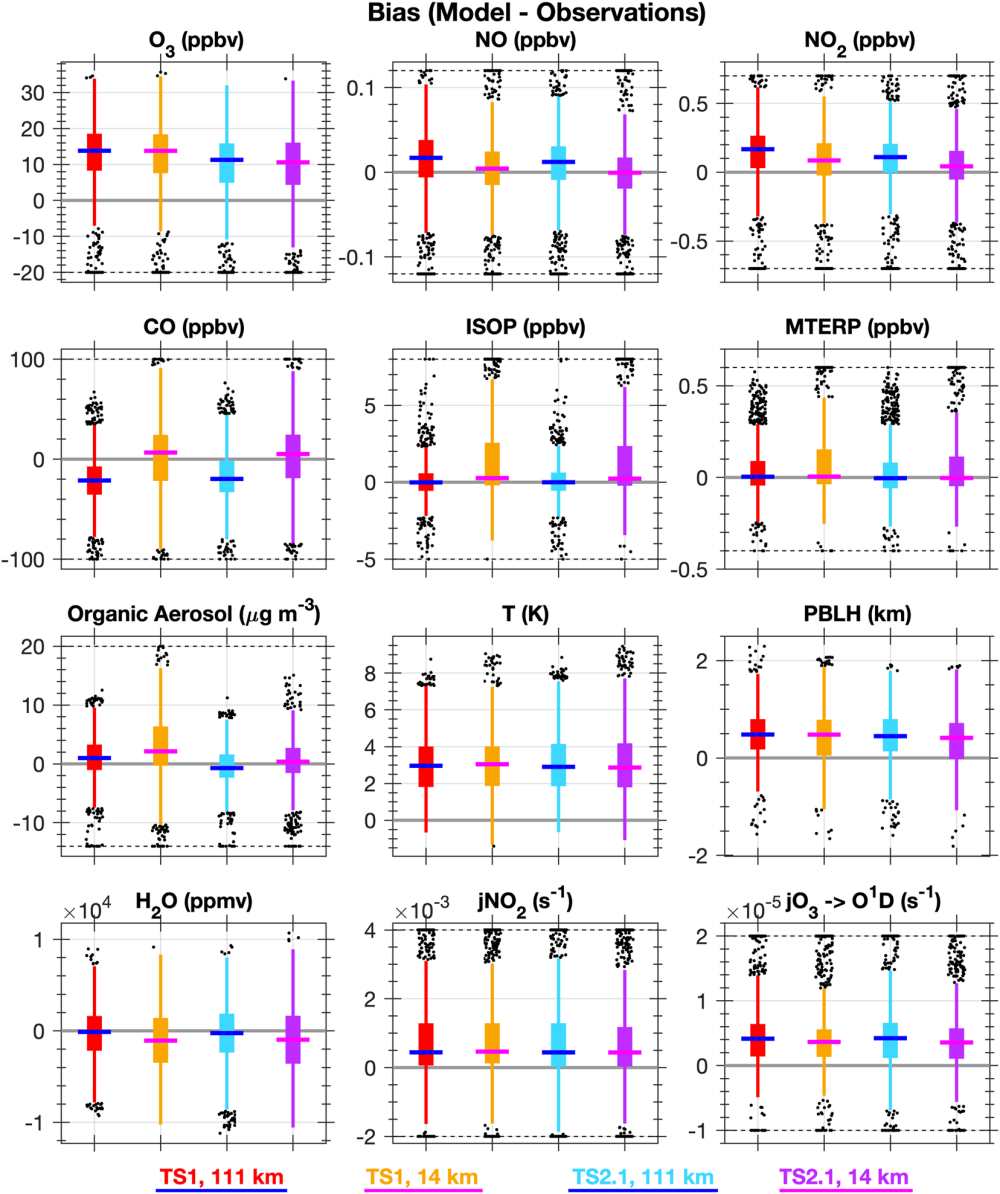
Box and whisker plots for bias (model‐observations) along the SEAC^4^RS flight tracks below 1 km pressure altitude for TS1 at 111 km resolution (red), TS1 at 14 km resolution (gold), TS2.1 at 111 km resolution (light blue), and TS2.1 at 14 km resolution (purple). To highlight differences between the coarse and refined horizontal resolutions, the medians for each case are shown as a blue line for ∼111 km resolution and a magenta line for ∼14 km resolution. The boxes extend to the 25th (*Q*_1_) and 75th (*Q*_3_) percentiles, the whiskers extend to the extremes, and the outliers are displayed as black markers. Outliers are defined as less than *Q*_1_ − 1.5 × IQR or greater than *Q*_3_ + 1.5 × IQR where IQR is the interquartile range (*Q*_3_–*Q*_1_).

**Figure 10:**
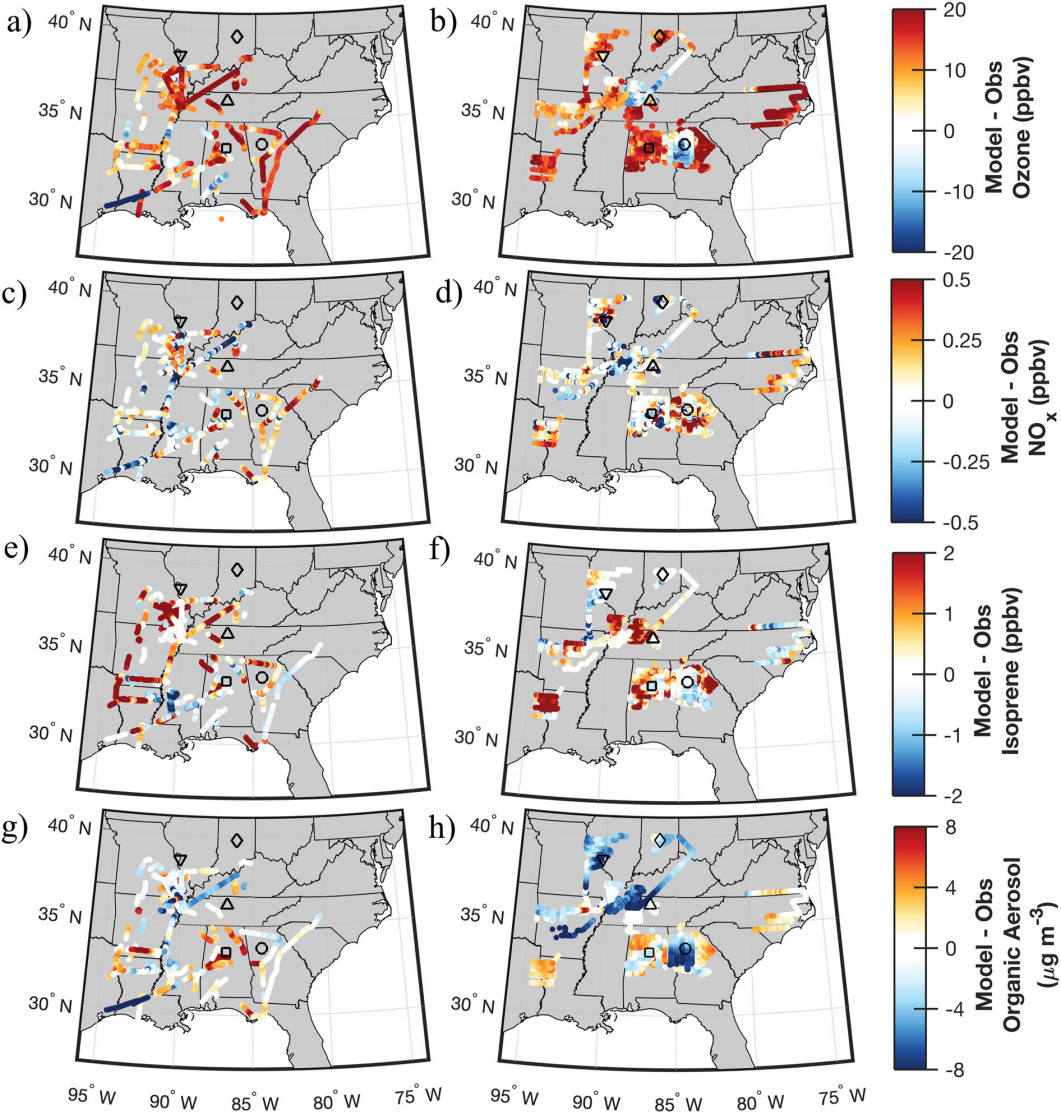
Biases (model [TS2.1, 14 km]—observations) for ozone (a and b), NO_x_ (c and d), isoprene (e and f), and organic aerosol (g and h) for SEAC^4^RS left and Southeast Nexus (SENEX) right flight tracks below 1 km pressure altitude. Markers indicate cities targeted during the SENEX campaign: Atlanta (circle), Birmingham (square), Nashville (upward triangle), St. Louis (downward triangle), and Indianapolis (diamond). Here, only results from the best case model simulation with TS2.1 chemistry and 14 km horizontal resolution are shown.

With the added capability of performing simulations with higher horizontal resolution within CESM/CAM‐chem, confidence that ozone formation and loss processes are accurately represented in the model is increased and the remaining biases in the most complex version of the model (i.e., TS2.1 chemistry at ∼14 km resolution) can be investigated to better understand which processes are missing or erroneous in the model that prevent accurate simulation of ozone. Box and whisker plots for the bias (model‐observations) along the SEAC^4^RS flight tracks below 1 km in altitude are presented in Figure 9 and the spatial distribution of biases along the SEAC^4^RS and SENEX flight tracks below 1 km altitude are shown for the most complex model simulation (TS2.1 chemistry and ∼14 km horizontal resolution) in Figure 10 and for all the model simulations in Figures S16–S23 in Supporting Information S1.

Ozone is overpredicted in all model simulations across much of the Southeast US (Figures 10a and 10b) and the median bias is improved more by updating chemistry than horizontal resolution (Figure 9). Not all processes necessarily improve with finer horizontal resolution. As expected, biases in anthropogenic ozone precursors like CO and NO_x_ clearly decrease, but other biases (e.g., isoprene and water vapor) increase as shown in Figure 9. The impact of these competing processes may counteract each other leading to only minor net changes in ozone when using finer horizontal resolution. As described below, improvements to model processes including meteorology (Section 4.1), emissions (Section 4.2), and chemistry (Section 4.3) are needed to reduce systematic biases in CESM/CAM‐chem‐SE across all scales.

### 4.1 Meteorology

As shown in Figure 9, large biases exist in CESM/CAM‐chem‐SE for key meteorological variables important for atmospheric chemistry including temperature, water vapor, clouds (as evaluated by photolysis rates), and the planetary boundary layer (PBL) height (PBLH). The high temperature bias in CESM seems to impact isoprene emissions more at finer horizontal resolution (Figure 9). Additionally, photolysis rates of NO_2_ and O_3_ are too high and the water (H_2_O) vapor mixing ratio is too low (Figure 9). Combined this possibly suggests there are not enough low clouds in the model, which would increase the water vapor, lower the photolysis rate constants, and likely lower the temperature as well.

**Figure 11:**
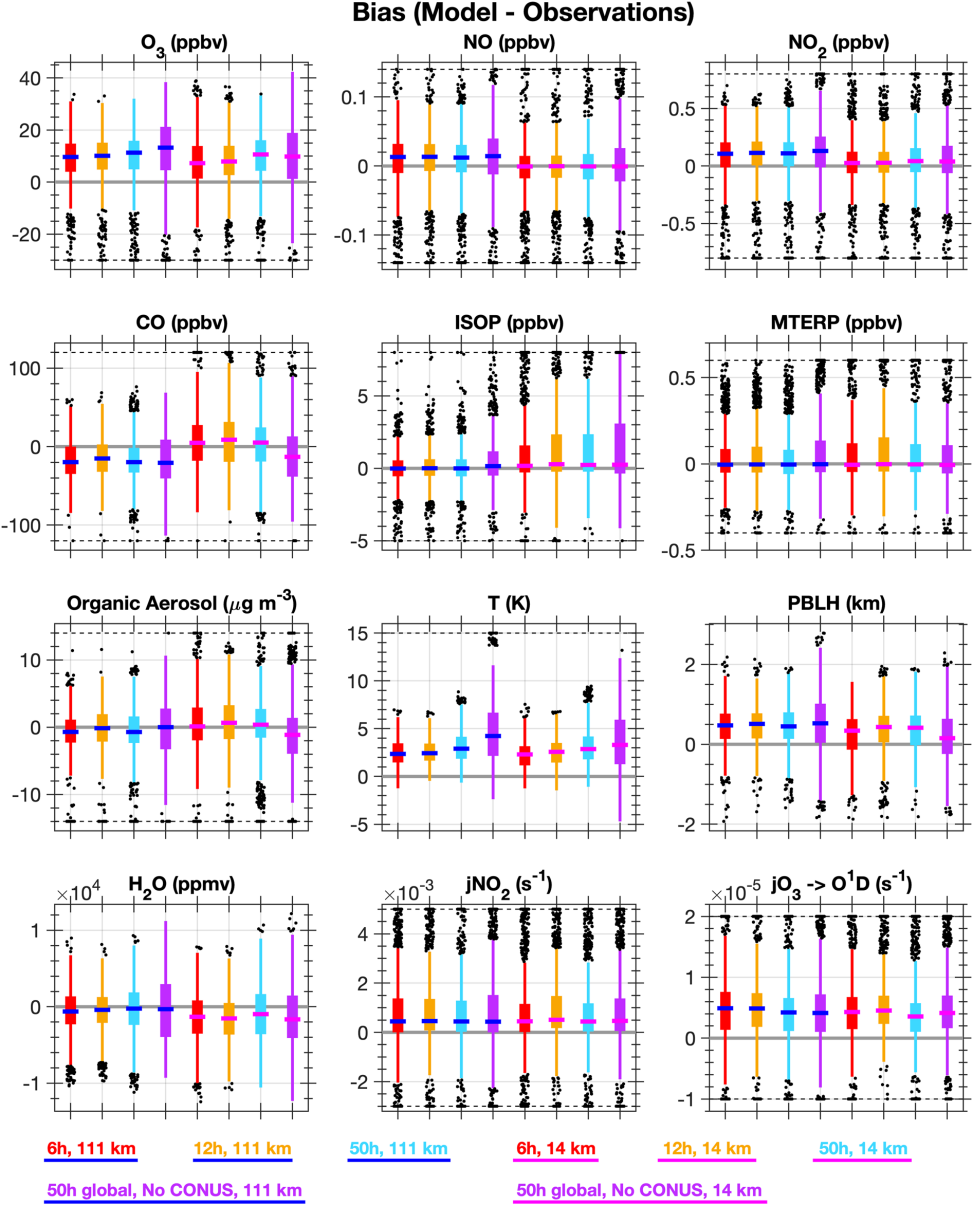
Identical to Figure 9, but for the specified dynamics sensitivity simulations listed in Table 1. The box and whiskers are colored by relaxation time: 6 hr (red), 12 hr (gold), 50 hr (light blue), and 50 hr globally, but no nudging within the conterminous US region (purple). The medians are colored by the horizontal resolution: ∼111 km (blue line) and ∼14 km (magenta line).

Given these biases, a series of sensitivity simulations (Table 1) are performed to evaluate the impact of the specified dynamics nudging strength. Temperature and winds are nudged to the GEOS5 meteorology (Section 2.2). The finest horizontal resolution used in this study (∼14 km, ∼0.125°) is finer than that available in the GEOS5 meteorology (0.31° × 0.25°). Thus, the base simulations to evaluate updates to chemistry and horizontal resolution are all performed using a light nudging with a relaxation time of 50 hr, in order to achieve the full benefit of using finer horizontal resolution. Box and whisker plots for the bias (model‐observations) along the SEAC^4^RS flight tracks below 1 km altitude at both ∼111 km (blue median lines) and ∼14 km (magenta median lines) horizontal resolutions are shown in Figure 11 for the following specified dynamics sensitivity tests: 6‐hr relaxation time, 12‐hr relaxation time, 50‐hr relaxation time, and no nudging over CONUS, but a 50‐hr relaxation time everywhere else. A 6‐hr relaxation time is a strong nudging case where full nudging toward reanalysis occurs every 6 hr, but distributed fractionally over each model time step. The stronger nudging to temperature and winds reduces the model bias in temperature for both horizontal resolutions, but even with a 6‐hr relaxation time the model is still biased high for temperature. At ∼14 km horizontal resolution, using the 6‐hr nudging results in the lowest bias for isoprene and ozone likely due to reductions in the temperature bias.

**Figure 12:**
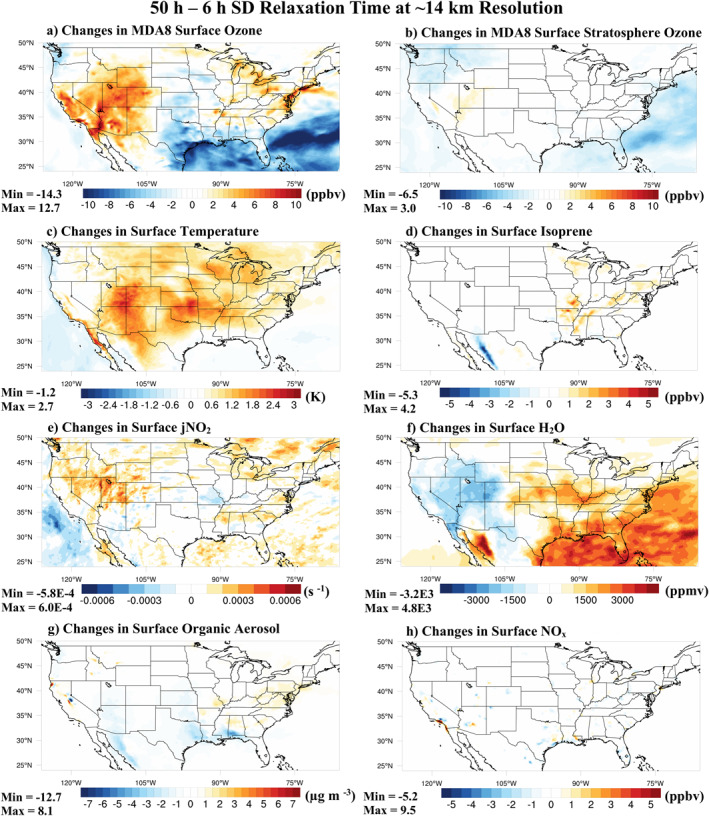
Changes at the surface caused by using a 50‐hr minus a 6‐hr specified dynamics (SD) relaxation time with the TS2.1 chemical mechanism at ∼14 km horizontal resolution for (a) MDA8 surface ozone, (b) MDA8 surface stratospheric ozone tracer, (c) temperature, (d) isoprene, (e) photolysis rate of NO_2_, (f) water mixing ratio, (g) organic aerosol, and (h) NO_x_.

Clear regional differences exist in MDA8 surface ozone when using 6‐hr versus 50‐hr relaxation times as shown in Figure 12a at ∼14 km and Figure S13a in Supporting Information S1 at ∼111 km horizontal resolution. There are negligible differences between the 6 and 50‐hr nudging simulations for the stratospheric ozone tracer at the surface (Figure 12b and Figure S13b in Supporting Information S1) verifying that the differences in surface MDA8 ozone are not caused by changes in troposphere‐stratosphere exchange. Isoprene concentrations (Figure 12d and Figure S13d in Supporting Information S1) and emissions (Figures S14a and S15a in Supporting Information S1) in the Southeast US are higher using the 50‐hr compared with the 6‐hr relaxation time especially at ∼14 km horizontal resolution. There are also clear differences in clouds (Figures S14c and S15c in Supporting Information S1) and thereby photolysis of NO_2_ (Figure 12e and Figure S13e in Supporting Information S1).

Interestingly, the model bias in water vapor is worse at the finer horizontal resolution (Figure 9) and changes in water vapor due to nudging strength (Figure 12f and Figure S13f in Supporting Information S1) are generally inversely correlated with changes in ozone. The impact of water vapor on ozone production and loss is complicated. Photolysis of O_3_ leads to the production of O(^1^D) (R1). O(^1^D) either reacts with H_2_O to destroy O_3_ (R2) or with O_2_ or N_2_ to form O(^3^P) (R3), which generally reacts with O_2_ to reform O_3_ (R4). In remote regions, higher water vapor leads to a net reduction in tropospheric ozone due to the competition between reactions 2 and 3 (Jacob & Winner, 2009; Johnson et al., 1999). In regions with NO_x_ pollution, the impact is more complex. For example, OH generated from reaction 2 can react with CO and VOCs in the presence of NO_x_ and sunlight to produce O_3_ or react with NO_2_ to produce HNO_3_ generally preventing O_3_ production (Jacob & Winner, 2009; Monks et al., 2015). The low model bias in water vapor using the ∼14 km horizontal resolution shown in Figure 9 falsely reduces reaction 2 biasing the O_3_ loss term low. Falsely reducing reaction 2 also reduces OH generation, which in the Southeast US where there is modest NO_x_ pollution biases the O_3_ production term low too. Due to this complexity, careful examination and reduction of biases in meteorology metrics like temperature, water vapor, and photolysis rates are important for accurately simulating ozone production and loss terms.

(R1) O3+hv→O2+O(D1)

(R2) O(D1)+H2O→OH+OH

(R3) O(D1)+O2/N2→O(P3)+O2/N2

(R4) O(P3)+O2+M→O3+M

The PBLH is over‐predicted in all simulations (Figures 9 and 11). CESM/CAM‐chem only has 32 vertical levels extending from the surface into the lower stratosphere (∼40 km). Increasing the vertical resolution would require re‐tuning many of the parameterizations in CESM/CAM‐chem. Regional models typically have many more vertical levels especially in the PBL. Interestingly, as shown in Figure 11, using different specified dynamics options does not impact the PBLH at ∼111 km resolution, but at ∼14 km resolution the PBLH is best represented when no nudging occurs over the CONUS region. Further investigation of how nudging temperature and winds impact the PBL parameterizations in CESM/CAM‐chem would also be beneficial. The CESM community is moving toward adding additional vertical levels in the PBL (Simpson et al., 2020), which will hopefully improve the simulated PBLH and the chemical and physical processes within the PBL.

The selection of nudging strength to temperature and winds strongly impacts other aspects of the model such as clouds, photolysis, water vapor, isoprene, and ozone. For example, in some regions differences in MDA8 ozone caused by selecting a 50‐hr versus 6‐hr relaxation time (Figure 12a) are similar in magnitude to using finer horizontal resolution or more complex chemistry (Figure 1). While nudging is needed to reduce dynamical variability, nudging can also lead to spurious results especially when the climatologies of the reanalysis meteorology and model are different (N. A. Davis et al., 2020). Development and evaluation of new nudging techniques that account for differences in the climatologies between the model and reanalysis meteorology (e.g., nudging toward climatological anomalies as done by N. A. Davis et al. (2020)) are needed. Future work to improve ozone predictions in CESM/CAM‐chem should prioritize reducing systematic regional biases in temperature rather than relying on specified dynamics to correct biases. In general, CESM/CAM‐chem has been tuned at coarser resolution, so development of scale‐independent parameterizations and tuning parameters are likely needed to reduce biases in meteorological variables. Additionally, the ability to test parameterizations developed for mesoscale processes within the MUSICA framework will also be useful.

### 4.2 Emissions

Reductions in biases of anthropogenic pollutants like NO, NO_2_, and CO are achieved most by improvements to finer horizontal resolution. There is still a high bias in NO_2_ in CESM/CAM‐chem even for the most complex simulation (TS2.1 chemistry and ∼14 km horizontal resolution). Generally, in the Southeast US NO_x_ is underpredicted in urban regions (e.g., St. Louis and Indianapolis in SENEX) and elsewhere large variability exists in the NO_x_ bias favoring a general overprediction of NO_x_ (Figures 10c and 10d). Higher resolution than ∼14 km may be needed to accurately capture NO_x_ in urban and urban outflow regions. Simulations at ∼111 km horizontal resolution generally had higher biases in NO_x_ than those at ∼14 km, but interestingly in certain locations the NO_x_ bias was higher at ∼14 km (Figures S18 and S19 in Supporting Information S1). This mixture of improvement in simulated NO_x_ is likely a contributing factor for why the median ozone vertical profile (Figure 4) did not greatly differ between the coarse and fine horizontal resolutions.

The high bias in the median and 25th and 75th percentiles for NO_x_ (Figure 4) combined with the spatial pattern of NO_x_ biases in Figure 10 suggest the need for additional capabilities within the MUSICA framework to replace sections of global anthropogenic emission inventories with regional inventories like the US EPA NEI inventory. The CAMS anthropogenic emissions used in this work are available at fine horizontal resolution, but only on a monthly timescale. The NEI inventory, along with having improved regional skill, would also add diel variation, weekend/weekday effects, and vertical information to the anthropogenic emissions. These improvements will likely reduce NO_x_ emissions and improve comparisons to observations shown in Section 3.2. Possibly further adjustments to these regional inventories will also be needed (e.g., NO_x_ reductions from mobile sources (McDonald, McKeen, et al., 2018; Travis et al., 2016)). With the new mesh refinement capability in CESM/CAM‐chem, evaluation of emission updates can easily be done across multiple scales, which will lead to more cohesive model improvements at the global and regional scale.

Additionally, a global soil NO_x_ inventory at 0.5° × 0.5° horizontal resolution is used in this work. Updating the resolution of soil NO_x_ emissions in CAM‐chem may also improve comparisons of modeled NO_x_ and ozone against field campaign observations (Figure 4). This should include incorporating updates from recent regional modeling studies (Almaraz et al., 2018; Oikawa et al., 2015; Souri et al., 2016) that identify the importance of increasing soil NO_x_ emissions in biogenic inventories such as MEGAN and BEIS (Biogenic Emission Inventory System).

The dust emissions used in CESM/CAM‐chem‐SE are based on a parameterization that uses a soil erodibility map that is at coarse (2° × 2°) horizontal resolution (Albani et al., 2014). Development of dust emission schemes that do not require resolution dependent soil erodibility maps and include other scale aware parameterizations (e.g., accurately handling higher wind speeds that are resolved at finer horizontal resolution) are underway in CAM and will be especially important for future CAM‐chem simulations with mesh refinement over regions of the Earth where dust emissions are more prevalent than CONUS. Although not the focus of this analysis, further constraints and evaluation of fire emissions, which vary widely between different emission inventories (e.g., Al‐Saadi et al., 2008; Pan et al., 2020; Pereira et al., 2016), fire plume injection height, and transport of smoke are also needed to accurately simulate ozone production from wildfires in CESM/CAM‐chem‐SE (Jaffe & Wigder, 2012).

Although this work has focused more on evaluating and discussing biases in emissions, deposition of ozone itself (Clifton et al., 2020) and VOC oxidation products (Nguyen et al., 2015) including ozone precursors and NO_x_ reservoir compounds are also important for accurately simulating ozone. Future work evaluating and improving deposition especially at a process level (e.g., Clifton et al., 2017; Kavassalis & Murphy, 2017) is also recommended.

#### 4.2.1 Isoprene Emissions

Isoprene and monoterpenes are unique in that model biases are lower using the ∼111 km versus ∼14 km horizontal resolution (Figures 5 and 9). As described in Section 2.1, biogenic emissions are calculated online in the CLM using MEGANv2.1 such that any changes in meterological data such as temperature will directly impact the emission rates. The total surface emissions of isoprene over CONUS are higher at ∼14 km than at ∼111 km horizontal resolution (Table 2).

Given the high temperature bias in CESM/CAM‐chem in all the simulations (Figure 9) and that isoprene emissions calculated by MEGAN v2.1 are highly sensitive to fluctuations in temperature (Guenther et al., 2012), isoprene emissions appear to be more sensitive to the high temperature bias in CESM/CAM‐chem at finer horizontal resolution. Part of the reason for performing the specified dynamics sensitivity tests was to determine if the difference in isoprene emissions between using ∼14 km compared to ∼111 km horizontal resolution could be mediated with stronger nudging, which partially corrects the high temperature bias in CESM. As shown in Figure 11, at ∼111 km horizontal resolution, the biases in isoprene are highest when no nudging is used within the CONUS region, but are not sensitive to the strength of nudging (i.e., 50 and 6 hr relaxation times have similar biases in isoprene). At ∼14 km horizontal resolution, no nudging within the CONUS region produced the highest biases for isoprene. Unlike at ∼111 km, increasing the nudging strength reduced the biases in isoprene at ∼14 km horizontal resolution with the 6 hr relaxation time performing the best. For now, these results suggest that users should use a stronger nudging strength especially in regions heavily impacted by biogenic emissions until the high temperature bias in CESM is addressed. CESM like other climate models often has higher regional temperature biases (e.g., Brown‐Steiner et al., 2015; Rasmussen et al., 2012) than weather models (e.g., Abdi‐Oskouei et al., 2020; Emery et al., 2001; McDonald, McKeen, et al., 2018). We acknowledge this is one limitation of bringing a climate model to regional scales, but future improvements including scale‐independent parameterizations and tuning parameters will hopefully improve temperature biases at all scales. Additionally, Jo et al. (2021) reduced the isoprene emissions for tropical PFTs in CESM in order to scale the global isoprene emissions down to be consistent with Bauwens et al. (2016). Future work will investigate how much model temperature biases versus other factors contribute to model biases in isoprene.

To better understand whether the increase in isoprene (Table S5 and Figures 5, 9, and S5 in Supporting Information S1) when using ∼14 km compared to ∼111 km horizontal resolution is caused by changes in oxidants or emissions, the area‐weighted isoprene emissions averaged over August 2013 for the Southeast US are compared. As expected, isoprene emissions do not change with changes to chemistry at both resolutions (i.e., factor of 1.01 from TS1 to TS2.1). Conversely, using ∼14 km compared to ∼111 km horizontal resolution, increases the isoprene emissions by a factor of 1.6 for both chemical mechanisms. In comparison, the area‐weighted average surface isoprene concentration increases by a factor of 2.3 when using ∼14 km compared to ∼111 km horizontal resolution for both chemical mechanisms. Because the changes in the surface isoprene concentration are higher than the changes in isoprene emissions, both direct and indirect effects are likely contributing to the differences in isoprene concentrations between the different horizontal resolutions. Higher isoprene emissions are increasing the isoprene concentrations directly, but also indirectly by increasing OH consumption regionally, which leads to less isoprene oxidation, and higher isoprene concentrations. Additionally, improved spatial segregation of isoprene and NO_x_ at ∼14 km resolution may also change regional oxidant concentrations. For example, K. Yu et al. (2016) found isoprene concentrations increase at finer horizontal resolution even when isoprene emissions are kept constant.

The isoprene bias at ∼14 km horizontal resolution is especially important in Louisiana, Arkansas, and Missouri (Figures S20 and S21 in Supporting Information S1). These same regions have fairly high values of isoprene in general and as expected are the regions where updating the biogenic chemistry (TS2.1) reduced the ozone bias (Figures S16 and S17 in Supporting Information S1). Ozone is often overpredicted (Figures 10a and 10b) where isoprene is also biased high (Figures 10e and 10f), but most often the highest biases in ozone do not correspond to where isoprene is also biased high. Thus, improving the biases in isoprene emissions, while important, may have a limited impact on the general ozone bias in the Southeast US in CESM/CAM‐chem‐SE.

### 4.3 Chemistry

In the ambient atmosphere gas and aerosol chemistry are intrinsically linked, but gas and aerosol processes are often represented separately in 3D models. Better understanding and incorporating gas and aerosol feedbacks into models will be crucial for accurately representing ozone and aerosols in the future. This includes adding the impact of aerosols on photolysis rates into CESM. Additionally, more complex representation of heterogenous chemistry is needed both for inorganic compounds (Jacob, 2000) as well as VOC oxidation products. For example, the loss of tertiary organic nitrates to aerosol and clouds and subsequent hydrolysis is an important NO_x_ loss pathway as demonstrated by novel experimental work (Darer et al., 2011; Hu et al., 2011; Jacobs et al., 2014; Teng et al., 2017) and recently applied to several 3D atmospheric chemistry models (Bates & Jacob, 2019; Müller et al., 2019; Schwantes et al., 2020; Zare et al., 2018, 2019). In particular, recent work by Vasquez et al. (2020) provides further constraints on the atmospheric fate of the different IHN isomers. Loss of the dominant tertiary IHN by aerosol uptake and subsequent hydrolysis is on par with OH‐oxidation even in Pasadena, CA, with low relative humidity and high OH and even more important over forested regions with high relative humidity and low OH. These past experimental and modeling studies emphasize the importance of expanding the representation of heterogenous chemistry in CESM/CAM‐chem to better represent the loss pathways of organic nitrates to aerosols and clouds in the future.

Using TS2.1 compared to TS1 chemistry reduces the positive model bias in organic aerosol during the SEAC^4^RS campaign, but causes a negative model bias during the SENEX campaign (Figure 7, Figures S22 and S23 in Supporting Information S1). In particular, organic aerosol is underpredicted during the SENEX campaign especially in urban and urban outflow regions (Figure 10h) suggesting urban SOA is not well represented in CESM/CAM‐chem. CESM/CAM‐chem is likely missing emerging urban SOA sources such as VOCs from volatile chemical products (VCPs) or cooking (Klein et al., 2016; McDonald, de Gouw, et al., 2018; Nault et al., 2021; Qin et al., 2021). Past studies have also suggested that biogenic emission inventories often, but not always underpredict isoprene in urban regions (Fast et al., 2014; Knote et al., 2014; Kota et al., 2015; Ma et al., 2019, 2022; Zhao et al., 2016), which may also be contributing to the underprediction of urban SOA in CESM/CAM‐chem. Additionally, finer horizontal resolution than ∼14 km may be needed to resolve the urban and power plant plumes sampled during the SENEX campaign (Warneke et al., 2016). Improving the representation of SOA in urban and urban outflow regions in CESM/CAM‐chem including performing simulations at even finer horizontal resolution and updates to emissions and chemistry to include emerging emission sources such as those from VCPs and cooking should be prioritized in future work. Additionally, MAM4 does not simulate inorganic particulate nitrate. Incorporation of inorganic particulate nitrate through Model for Simulating Aerosol Interactions and Chemistry into CESM is currently under development.

In general, increasing chemical complexity increases the number of transported tracers, which then increases the simulation cost. How computational cost scales with adding additional transported tracers depends on the transport scheme. For uniform grids in CESM/CAM‐chem‐SE, users can optionally use the CSLAM (Conservative Semi‐Lagrangian Multitracer) scheme, which improves the efficiency and accuracy of tracer transport (Lauritzen et al., 2017) compared to the default transport scheme. The uniform resolution configuration using CSLAM passes the state to physical parameterizations on a more “equal‐area” grid compared to the spectral‐element quadrature grid. This alleviates spurious noise near the element boundaries (Herrington, Lauritzen, Taylor, et al., 2019) and allows for running parameterizations on a lower or higher resolution grid compared to the dynamics (Herrington, Lauritzen, Reed, et al., 2019). Currently, the variable resolution configurations do not support CSLAM and the separate physics grid, but there are no theoretical constraints preventing this development. Further development of computationally efficient transport schemes like CSLAM will be particularly useful for predicting air quality, which requires a large number of transported chemical tracers, within the MUSICA framework.

## 5 Future Model Development

This work brings a global atmospheric chemistry model, CESM/CAM‐chem, to regional scales through mesh refinement using the SE dynamical core. Based on the evaluation described in Section 3 and 4, several capabilities have been identified as important priorities for future work. Those with the highest priority are:

1. Add capability to replace the CAMS global anthropogenic emission inventory in specific locations with regional inventories that have improved local information and greater detail such as weekend/weekday effects, diel variation, and vertical information, which will include first adding the US EPA NEI inventory relevant over CONUS and second a more generalized approach to use regional emission inventories across the world. This will be facilitated by the use of the Harmonized Emissions Component, which is being connected to CESM2 (H. Lin et al., 2021).
2. Update the resolution of soil NO_x_ and dust emissions used in CAM‐chem.
3. Improve feedbacks between gas and aerosols in CESM/CAM‐chem including incorporating the feedback of aerosols on photolysis rates and improving the representation of heterogeneous chemistry including the loss of organic nitrates to aerosols and clouds.
4. Further develop computationally efficient transport schemes like CSLAM, which will limit the computational costs of using more complex chemical mechanisms.
5. Improve understanding and reduction of regional biases in meteorology in CAM‐chem, especially temperature, water vapor, clouds, and PBL height, which includes:
  1. Developing scale‐independent parameterizations and tuning factors.
  2. Adding the capability to test parameterizations developed for mesoscale processes within MUSICA.
  3. Increasing the vertical resolution within the PBL.
6. Incorporate a nonhydrostatic dynamical core into CESM, in order to achieve an objective of MUSICA to model from global to local scales all within the same modeling framework.
7. Develop and evaluate new grids with regional refinement in various regions around the world.

## 6 Conclusions

A new configuration of CESM/CAM‐chem called CESM/CAM‐chem‐SE with the capability of horizontal mesh refinement has been developed and evaluated in this work. This new capability not only creates a model ideally suited to simulate air quality across scales including global feedbacks without the limitations of lateral BCs, but also allows studies like this one to evaluate the importance of horizontal resolution and chemical complexity all within a single model framework.

Even with finer horizontal resolution and more complex chemistry, biases in ozone remain as compared to observations from five aircraft campaigns in 2013. The aircraft observations provide a wide variety of measurements of ozone precursors (VOCs, CO, and NO_x_) and meteorological metrics (water vapor, temperature, photolysis rate constants, and PBLH), which enable a more careful examination of model processes. In particular, this analysis suggests further work reducing biases in meteorology like temperature, water vapor, clouds, and PBLH will be important for improving simulated ozone in CESM/CAM‐chem‐SE. Because inter‐model comparisons typically have better agreement for the ozone burden than for ozone production and loss terms (Young et al., 2018), continued evaluation against field campaign data with comprehensive measurement payloads is important for understanding whether the model physical and chemical processes, which determine the ozone distribution, are accurately represented.

We recognize that several regional model capabilities still need to be incorporated into CESM as described in Section 5 including updates to anthropogenic emissions, soil NO_x_ emissions, dust emissions, vertical resolution, and better coupling gas and aerosol chemistry. Additionally, various chemical and physical processes in CESM need to be updated to be scale‐independent and/or scale‐aware (Pfister et al., 2020). These activities will be a priority for future work.

This work demonstrates the importance of balancing the simulation costs of horizontal resolution, vertical resolution, and physical and chemical processes complexity. Ozone precursors are all more impacted by horizontal resolution than ozone itself suggesting that ozone formation processes clearly change when using ∼14 km compared to ∼111 km horizontal resolution. Future studies investigating fine scale features such as urban and fire plumes will gain the most benefit from using the mesh refinement capability added to CESM/CAM‐chem in this work. Updating isoprene and terpene chemistry reduces surface ozone in the Eastern US at varying horizontal resolutions and especially at finer horizontal resolution where a greater range of NO_x_ and VOC chemical regimes are resolved.

Because the impact of using more complex isoprene and terpene chemistry on ozone and other compounds such as formaldehyde and isoprene oxidation products is more pronounced at finer horizontal resolution, more complex chemistry is needed to achieve the full benefit of using finer horizontal resolution. This demonstrates the importance of balancing increases in finer horizontal resolution with model physical and chemical process complexity. Model process complexity often only increases simulation costs by fractional amounts compared to increases in horizontal resolution, which increase simulation costs by orders of magnitude. A CESM/CAM‐chem‐SE simulation using the grid with regional refinement down to ∼14 km over CONUS is a factor of ∼29 more expensive than that using the uniform ∼111 km resolution and comparable in cost to a WRF‐Chem simulation over CONUS after roughly accounting for differences in the chemical/physical timestep and the number of grid cells and tracers used. For comparison, using TS2.1 chemistry increases the cost by a factor of ∼1.6 compared with using TS1 chemistry in CESM2.2/CAM‐chem‐SE.

This is only the beginning of the community effort called MUSICA to create a unified infrastructure to model atmospheric chemistry across local to regional to global scales in the Earth system (Pfister et al., 2020). Connecting the local scale will be an important goal for future work to simulate atmospheric chemistry in urban environments to fire plumes to forest canopies. Although moving to finer and finer horizontal resolutions to capture fine scale features is important, results from this work suggest improvements to model physical and chemical processes and vertical resolution are equally important for reducing model biases of ozone. Now that CESM has the capability of moving seamlessly between regional and global scales, model processes can be more extensively evaluated and improved, which will hopefully lead to future improvements in model performance at simulating ozone across all scales.

## Supporting information

Supporting Information S1

## Data Availability

*Model Code Availability*: Community Earth System Model (CESM) is an open‐source community model available from http://www.cesm.ucar.edu/. The regional refined capability over conterminous US developed in this work was released as a component set in CESM2.2. The following webpage describes how to download and use CESM2.2 (https://wiki.ucar.edu/display/MUSICA/MUSICA+Home). The TS2.1 and TS1.1 chemical mechanisms are also available in the latest release of CESM2.2. The TS2.1 mechanism includes the gas‐phase updates to MOZART‐TS2 (Schwantes et al., 2020) and secondary organic aerosol (SOA) mechanism updates applied here. Considering the importance of these SOA mechanism updates at all scales as demonstrated in this work, similar updates were applied to the MOZART‐TS1 (Emmons et al., 2020) chemical mechanism to create TS1.1. The CESM namelist files used to perform the simulations listed in Table 1 are provided at https://github.com/rschwant/cesm_cam_chem_se_evaluation_aircraft_schwantes. *Model Data Availability*: To facilitate further evaluation of this data set, the unprocessed model flight track data including all vertical levels and the closest nine grid cells to each observational point are provided online along with global monthly averaged data for August 2013 at https://doi.org/10.5065/wtcc-at83 (Schwantes et al., 2022). Data from a new simulation of MUSICAv0 for 2013 with code updates beyond that used in this work are also available for community use here https://doi.org/10.5065/tgbj-yv18 (Tilmes, 2021). *Observational Data Availability*: The field campaign data used in this work are available for download at the following websites: Studies of Emissions, Atmospheric Composition, Clouds, and Climate Coupling by Regional Surveys Revision 7 (https://www-air.larc.nasa.gov/cgi-bin/ArcView/seac4rs?MERGE=1#60_SECOND.DC8_MRG/; NASA, 2018), Southeast Nexus Revision D (https://esrl.noaa.gov/csd/groups/csd7/measurements/2013senex/P3/DataDownload/mergeFiles.html), Nitrogen, Oxidants, Mercury and Aerosol Distributions, Sources, and Sinks (NOMADSS) Revision 5 (https://data.eol.ucar.edu/dataset/373.046; Emmons, 2016), and DISCOVER‐AQ‐CA Revision 4 and DISCOVER‐AQ‐TX Revision 3 (https://www-air.larc.nasa.gov/missions/merges/; NASA, 2014). The NOMADSS data is provided by the NCAR/EOL under the sponsorship of the National Science Foundation (https://data.eol.ucar.edu/) and part of the larger Southeast Atmosphere Study data set (https://data.eol.ucar.edu/master_lists/generated/sas/).
