# Supplementary material for: Evaluating the Impact of Chemical Complexity and Horizontal Resolution on Tropospheric Ozone Over the Conterminous US With a Global Variable Resolution Chemistry Model
Source: J Adv Model Earth Syst. 2022 Jun 22;14(6):e2021MS002889. doi: 10.1029/2021MS002889 (PMC9286600; doi:10.1029/2021MS002889)
Supplement: Supplementary file 1 — Supporting Information S1 [file JAME-14-0-s001.pdf]

Supporting Information for "Evaluating the impact of chemical complexity and horizontal resolution on tropospheric ozone over the conterminous US with a global variable resolution chemistry model"

Rebecca H. Schwantes[1,2,3], Forrest G. Lacey[1], Simone Tilmes[1], Louisa K. Emmons[1], Peter H. Lauritzen[4], Stacy Walters[1], Patrick Callaghan[4], Colin M. Zarzycki[5], Mary C. Barth[1], Duseong S. Jo[1], Julio T. Bacmeister[4], Richard B. Neale[4], Francis Vitt[1], Erik Kluzek[4], Behrooz Roozitalab[6], Samuel R. Hall[1], Kirk Ullmann[1], Carsten Warneke[3], Jeff Peischl[2,3], Ilana B. Pollack[7], Frank Flocke[1], Glenn M. Wolfe[8], Thomas F. Hanisco[8], Frank N. Keutsch[9], Jennifer Kaiser[10], Thao Paul V. Bui[11], Jose L. Jimenez[12,2], Pedro Campuzano-Jost[12,2], Eric C. Apel[1], Rebecca S. Hornbrook[1], Alan J. Hills[1], Bin Yuan[13], Armin Wisthaler[14,15]

[1]Atmospheric Chemistry Observations & Modeling Laboratory, National Center for Atmospheric Research, Boulder, CO, USA

[2]Cooperative Institute for Research in Environmental Sciences, University of Colorado, Boulder, CO, USA

[3]Chemical Sciences Laboratory, National Oceanic and Atmospheric Administration, Boulder, CO, USA

[4]Climate and Global Dynamics Laboratory, National Center for Atmospheric Research, Boulder, CO, USA

[5]Department of Meteorology and Climate Dynamics, Pennsylvania State University, University Park, PA, USA

[6]Department of Chemical and Biochemical Engineering & Center for Global and Regional Environmental Research, The University of Iowa, Iowa City, IA, USA

[7]Department of Atmospheric Science, Colorado State University, Fort Collins, CO, USA

[8]Atmospheric Chemistry and Dynamics Lab, NASA Goddard Space Flight Center, Greenbelt, MD, USA

[9]John A. Paulson School of Engineering and Applied Sciences, Department of Chemistry and Chemical Biology & Department of Earth and Planetary Sciences, Harvard University, Cambridge, MA, USA

[10]School of Civil and Environmental Engineering & School of Earth and Atmospheric Sciences, Georgia Institute of Technology, Atlanta, GA, USA

[11]Earth Science Division, NASA Ames Research Center, Moffett Field, CA, USA

[12]Department of Chemistry, University of Colorado, Boulder, CO, USA

[13]Institute for Environmental and Climate Research, Jinan University, Guangzhou 511443, China

[14]Institute for Ion Physics and Applied Physics, University of Innsbruck, Technikerstrasse 25, 6020 Innsbruck, Austria

[15]Department of Chemistry, University of Oslo, P.O. 1033 – Blindern, 0315 Oslo, Norway

Contents of this file

1. Tables S1 to S5
2. Figures S1 to S23

Introduction A list of the new species and reactions added to the MOZART-TS2 mechanism to create the MOZART-TS2.1 mechanism are provided in Tables S1 and S2, respectively. Tables S3 and S4 provide a list of the observations used in this work for model evaluation. Table S5 lists the differences in the area-weighted averages during August 2013 at the surface over the same regions defined in the field campaign analysis as shown in Figure S1.

Figure S1 displays the flight tracks from the five 2013 aircraft campaigns used to evaluate the model in this work. Difference plots similar to Figures 1-3 in the main text,

but extended to more compounds (nitrogen oxides (NO_x), carbon monoxide (CO), hydrogen oxide radicals (HO_x), isoprene, isoprene hydroxy hydroperoxides, isoprene hydroxy nitrates, methacrolein, and methyl vinyl ketone) are presented in Figures S2-S9. Figure S10 is similar to Figure 4 in the main text, but the flight tracks are processed differently; no horizontal interpolation is applied and instead the model grid cell closest in distance (based on the grid cell center) to each observational point is selected. Figure S11 and S12 provide the median vertical profile plots for the SEAC^{4}RS campaign similar to Figures 4 - 8 in the main text, but extended up to 12 km in pressure altitude. Figures S13 - S15 are similar to Figure 12 in the main text, but extended to include more compounds and also results for simulations using $\sim$ 111 km horizontal resolution. Figure S16 - S23 are similar to Figure 10 in the main text, but extended to include all four main model simulations.

References

Carlton, A. G., de Gouw, J., Jimenez, J. L., Ambrose, J. L., Attwood, A. R., Brown, S., ... Zhou, X. (2018). Synthesis of the Southeast Atmosphere Studies: Investigating fundamental atmospheric chemistry questions. *Bulletin of the American Meteorological Society*, *99*, 547-567. doi: 10.1175/BAMS-D-16-0048.1

Crawford, J. H., & Pickering, K. E. (2014). DISCOVER-AQ: Advancing strategies for air quality observations in the next decade. *Air and Waste Management Association EM*, 4-7.

Toon, O. B., Maring, H., Dibb, J., Ferrare, R., Jacob, D. J., Jensen, E. J., ... Pszenny, A. (2016). Planning, implementation, and scientific goals of the Studies of Emissions and Atmospheric Composition, Clouds and Climate Coupling by Regional Surveys (SEAC^{4}RS) field mission. *Journal of Geophysical Research: Atmospheres*, *121*, 4967-

5009. doi: 10.1002/2015JD024297

Warneke, C., Trainer, M., de Gouw, J. A., Parrish, D. D., Fahey, D. W., Ravishankara, A. R., ... Hatch, C. D. (2016). Instrumentation and measurement strategy for the NOAA SENEX aircraft campaign as part of the Southeast Atmosphere Study 2013. *Atmospheric Measurement Techniques*, *9*, 3063-3093. doi: 10.5194/amt-9-3063-2016

Table S1. New Species added to MOZART-TS2 to create MOZART-TS2.1

Species Name	Molecular Formula	Description
APINO2VBS	C10H17O3	SOA tracer for α-pinene hydroxy peroxy radical
BCARYO2VBS	C15H25O3	SOA tracer for β-caryophyllene hydroxy peroxy radical
BENZO2VBS	C6H7O5	SOA tracer for bicyclic peroxy radical from benzene
BPINO2VBS	C10H17O3	SOA tracer for β-pinene hydroxy peroxy radical
ISOPO2VBS	C5H9O3	SOA tracer for isoprene hydroxy peroxy radical
IVOCO2VBS	C13H29O3	SOA tracer for IVOC hydroxy peroxy radical
LIMONO2VBS	C10H17O3	SOA tracer for limonene hydroxy peroxy radical
MYRCO2VBS	C10H17O3	SOA tracer for myrcene hydroxy peroxy radical
TOLUO2VBS	C7H9O5	SOA tracer for bicyclic peroxy radical from toluene
XYLEO2VBS	C8H11O5	SOA tracer for bicyclic peroxy radical from xylene

[a] All species listed in this table are peroxy radicals and so are not transported in the model.

Table S2. New Reactions added to MOZART-TS2 to create MOZART-TS2.1

Reactants	Products	Rate Constant
APIN + OH	APIN + OH + 1.0000*APINO2VBS	1.34e-11 exp(410/T)
APINO2VBS + HO2	HO2 + 0.0508*SOAG0 + 0.1149*SOAG1 + 0.0348*SOAG2 + 0.0554*SOAG3 + 0.1278*SOAG4	2.6e-13 exp(1300/T)
APINO2VBS + NO	NO + 0.0245*SOAG0 + 0.0082*SOAG1 + 0.0772*SOAG2 + 0.0332*SOAG3 + 0.1300*SOAG4	2.7e-12 exp(360/T)
BCARY + OH	BCARY + OH + 1.0000*BCARYO2VBS	2e-10
BCARYO2VBS + HO2	HO2 + 0.2202*SOAG0 + 0.2067*SOAG1 + 0.0653*SOAG2 + 0.1284*SOAG3 + 0.114*SOAG4	2.75e-13 exp(1300/T)
BCARYO2VBS + NO	NO + 0.1279*SOAG0 + 0.1792*SOAG1 + 0.0676*SOAG2 + 0.0790*SOAG3 + 0.1254*SOAG4	2.7e-12 exp(360/T)
BENZENE + OH	BENZENE + OH + 1.0000*BENZO2VBS	2.3e-12 exp(-193/T)
BENZO2VBS + HO2	HO2 + 0.0023*SOAG0 + 0.0008*SOAG1 + 0.0843*SOAG2 + 0.0443*SOAG3 + 0.1621*SOAG4	7.5e-13 exp(700/T)
BENZO2VBS + NO	NO + 0.0097*SOAG0 + 0.0034*SOAG1 + 0.1579*SOAG2 + 0.0059*SOAG3 + 0.0536*SOAG4	2.6e-12 exp(365/T)
BPIN + OH	BPIN + OH + 1.0000*BPINO2VBS	1.62e-11 exp(460/T)
BPINO2VBS + HO2	HO2 + 0.0508*SOAG0 + 0.1149*SOAG1 + 0.0348*SOAG2 + 0.0554*SOAG3 + 0.1278*SOAG4	2.6e-13 exp(1300/T)
BPINO2VBS + NO	NO + 0.0245*SOAG0 + 0.0082*SOAG1 + 0.0772*SOAG2 + 0.0332*SOAG3 + 0.1300*SOAG4	2.7e-12 exp(360/T)
ISOP + NO3	ISOP + NO3 + 0.059024*SOAG3 + 0.025024*SOAG4	2.95e-12 exp(-450/T)
ISOP + O3	ISOP + O3 + 0.0033*SOAG3	1.03e-14 exp(-1995/T)
ISOP + OH	ISOP + OH + 1.0000*ISOPO2VBS	2.7e-11 exp(390/T)
ISOPO2VBS + HO2	HO2 + 0.0031*SOAG0 + 0.0035*SOAG1 + 0.0003*SOAG2 + 0.0271*SOAG3 + 0.0474*SOAG4	2.12e-13 exp(1300/T)
ISOPO2VBS + NO	NO + 0.0003*SOAG0 + 0.0003*SOAG1 + 0.0073*SOAG2 + 0.0057*SOAG3 + 0.0623*SOAG4	2.7e-12 exp(360/T)
IVOC + OH	OH + 1.0000*IVOCO2VBS	1.34e-11
IVOCO2VBS + HO2	HO2 + 0.2381*SOAG0 + 0.1308*SOAG1 + 0.0348*SOAG2 + 0.0076*SOAG3 + 0.0113*SOAG4	7.5e-13 exp(700/T)
IVOCO2VBS + NO	NO + 0.1056*SOAG0 + 0.1026*SOAG1 + 0.0521*SOAG2 + 0.0143*SOAG3 + 0.0166*SOAG4	2.6e-12 exp(365/T)
LIMON + OH	LIMON + OH + 1.0000*LIMONO2VBS	3.41e-11 exp(470/T)
LIMONO2VBS + HO2	HO2 + 0.0508*SOAG0 + 0.1149*SOAG1 + 0.0348*SOAG2 + 0.0554*SOAG3 + 0.1278*SOAG4	2.6e-13 exp(1300/T)
LIMONO2VBS + NO	NO + 0.0245*SOAG0 + 0.0082*SOAG1 + 0.0772*SOAG2 + 0.0332*SOAG3 + 0.1300*SOAG4	2.7e-12 exp(360/T)
MYRC + OH	MYRC + OH + 1.0000*MYRCO2VBS	2.1e-10
MYRCO2VBS + HO2	HO2 + 0.0508*SOAG0 + 0.1149*SOAG1 + 0.0348*SOAG2 + 0.0554*SOAG3 + 0.1278*SOAG4	2.6e-13 exp(1300/T)
MYRCO2VBS + NO	NO + 0.0245*SOAG0 + 0.0082*SOAG1 + 0.0772*SOAG2 + 0.0332*SOAG3 + 0.1300*SOAG4	2.7e-12 exp(360/T)
TOLUENE + OH	TOLUENE + OH + 1.0000*TOLUO2VBS	1.7e-12 exp(352/T)
TOLUO2VBS + HO2	HO2 + 0.1364*SOAG0 + 0.0101*SOAG1 + 0.0763*SOAG2 + 0.2157*SOAG3 + 0.0738*SOAG4	7.5e-13 exp(700/T)
TOLUO2VBS + NO	NO + 0.0154*SOAG0 + 0.0452*SOAG1 + 0.0966*SOAG2 + 0.0073*SOAG3 + 0.2380*SOAG4	2.6e-12 exp(365/T)
XYLENES + OH	XYLENES + OH + 1.0000*XYLEO2VBS	1.7e-11
XYLEO2VBS + HO2	HO2 + 0.1677*SOAG0 + 0.0174*SOAG1 + 0.086*SOAG2 + 0.0512*SOAG3 + 0.1598*SOAG4	7.5e-13 exp(700/T)
XYLEO2VBS + NO	NO + 0.0063*SOAG0 + 0.0237*SOAG1 + 0.0025*SOAG2 + 0.0110*SOAG3 + 0.1185*SOAG4	2.6e-12 exp(365/T)

Table S3. Description of Observations for SEAC^{4}RS

Observation	Instrumentation (Uncertainties)	Affiliation: Contributors (email)
O_3, NO, NO_2	Chemiluminescence (O_3: 0.030 ppbv + 3%, NO: 0.010 ppbv + 4%, NO_2: 0.030 ppbv + 7%)	NOAA CSL: Thomas Ryerson (tbryerson@gmail.com), Ilana Pollack (ipollack@rams.colostate.edu), Jeff Peischl (jeff.peischl@noaa.gov)
CO	Diode laser spectrometer - DACOM (5% or 5 ppbv)	NASA LaRC: Glenn S. Diskin (glenn.s.diskin@nasa.gov)
H_2O	Diode Laser Hygrometer - DLH (5% or 1 ppmv)	NASA LaRC: Glenn S. Diskin (glenn.s.diskin@nasa.gov)
CH_2O	In Situ Airborne Formaldehyde (ISAF) - Laser Induced Fluorescence (10 pptv + 10%)	NASA GSFC: Thomas F. Hanisco (thomas.hanisco@nasa.gov) and Glenn M. Wolfe
Acetonitrile, Monoterpenes, Isoprene *	Proton Transfer Reaction Mass Spectrometer - PTR-MS (Acetonitrile: 15%, Monoterpenes: 15%, Isoprene: 5%)	University of Innsbruck: Armin Wisthaler (armin.wisthaler@uibk.ac.at)
jNO_2, $jO_3 \rightarrow O(^1D)$	Charged-coupled device Actinic Flux Spectroradiometer - CAFS (jNO_2: 12%, jO_3: 25%)	NCAR: Samuel R. Hall (halls@ucar.edu) and Kirk Ullmann
Organic Aerosol	Time-of-Flight Aerosol Mass Spectrometer - ToF-AMS (38%)	University of Colorado Boulder: Jose L Jimenez (jose.jimenez@colorado.edu) and Pedro Campuzano-Jost (pedro.campuzanojost-1@colorado.edu)
Isoprene oxidation products	Triple Quadrupole Chemical Ionization Mass Spectrometer for ISOPOOH (30 pptv + 40%) and Chemical Ionization Time of Flight Mass Spectrometer (CIT-ToF-CIMS) for $C_5O_3H_8$ (20 pptv + 50%), ISOPN (10 pptv + 30%), NOA (10 pptv + 50%), $C_4O_5H_7N$ (5 pptv + 30%), NO_3CH_2CHO (5 pptv + 50%)	California Institute of Technology: Paul Wennberg (wennberg@gps.caltech.edu), John Crounse, Jason St. Clair
Total Organic Nitrates and Total Peroxy Acyl Nitrates	Thermal-Dissociation Laser Induced Fluorescence - TD-LIF (Organic Nitrates: 15%, Peroxy Acyl Nitrates: 10%)	University of California, Berkeley: Ronald Cohen (cohen@cchem.berkeley.edu)
Temperature	MMS - Meteorological Measurement System (0.3K)	NASA Ames Research Center: T. Paul Bui (thaopaul.v.bui@nasa.gov)
PBLH **	Mixed layer height derived from Differential Absorption Lidar - DIAL High Spectral Resolution Lidar - HSRL (see README with data)	NASA LaRC: Richard Ferrare (Richard.A.Ferrare@nasa.gov), John Hair, Amy Jo Scarino

* Isoprene is listed as isoprenefuran. Because data impacted by fires are removed, the furan contribution should be minor.

** The mixed layer height (Best_ML_Hgt) was directly compared to the planetary boundary layer height (PBLH) model output. Data from 8/14/2013 were excluded due to an unexpectedly high value derived from the DIAL-HSRL, which was either a sampling artifact or an anomalous event that should not be included in this representative analysis.

Chemical acronyms: ozone (O_3), nitrogen oxide (NO), nitrogen dioxide (NO_2), carbon monoxide (CO), water (H_2O), formaldehyde (CH_2O), jNO_2 (NO_2 photolysis rate), $jO_3 \rightarrow O(^1D)$ (O_3 photolysis rate), isoprene hydroxy hydroperoxide (ISOPOOH), isoprene hydroperoxy aldehyde (HPALD), isoprene hydroxy nitrate (ISOPN), propanone nitrate (NOA), methacrolein and methyl vinyl ketone nitrates (MACRN + MVKN), ethanal nitrate (NO_3CH_2CHO)

Organization acronyms: NASA LaRC (National Aeronautics and Space Administration Langley Research Center), NOAA CSL (National Oceanic and Atmospheric Administration Chemical Sciences Laboratory), NASA GSFC (NASA Goddard Space Flight Center), and NCAR (National Center for Atmospheric Research)

For more information about the SEAC^{4}RS campaign and instrumentation refer to Toon et al. (2016) and DOI: 10.5067/Aircraft/SEAC4RS/Aerosol-TraceGas-Cloud

Table S4. Description of Observations for SENEX, NOMADSS, and DISCOVER-AQ

**Observation**	**Instrumentation (Uncertainties)**	**Affiliation: Contributors (email)**
**SENEX**		
O_3, NO, NO_2	Chemiluminescence (5%)	NOAA CSL: Ilana Pollack (ipollack@rams.colostate.edu) and Thomas Ryerson (tbryerson@gmail.com)
CO	Vacuum ultraviolet resonance fluorescence (5%)	CIRES (Cooperative Institute for Research in Environmental Sciences)/NOAA CSL: John S Holloway (john.holloway@colorado.edu)
CH_2O	In Situ Airborne Formaldehyde (ISAF) - Laser Induced Fluorescence (10 pptv + 10%)	NASA GSFC: Thomas F. Hanisco (thomas.hanisco@nasa.gov), Glenn M. Wolfe, Frank Keutch (keutsch@seas.harvard.edu), and Jennifer Kaiser (jennifer.kaiser@ce.gatech.edu)
Acetonitrile, Monoterpenes, Isoprene	Proton Transfer Reaction Mass Spectrometer - PTR-MS (20%)	NOAA CSL: Martin Graus (martin.graus@uibk.ac.at)
jNO_2, $jO_3 \rightarrow O(^1D)$	Filter Radiometers (10%)	NOAA CSL: Carsten Warneke (carsten.warneke@noaa.gov), John Holloway, Gerd Huebler
Organic aerosol	Compact Time-of-Flight Aerosol Mass Spectrometer - C-ToF AMS (30%)	NOAA CSL: Ann Middlebrook (Ann.M.Middlebrook@noaa.gov), Jin Liao, and Andre Welti
**NOMADSS**		
O_3, NO, NO_2	Chemiluminescence (NO: 10%, NO_2: 15%, O_3: 5%)	NCAR: Andrew J. Weinheimer (wein@ucar.edu), David J. Knapp, Deedee Montzka, Frank M. Flocke, Teresa .L. Campos, Geoff S. Tyndall
CO	Aero-Laser Vacuum Ultraviolet Fluorescence (3 ppbv + 3%)	NCAR: Teresa Campos (campos@ucar.edu), Frank Flocke, Michael Reeves, Daniel Stechman, and Meghan Stell
Acetonitrile, CH_2O	Trace Organic Gas Analyzer - TOGA (Acetonitrile: 40% or 2 pptv, CH_2O: 40% or 40 pptv)	NCAR: Eric Apel (apel@ucar.edu), Rebecca Hornbrook, Alan Hills, Daniel Riemer, Nicola Blake
Monoterpenes, Isoprene	Proton Transfer Reaction Mass Spectrometer - PTR-MS (20%)	NCAR and NOAA: Lisa Kaser (Lisa.Kaser@colorado.edu) and Bin Yuan (byuan@jnu.edu.cn)
jNO_2, $jO_3 \rightarrow O(^1D)$	High-performance instrumented airborne platform for environmental research Airborne Radiation Package (HARP) - Actinic Flux (jNO_2: 12%, jO_3: 25%)	NCAR: Samuel R. Hall (halls@ucar.edu) and and Kirk Ullmann
**DISCOVER-AQ (TX & CA)**		
O_3, NO, NO_2	Chemiluminescence (O_3: 0.1 ppbv + 5%, NO: 10 pptv + 10%, NO_2: 20 pptv + 10%)	NCAR: Andrew J. Weinheimer (wein@ucar.edu), David J. Knapp, Deedee Montzka
CO	Diode laser spectrometer - DACOM (2% or 2 ppbv)	NASA LaRC: Glenn S. Diskin (glenn.s.diskin@nasa.gov)
CH_2O	Difference Frequency Generation Absorption Spectroscopy - DFGAS (quadrature addition of 20 pptv & 4%)	The University of Colorado at Boulder - INSTAAR (Institute of Arctic and Alpine Research): Alan Fried (alan.fried@colorado.edu) and James Walega
Acetonitrile, Monoterpenes, Isoprene	Proton Transfer Reaction Time of Flight Mass Spectrometer - PTR-ToF-MS (reported in data file)	University of Innsbruck: Armin Wisthaler (armin.wisthaler@uibk.ac.at)
jNO_2	Filter Radiometers	NASA LaRC: John Barrick (john.d.barrick@nasa.gov) and Ali Aknan

Acronyms are defined in Table S3. **For more information** about the campaigns including instrumentation refer to Warneke et al. (2016) and `https://csl.noaa.gov/groups/csl7/measurements/2013senex/` for SENEX; Carlton et al. (2018) and `https://data.eol.ucar.edu/dataset/373.046` for NOMADSS; and Crawford and Pickering (2014) and DOI: 10.5067/Aircraft/DISCOVER-AQ/Aerosol-TraceGas for DISCOVER-AQ

Table S5. Differences between Model Simulations Varying Chemistry or Resolution

Region	Chemistry (TS2.1 - TS1)		Horizontal Resolution (~14 km - ~111 km)	
	~14 km	~111 km	TS1	TS2.1
**MDA8 Ozone (ppbv)**				
SE	-3.150	-2.864	0.932	0.635
TX	-2.432	-0.793	0.008	-1.493
CA	-1.687	-1.868	1.956	2.197
**NO_x (ppbv)**				
SE	0.054	0.089	-0.049	-0.080
TX	-0.225	0.065	1.819	1.529
CA	0.076	0.111	-0.829	-0.873
**Formaldehyde (ppbv)**				
SE	0.0115	0.110	1.276	1.168
TX	0.022	0.036	0.231	0.213
CA	-0.165	-0.297	-0.048	0.094
**Organic Aerosol (μg m^{-3})**				
SE	-2.062	-1.944	0.756	0.631
TX	-0.569	-0.434	0.166	0.094
CA	-1.053	-1.041	-1.267	-1.260
**CO (ppbv)**				
SE	2.306	3.432	19.603	18.514
TX	-0.886	2.494	17.921	14.453
CA	-0.289	-1.680	-15.456	-14.098
**Isoprene (ppbv)**				
SE	-0.039	-0.027	2.359	2.347
TX	0.095	0.016	1.404	1.477
CA	0.014	0.003	0.545	0.556
**Isoprene hydroxy hydroperoxide (ppbv)**				
SE	-0.137	-0.108	0.180	0.151
TX	-0.057	-0.039	0.085	0.072
CA	-0.027	-0.014	0.065	0.052
**Isoprene hydroxy nitrate (ppbv)**				
SE	0.004	0.004	0.011	0.011
TX	0.002	0.004	0.015	0.012
CA	0.012	0.007	0.019	0.023
**HO_x (pptv)**				
SE	-2.746	-2.234	1.286	0.774
TX	-1.832	-1.297	-0.214	-0.665
CA	-1.980	-1.406	0.592	0.093
**Methyl vinyl ketone (pptv)**				
SE	0.049	0.108	0.373	0.312
TX	0.102	0.094	0.167	0.163
CA	0.095	0.054	0.145	0.182
**Methacrolein (pptv)**				
SE	-0.159	-0.091	0.360	0.292
TX	-0.035	-0.008	0.139	0.114
CA	-0.002	-0.003	0.105	0.106

*Area-weighted average differences for August 2013 calculated for each region in Figure S1: Southeast US (SE), Houston, Texas (TX), and California (CA)

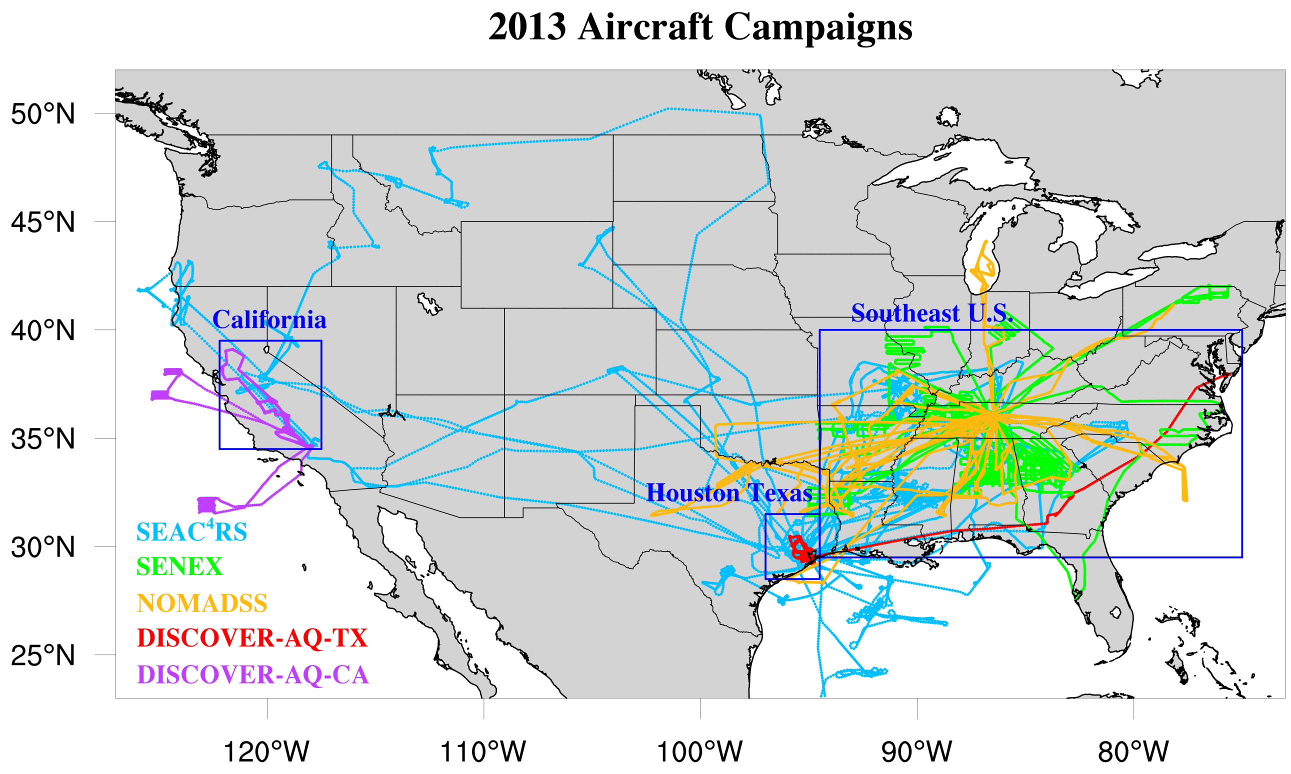

Figure S1. Flight tracks from the five 2013 aircraft campaigns used to evaluate CESM/CAM-chem-SE. Only data within the following representative regions shown in blue boxes are used in the analysis: Southeast US [94.5 - 75° W, 29.5 - 40° N] for SEAC^{4}RS, SENEX, and NOMADSS; Houston, Texas, [97 - 94.5° W, 28.5 - 31.5° N] for DISCOVER-AQ-TX; and California [122.2 - 117.5° W, 34.5 - 39.5° N] for DISCOVER-AQ-CA.

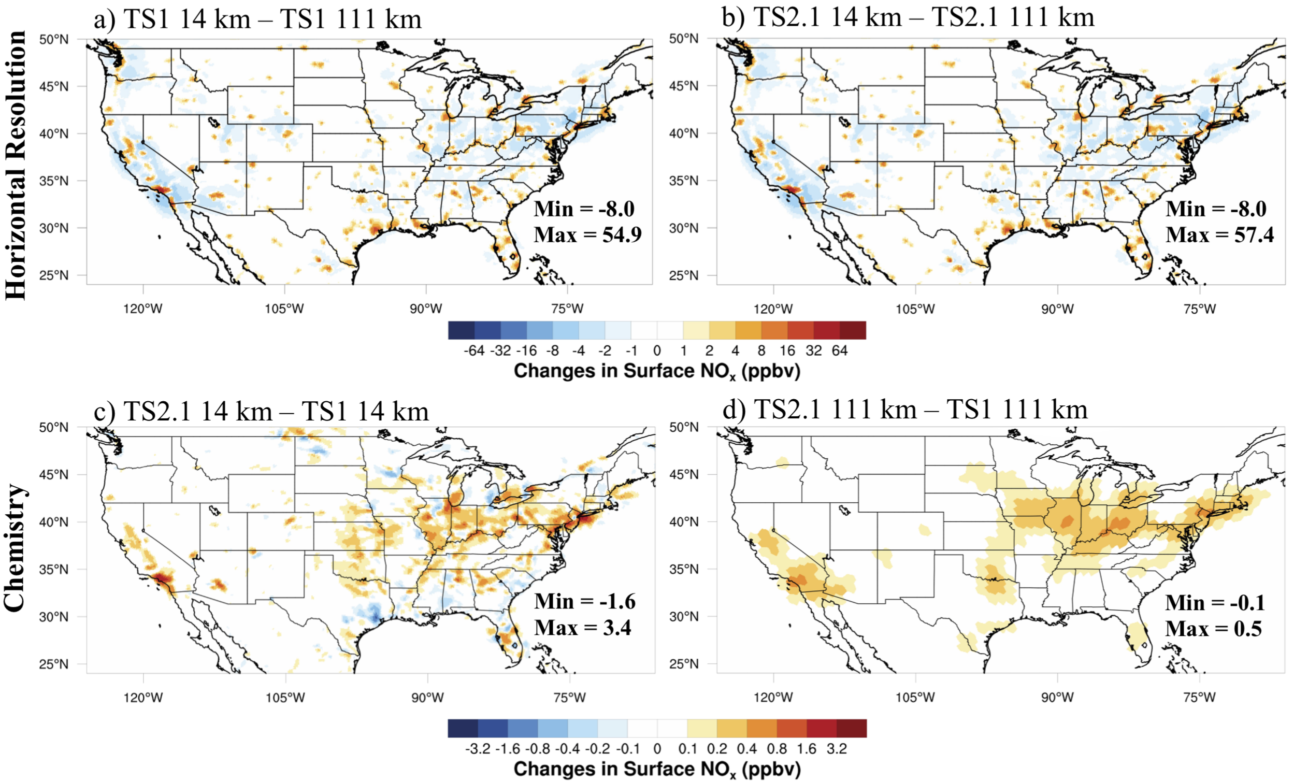

Figure S2. August 2013 average surface nitrogen oxides (NO_x) differences between different horizontal resolutions with chemistry fixed (a & b) and different chemical mechanisms with horizontal resolution fixed (c & d).

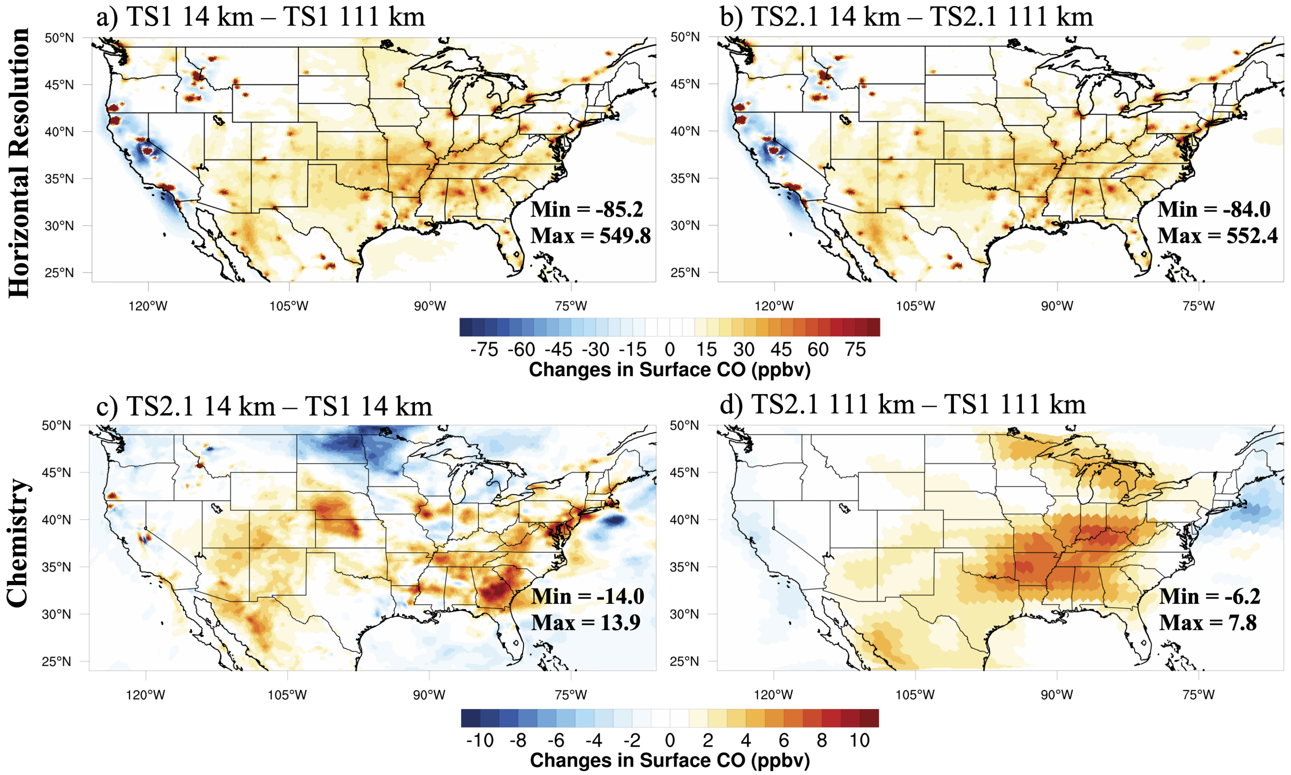

Figure S3. August 2013 average surface carbon monoxide (CO) differences between different horizontal resolutions with chemistry fixed (a & b) and different chemical mechanisms with horizontal resolution fixed (c & d).

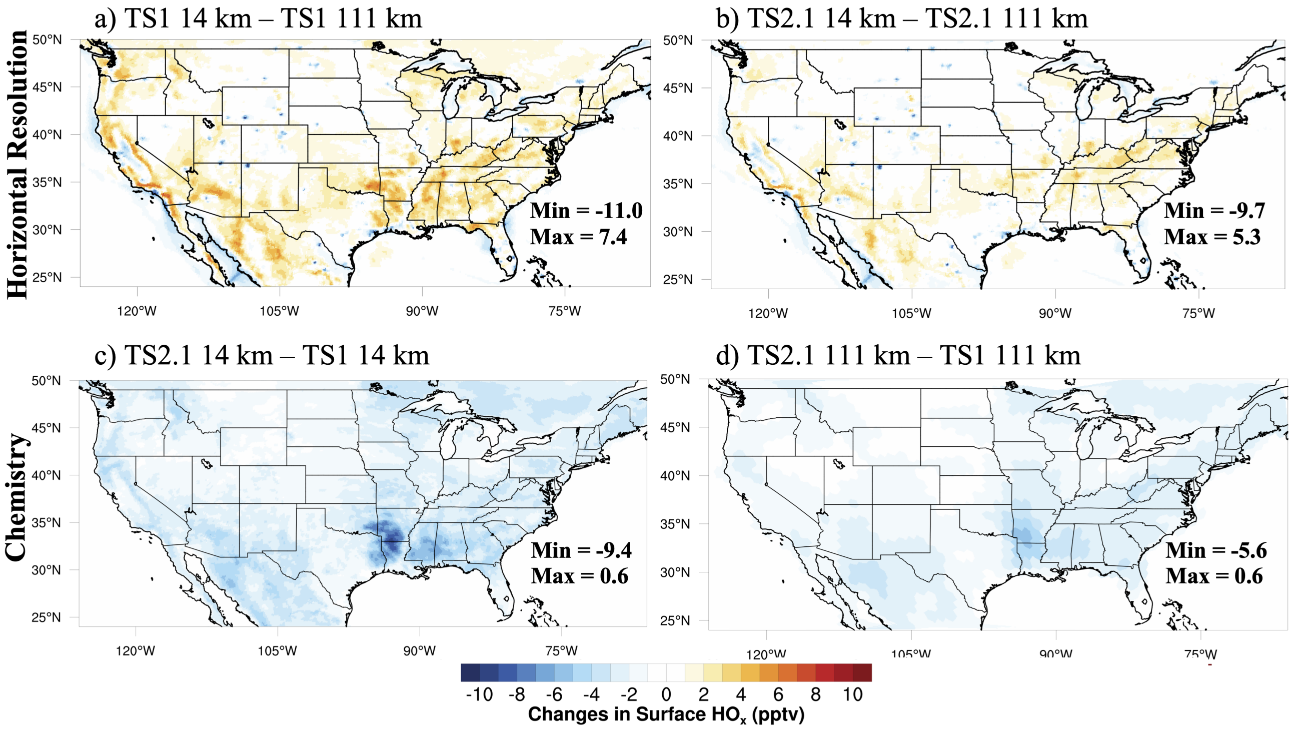

Figure S4. August 2013 average surface HO_x (hydroxyl + hydroperoxyl radicals) differences between different horizontal resolutions with chemistry fixed (a & b) and different chemical mechanisms with horizontal resolution fixed (c & d).

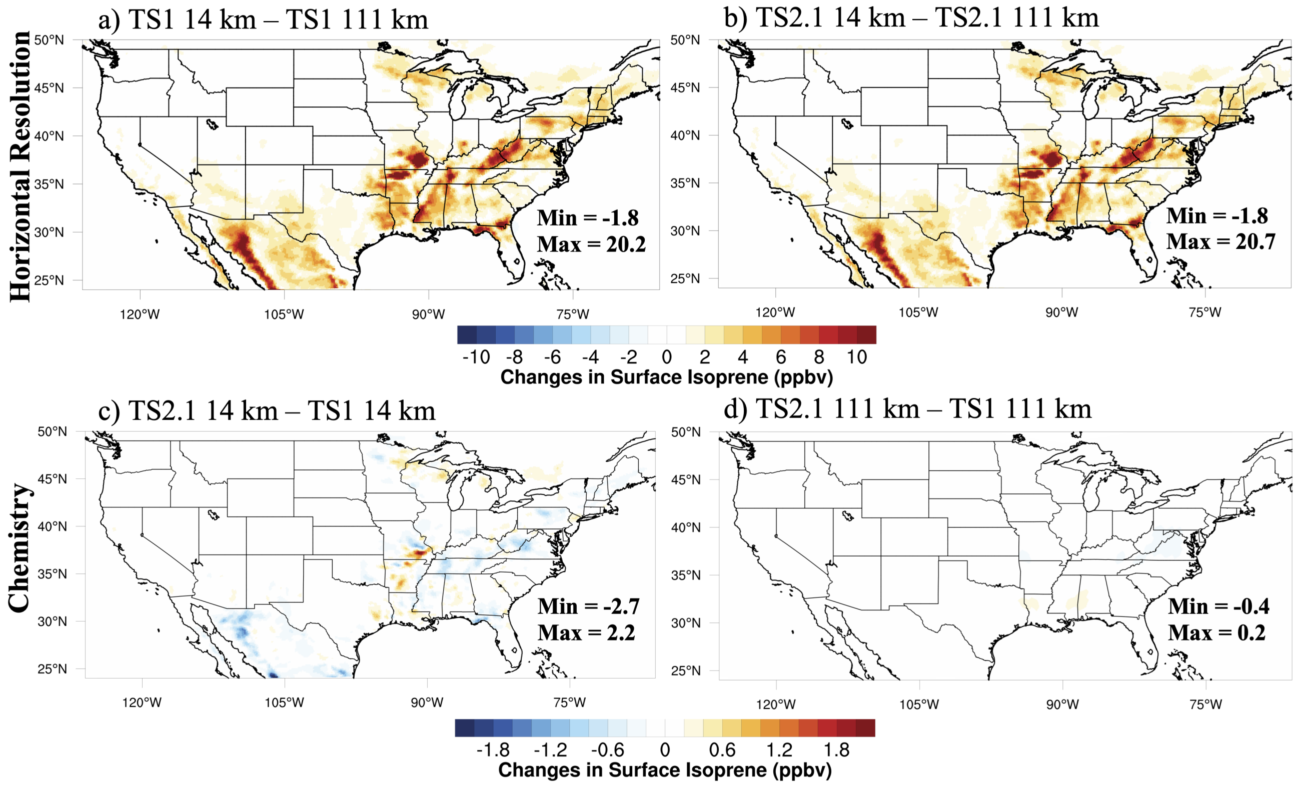

Figure S5. August 2013 average surface isoprene differences between different horizontal resolutions with chemistry fixed (a & b) and different chemical mechanisms with horizontal resolution fixed (c & d).

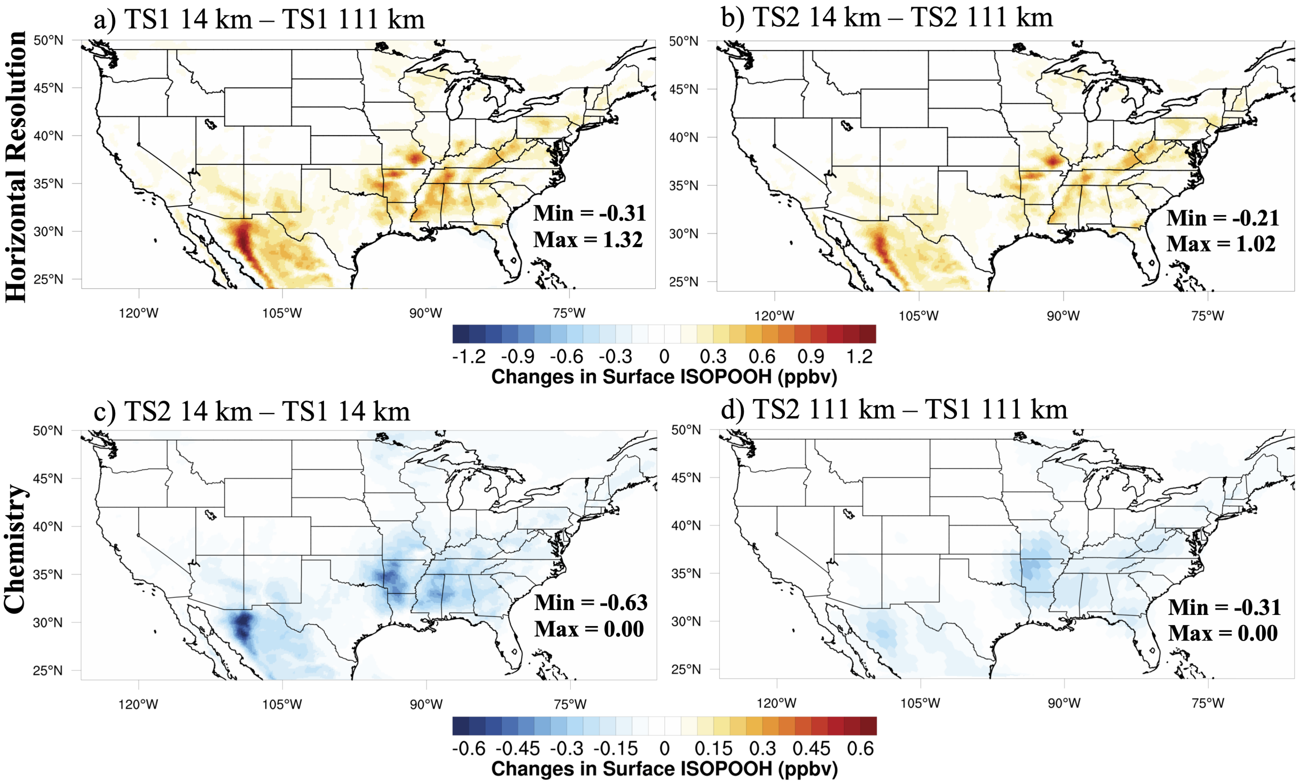

Figure S6. August 2013 average surface isoprene hydroxy hydroperoxide (ISOPOOH) differences between different horizontal resolutions with chemistry fixed (a & b) and different chemical mechanisms with horizontal resolution fixed (c & d).

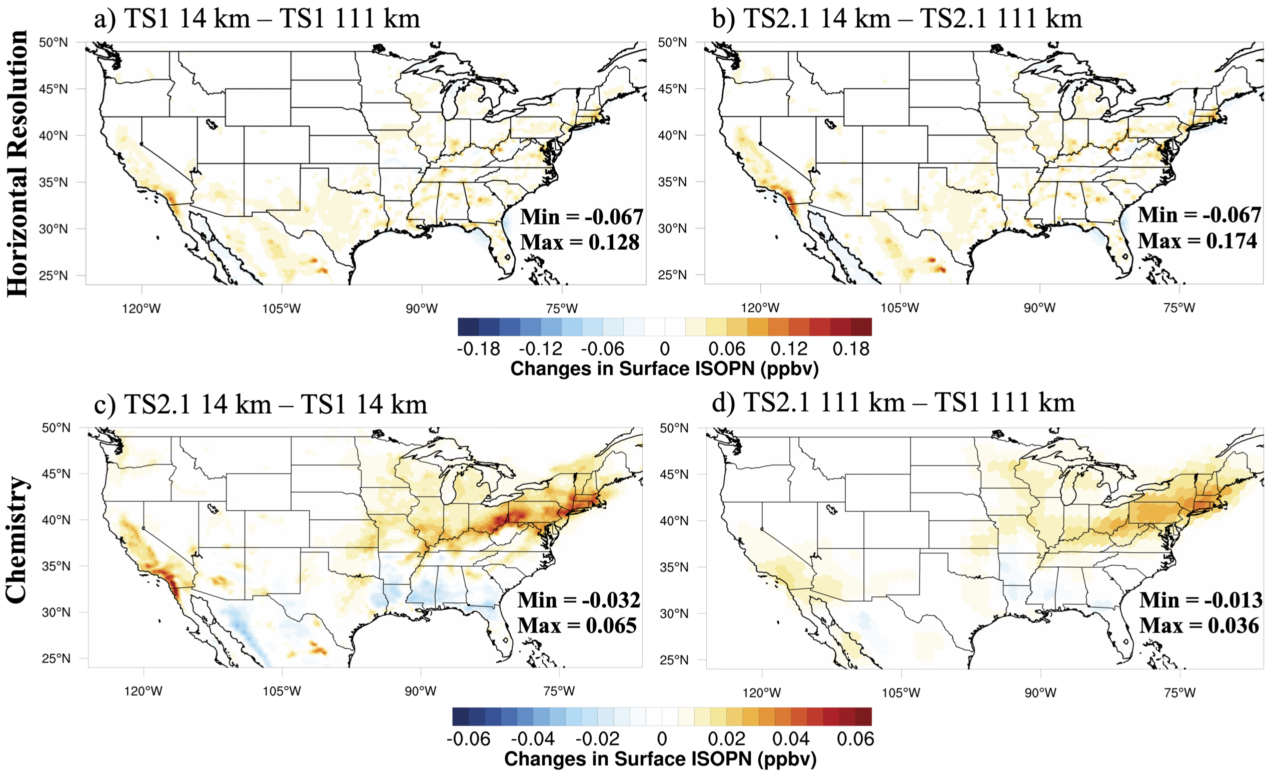

Figure S7. August 2013 average surface isoprene hydroxy nitrate (ISOPN) differences between different horizontal resolutions with chemistry fixed (a & b) and different chemical mechanisms with horizontal resolution fixed (c & d).

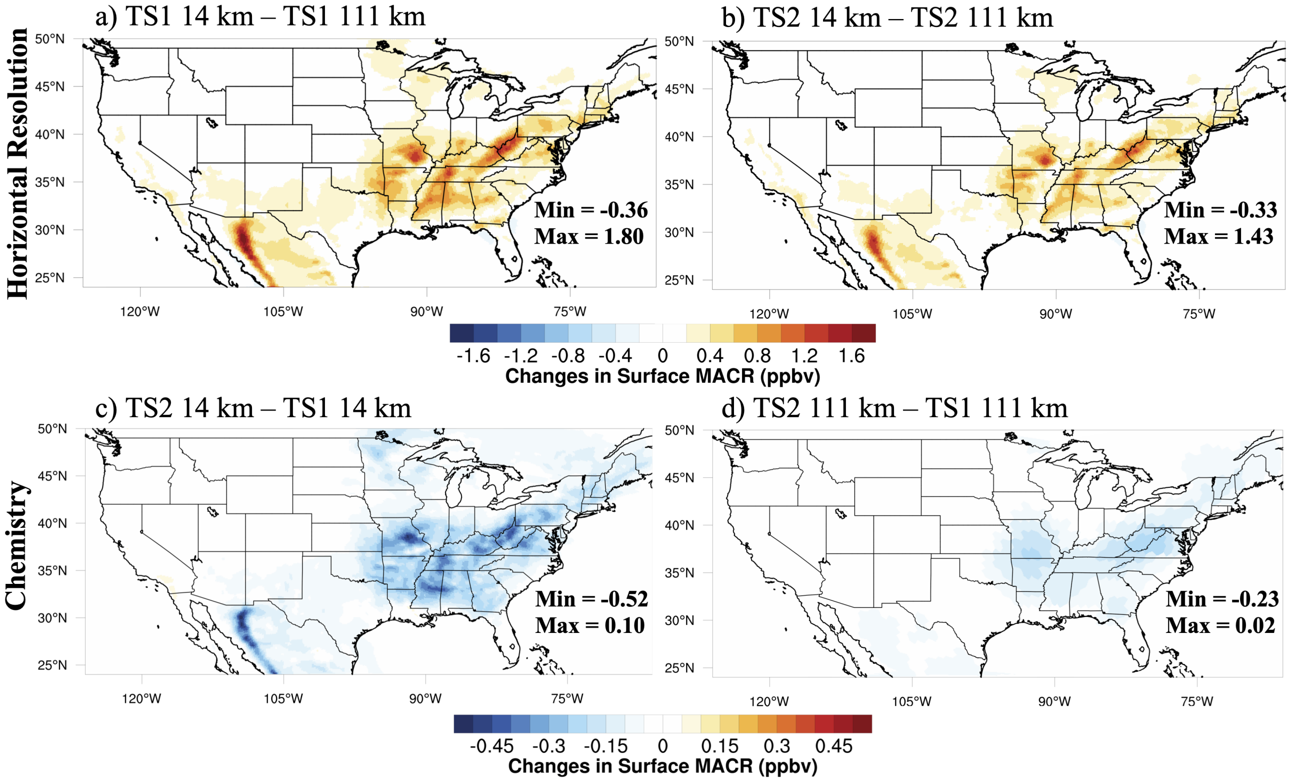

Figure S8. August 2013 average surface methacrolein (MACR) differences between different horizontal resolutions with chemistry fixed (a & b) and different chemical mechanisms with horizontal resolution fixed (c & d).

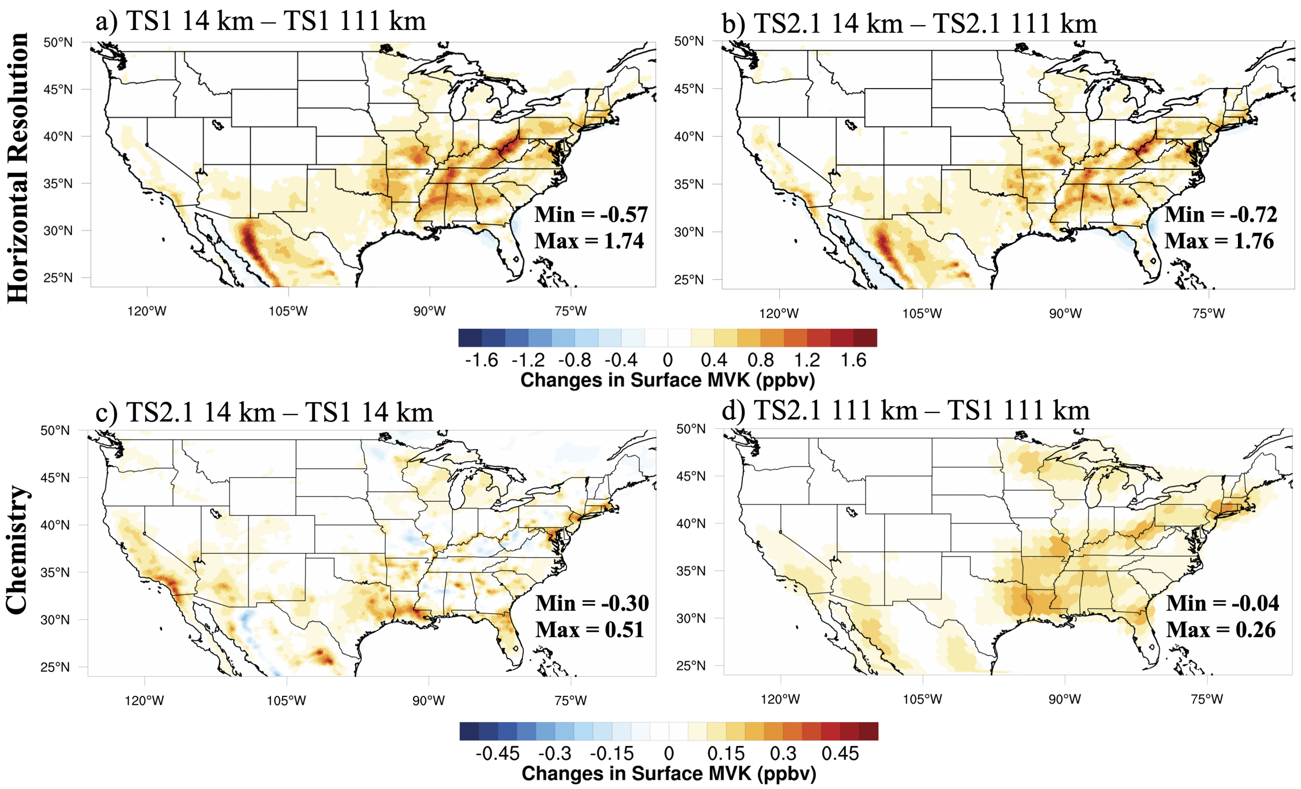

Figure S9. August 2013 average surface methyl vinyl ketone (MVK) differences between different horizontal resolutions with chemistry fixed (a & b) and different chemical mechanisms with horizontal resolution fixed (c & d).

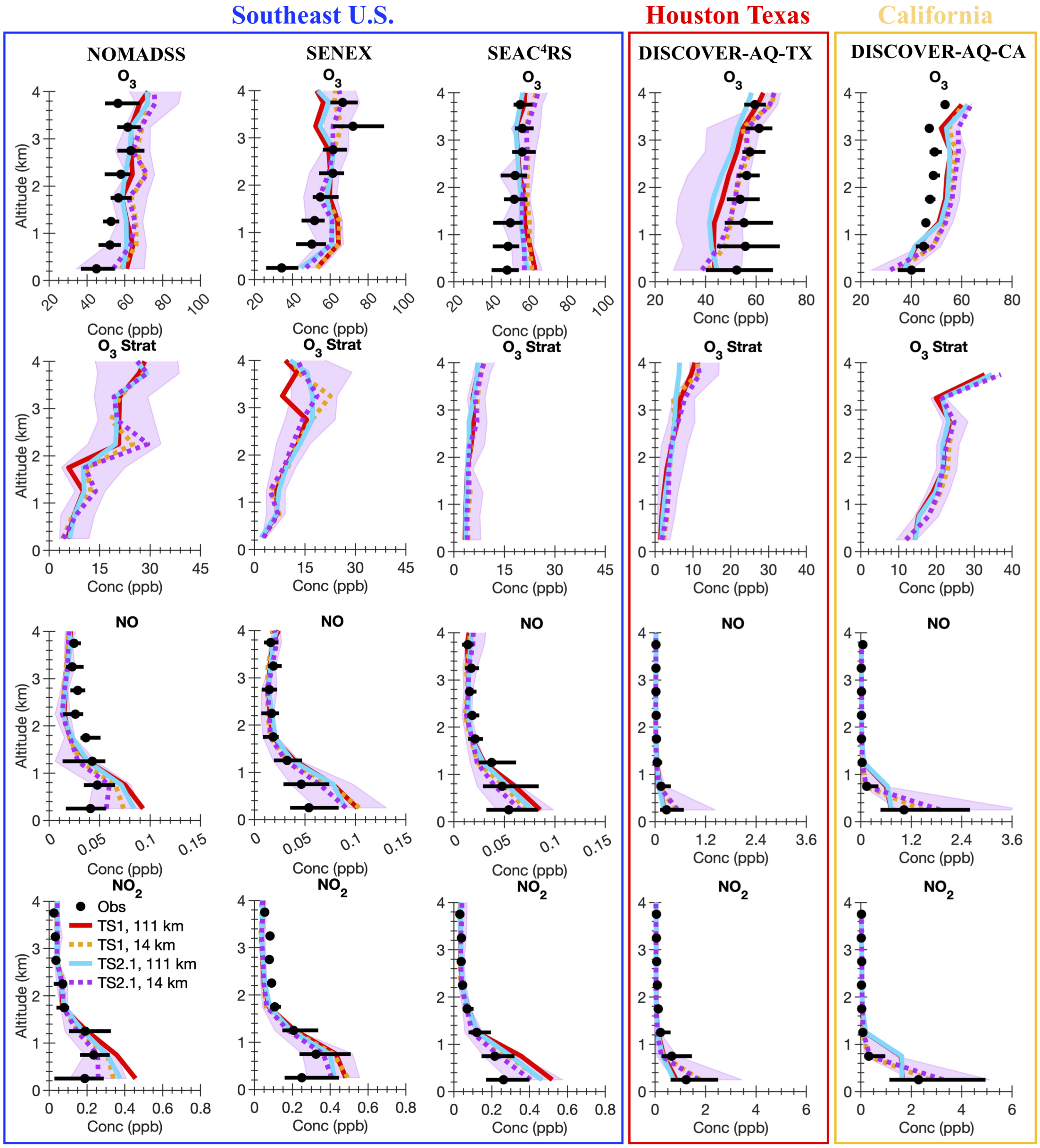

Figure S10. Median vertical profile plots of the five aircraft campaigns for observations (black markers) and the model simulations (colored lines). The black horizontal lines and the purple shading show the 25th and 75th percentiles for the observations and the TS2.1, 14 km model simulation, respectively. Abbreviations are ozone (O_3), stratospheric ozone tracer (O_3 Strat), nitrogen oxide (NO), and nitrogen dioxide (NO_2). This figure is similar to Figure 4 in the main text, but is the only figure in the main text or SI where no horizontal interpolation is applied when processing the flight tracks and instead the grid cell closest in distance to each observational point is selected.

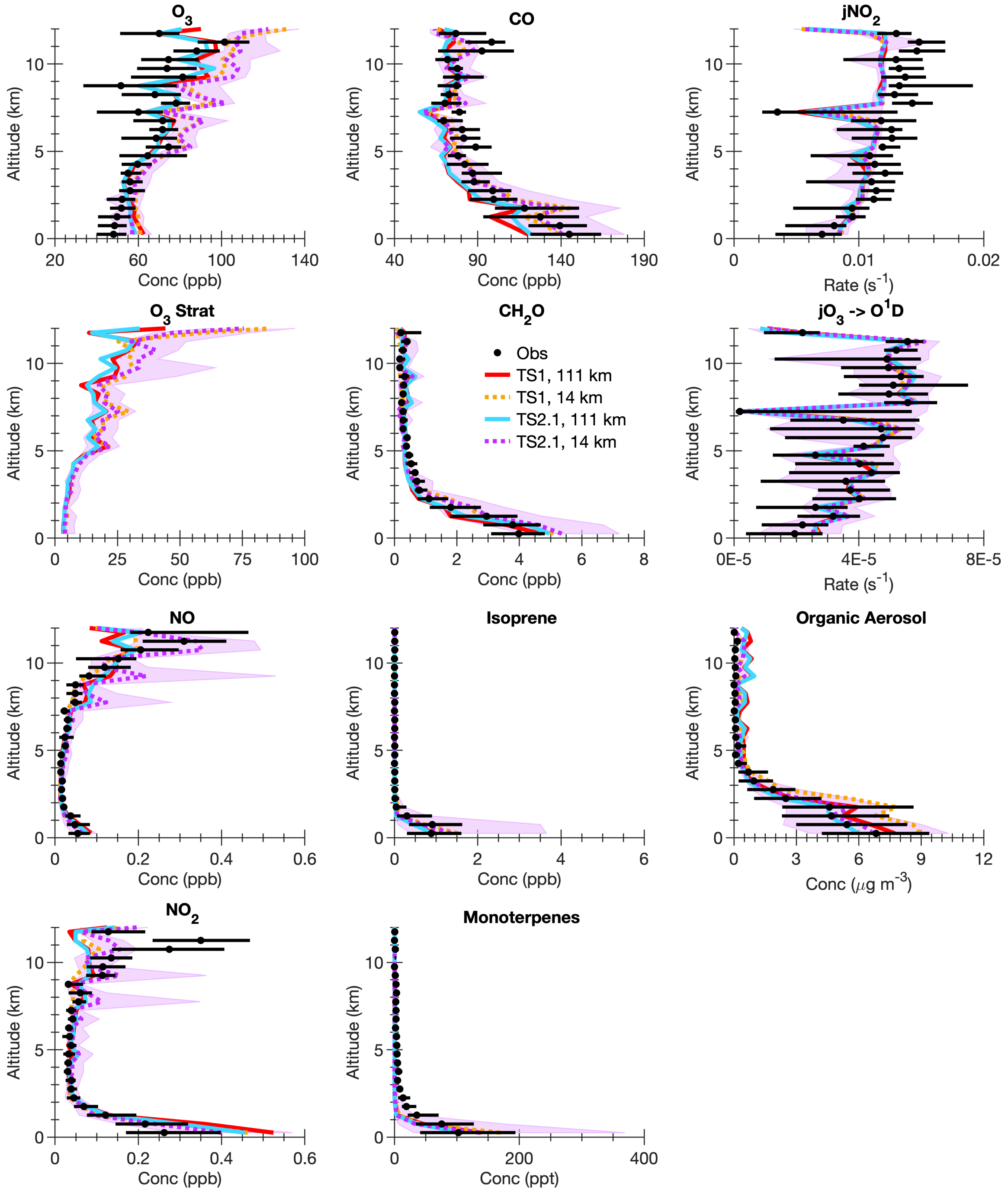

Figure S11. Median vertical profile plots for the SEAC[4]RS campaign for observations (black markers) and the model simulations in colored lines. The black horizontal lines and the purple shading show the 25th and 75th percentiles for the observations and the TS2.1, ∼14 km model simulation, respectively. Abbreviations are ozone (O_3), stratospheric ozone tracer (O_3 Strat), nitrogen oxide (NO), nitrogen dioxide (NO_2), carbon monoxide (CO), and formaldehyde (CH_2O). Figure is similar to Figures 4-7 in the main text, but extended to 12 km pressure altitude.

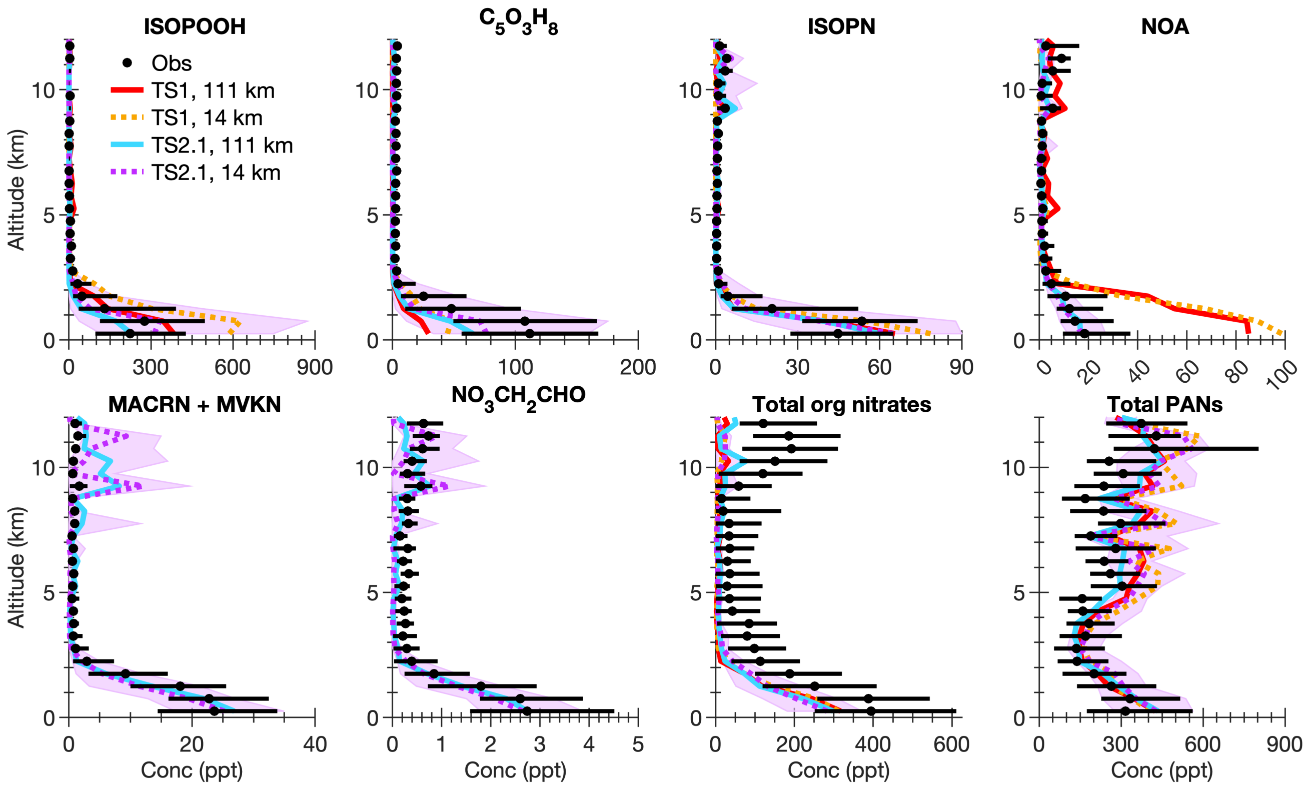

Figure S12. Identical to Figure S11, but for isoprene hydroxy hydroperoxide (ISOPOOH), isoprene hydroperoxy aldehyde (HPALD), isoprene hydroxy nitrate (ISOPN), propanone nitrate (NOA), methacrolein and methyl vinyl ketone hydroxy nitrates (MACRN + MVKN), ethanal nitrate (NO_3CH_2CHO), total organic nitrates, and total peroxy acyl nitrates (PANs). Figure is identical to Figure 8 in the main text, but extended to 12 km pressure altitude.

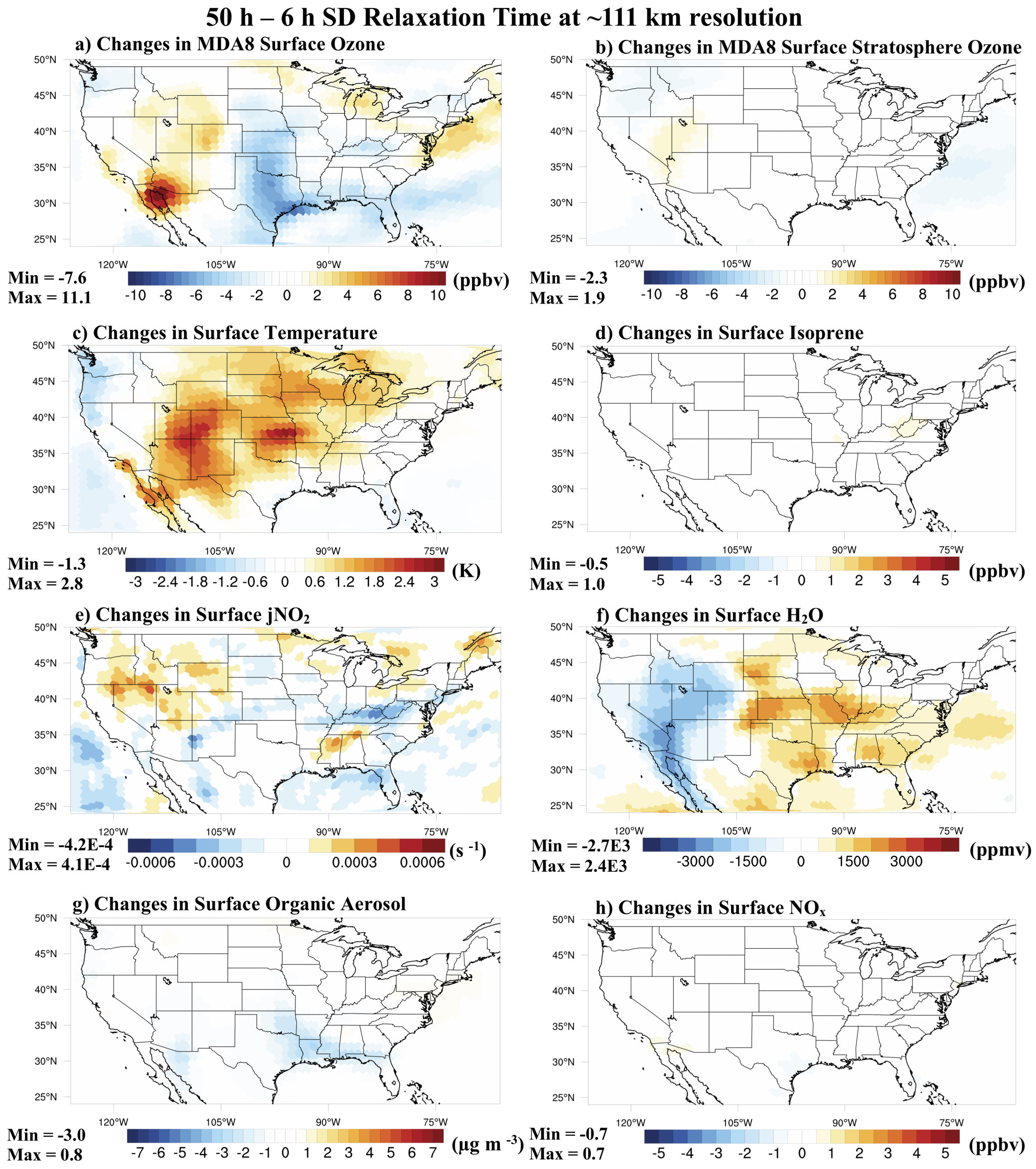

Figure S13. Changes at the surface caused by using a 50-h minus a 6-h specified dynamics (SD) relaxation time with the TS2.1 chemical mechanism at ∼111 km horizontal resolution for (a) MDA8 surface ozone, (b) MDA8 surface stratospheric ozone tracer, (c) temperature, (d) isoprene, (e) photolysis rate of NO_2, (f) water mixing ratio, (g) organic aerosol, and (h) NO_x.

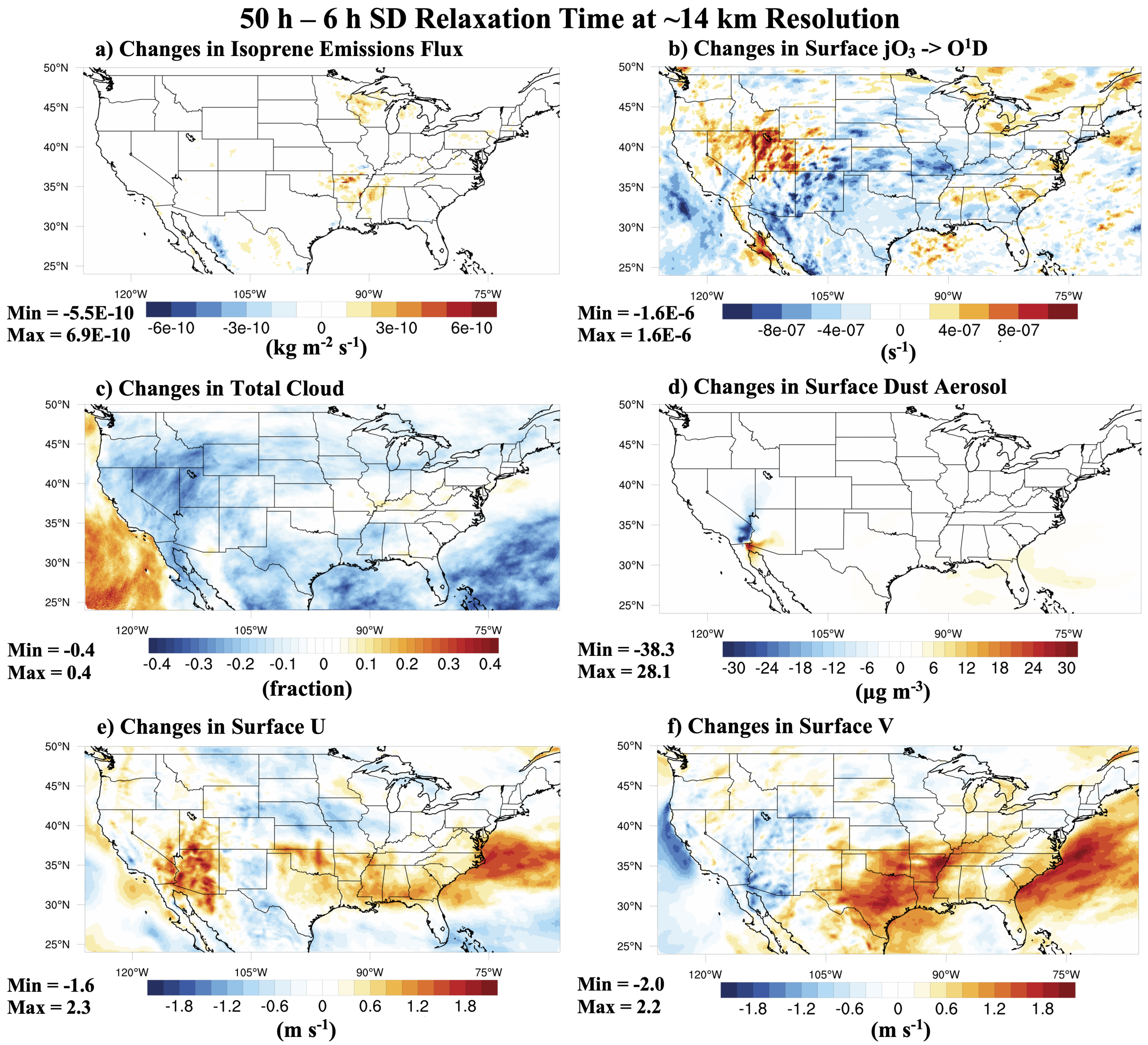

Figure S14. Changes at the surface caused by using a 50-h minus a 6-h specified dynamics (SD) relaxation time with the TS2.1 chemical mechanism at ∼14 km horizontal resolution for (a) isoprene emissions flux, (b) photolysis rate of O_3, (c) total cloud fraction, (d) dust aerosol, (e) zonal wind velocity - U, (f) meridional wind velocity - V.

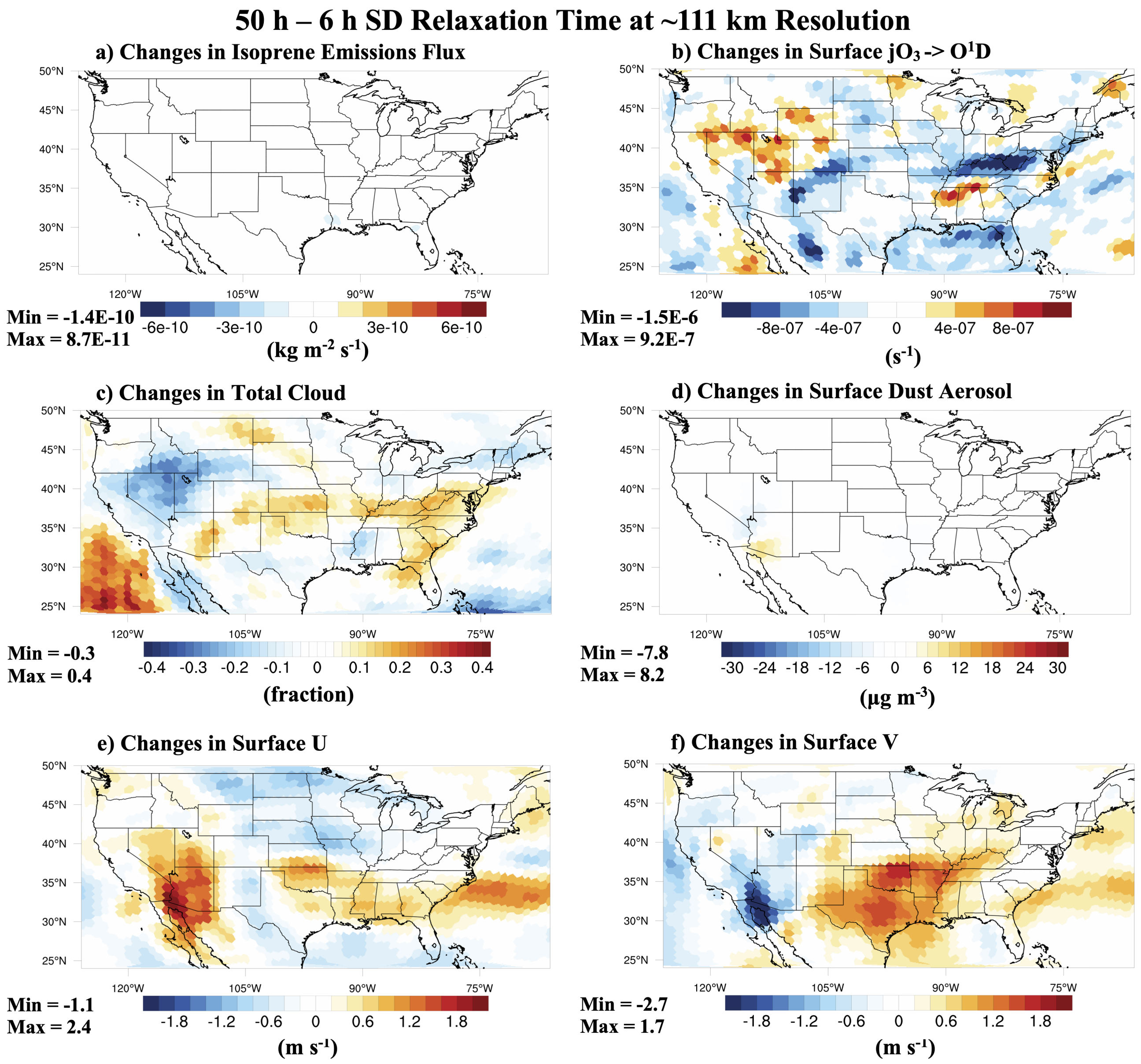

Figure S15. Changes at the surface caused by using a 50-h minus a 6-h specified dynamics (SD) relaxation time with the TS2.1 chemical mechanism at ~111 km horizontal resolution for (a) isoprene emissions flux, (b) photolysis rate of O_3, (c) total cloud fraction, (d) dust aerosol, (e) zonal wind velocity - U, (f) meridional wind velocity - V.

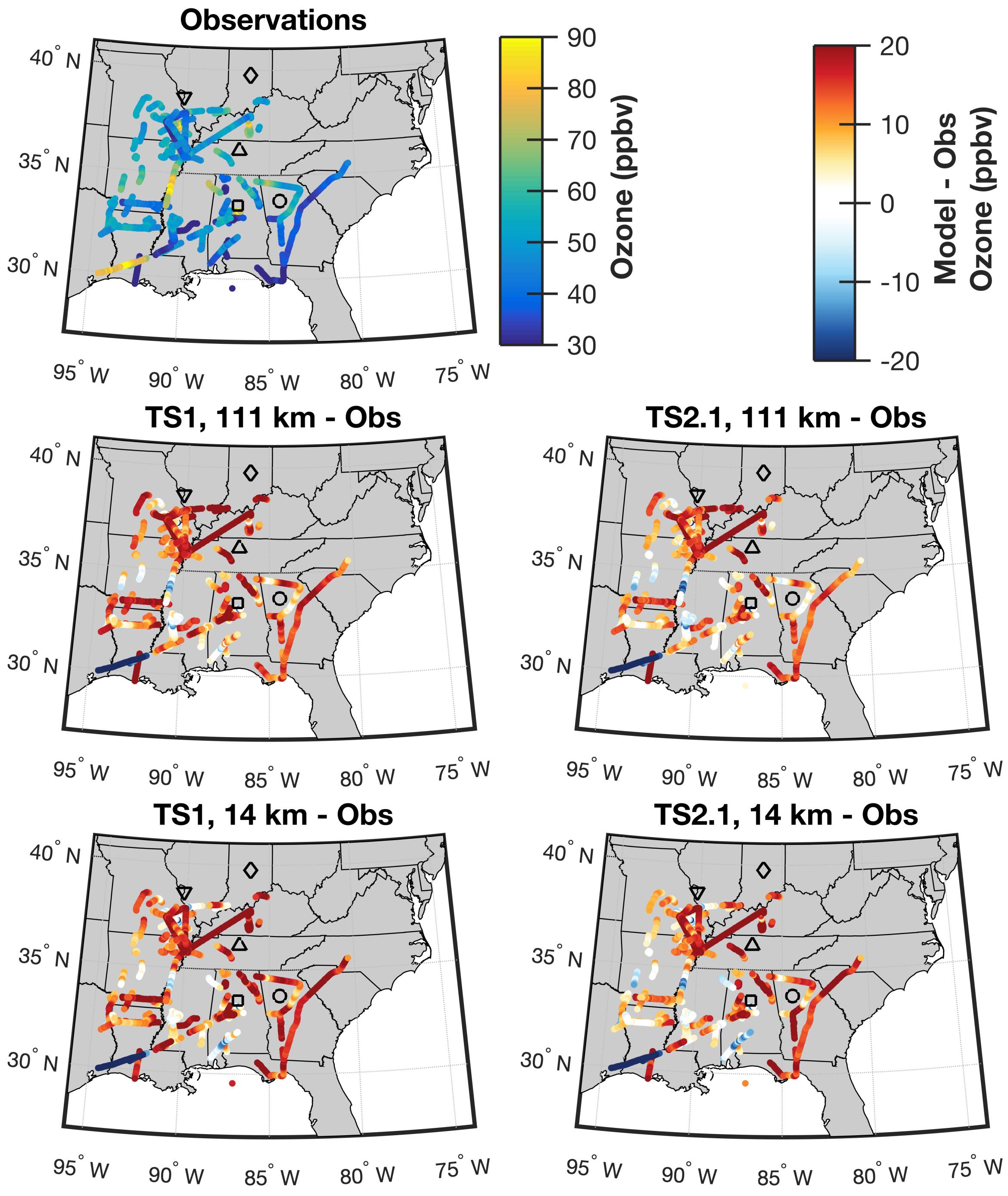

Figure S16. Observations of ozone (top panel) followed by differences between model - observations (obs) for ozone along the SEAC^{4}RS flight tracks below 1 km pressure altitude for each of the model simulations performed evaluating two different chemical mechanisms of varying complexity (TS1 & TS2.1) and two different horizontal resolutions (∼111 km & ∼14 km). Markers indicate cities targeted during the SENEX campaign: Atlanta (circle), Birmingham (square), Nashville (upward triangle), St. Louis (downward triangle), and Indianapolis (diamond).

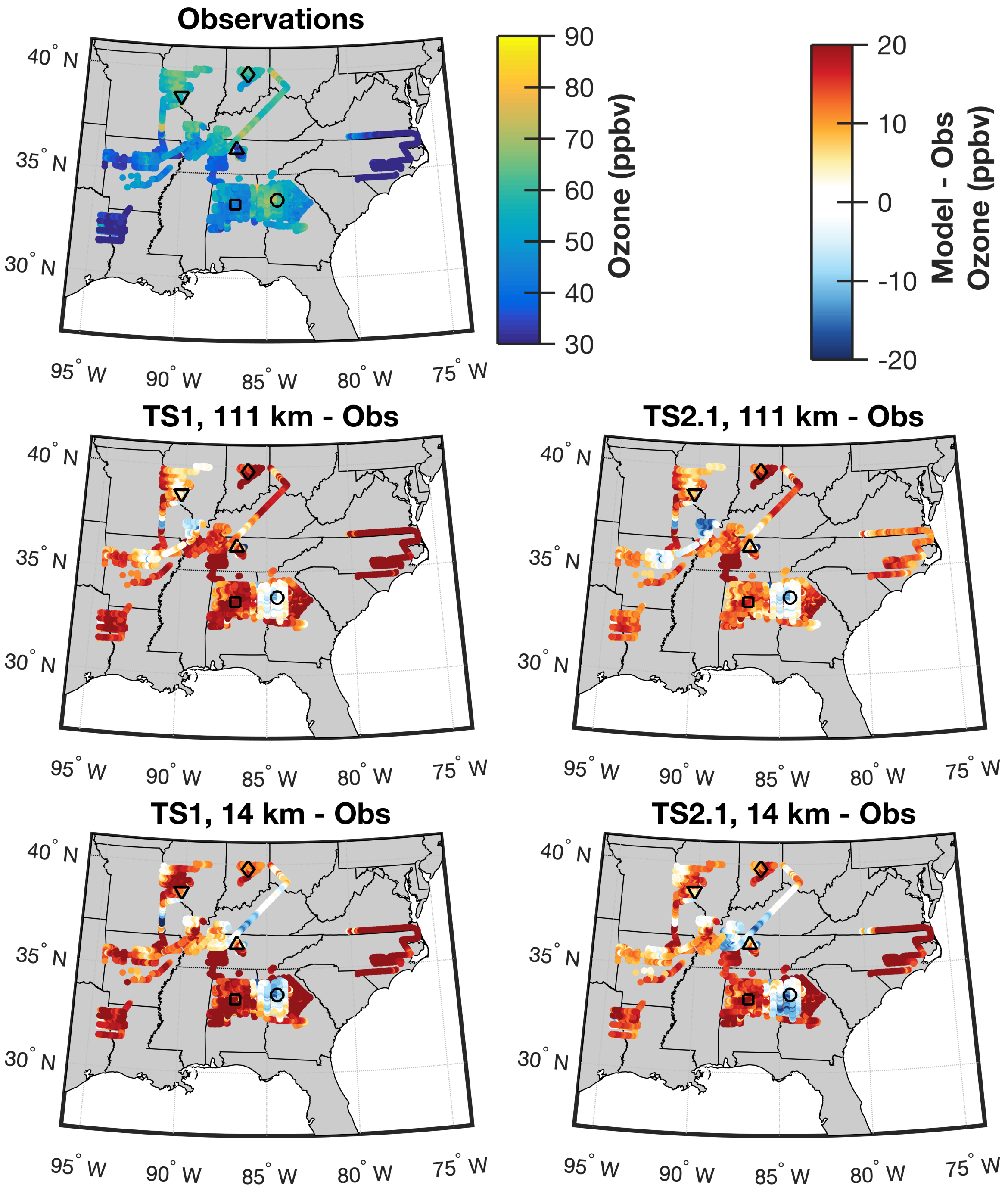

Figure S17. Identical to Figure S16, but for ozone along the SENEX flight tracks.

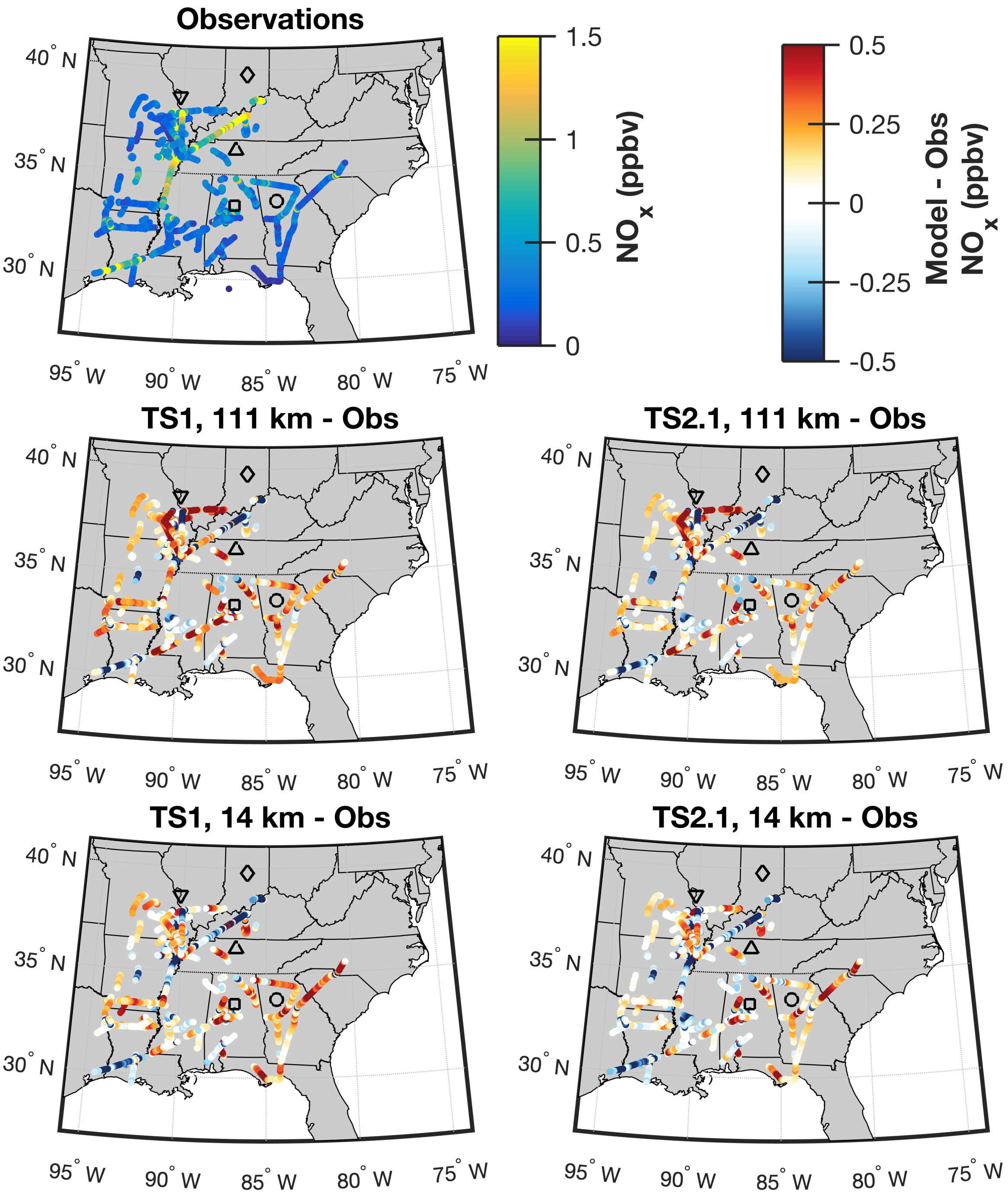

Figure S18. Identical to Figure S16, but for NO_x along the SEAC[4]RS flight tracks.

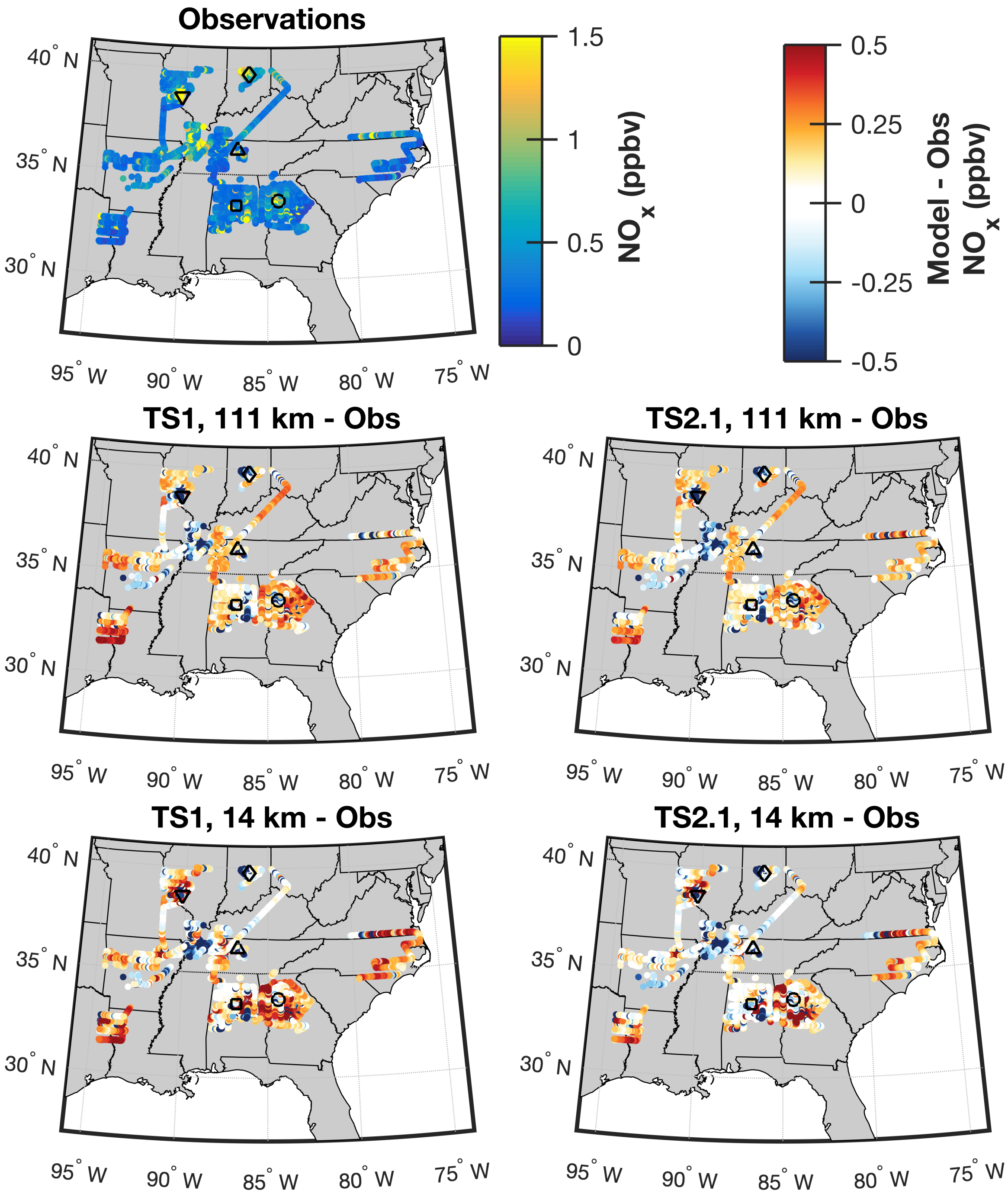

Figure S19. Identical to Figure S16, but for NO_x along the SENEX flight tracks.

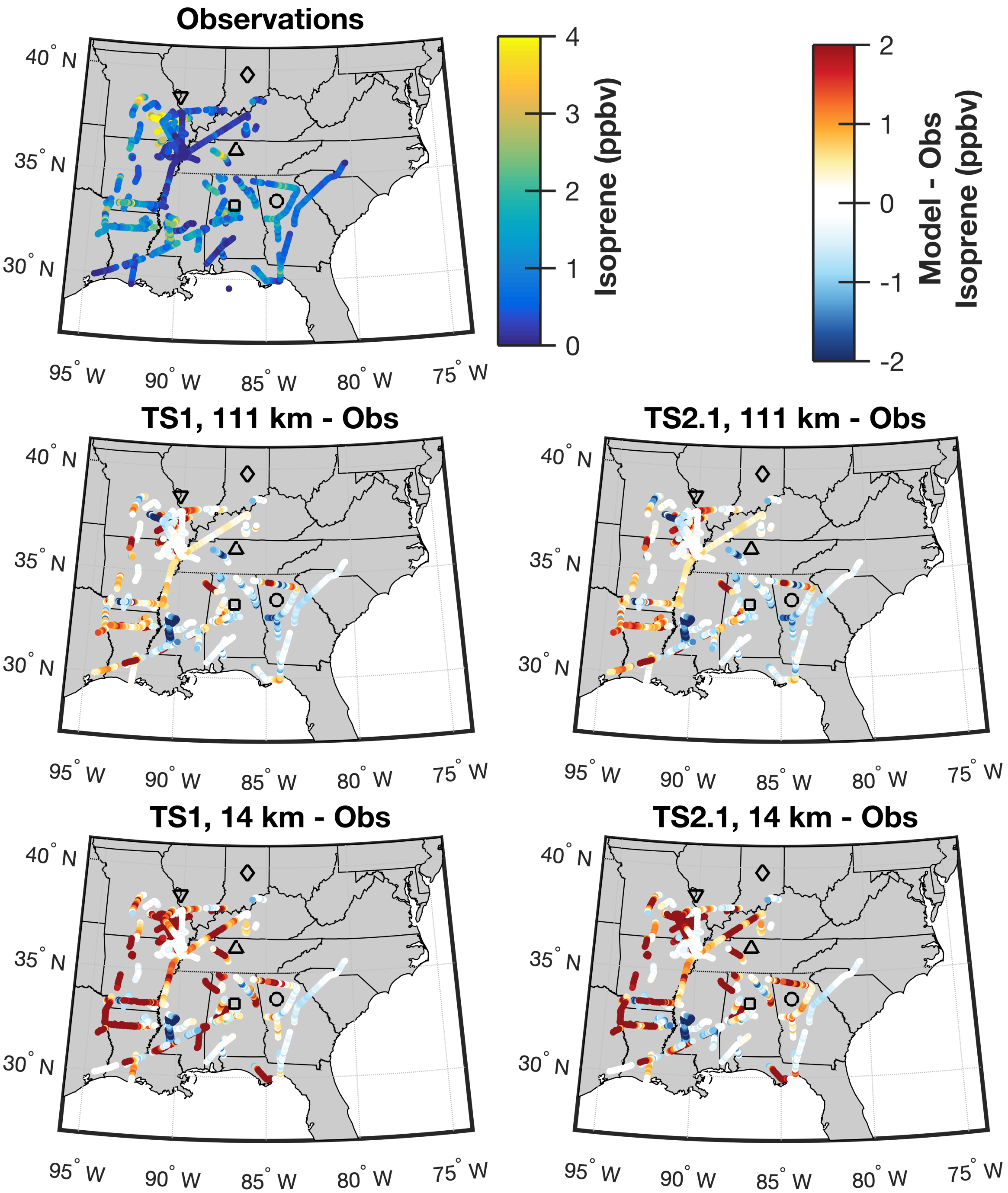

Figure S20. Identical to Figure S16, but for isoprene along the SEAC^{4}RS flight tracks.

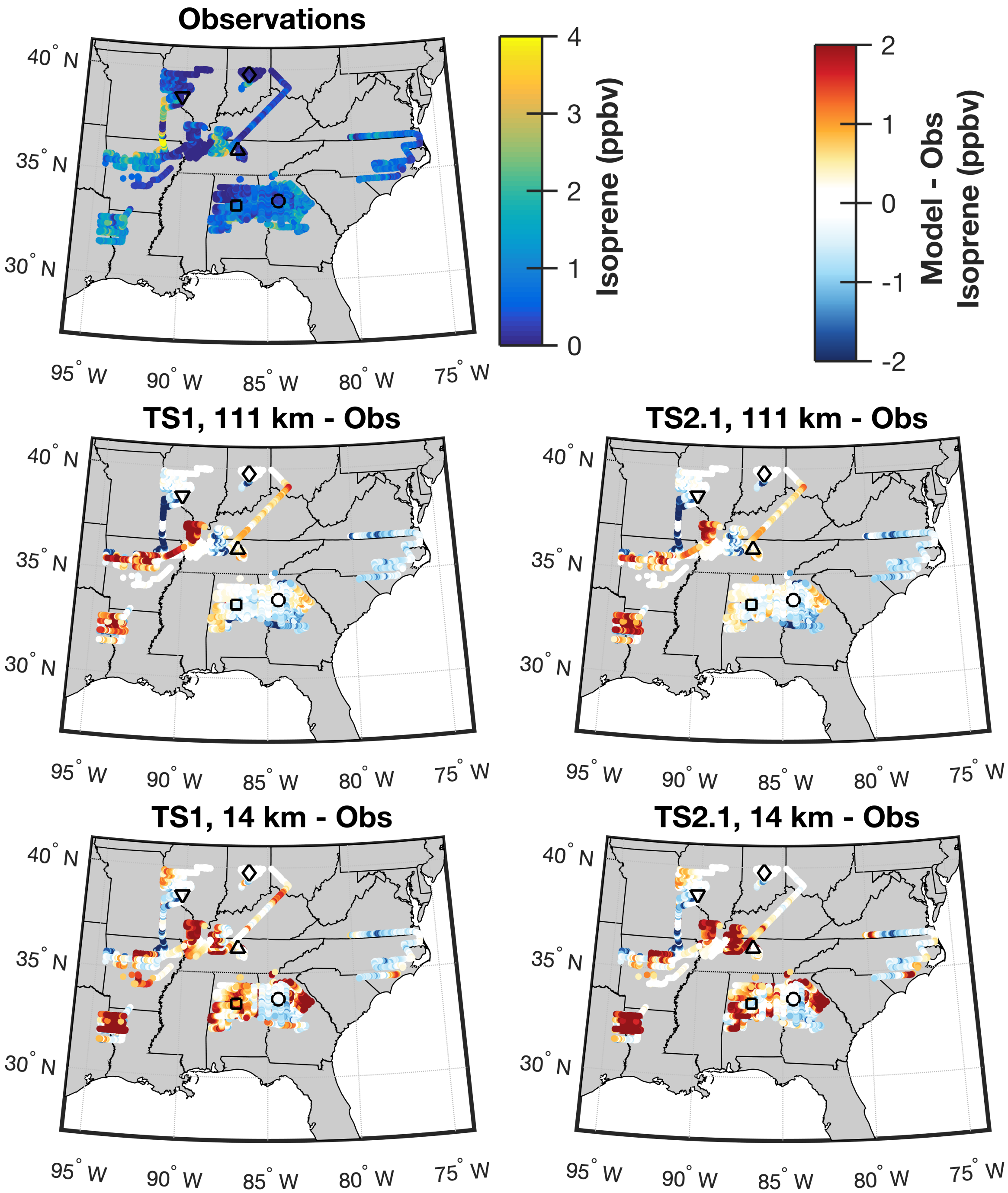

Figure S21. Identical to Figure S16, but for isoprene along the SENEX flight tracks.

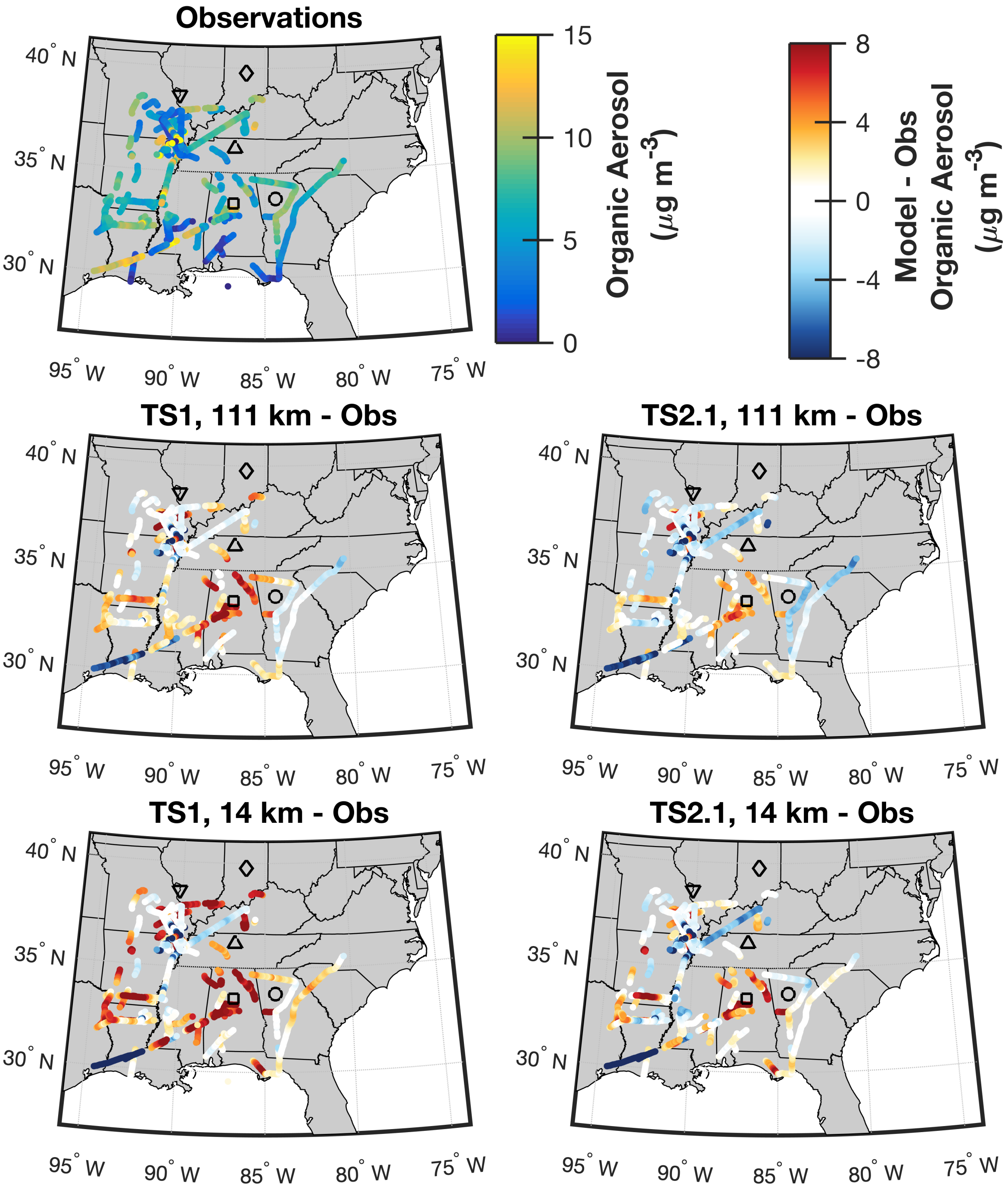

Figure S22. Identical to Figure S16, but for organic aerosol along the SEAC^{4}RS flight tracks.

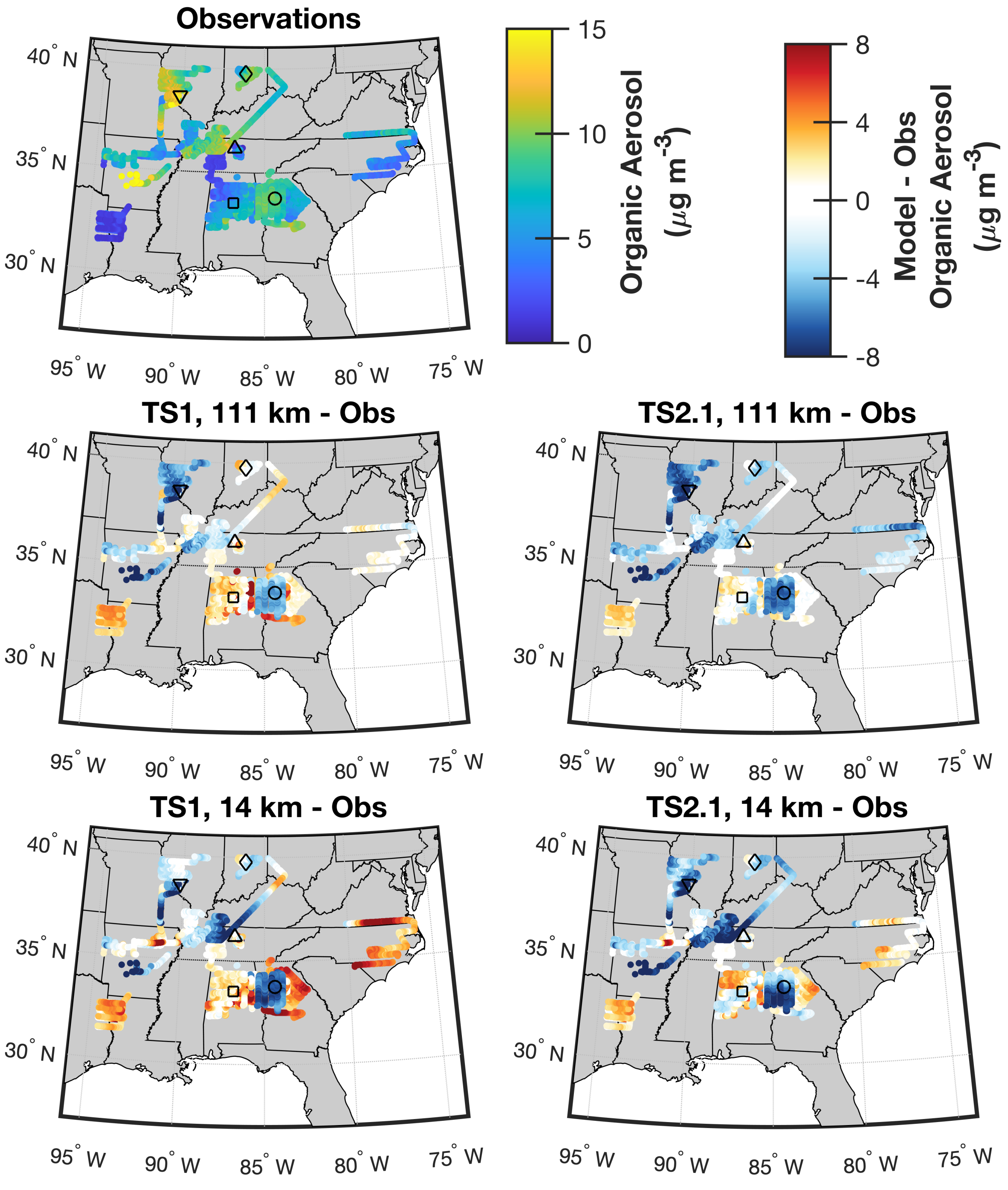

Figure S23. Identical to Figure S16, but for organic aerosol along the SENEX flight tracks.
